# Inhibition of the K_Ca_2 potassium channel in atrial fibrillation: a randomized phase 2 trial

**DOI:** 10.1038/s41591-023-02679-9

**Published:** 2023-12-13

**Authors:** Anders G. Holst, János Tomcsányi, Birgitte Vestbjerg, Morten Grunnet, Ulrik S. Sørensen, Jonas G. Diness, Bo H. Bentzen, Nils Edvardsson, Stefan H. Hohnloser, Deepak L. Bhatt, Paul Dorian

**Affiliations:** 1 Acesion Pharma, Copenhagen, Denmark; 2 Cardiology Department, St. John of God Hospital, Budapest, Hungary; 3 Department of Molecular and Clinical Medicine/Cardiology, Institute of Medicine, Sahlgrenska Academy, University of Gothenburg, Gothenburg, Sweden; 4 Department of Cardiology, J. W. Goethe University, Frankfurt, Germany; 5 Mount Sinai Heart, Icahn School of Medicine at Mount Sinai, New York, NY USA; 6 Department of Medicine, Division of Cardiology, University of Toronto, St. Michael’s Hospital, Toronto, Ontario Canada

**Keywords:** Atrial fibrillation, Drug development

## Abstract

Existing antiarrhythmic drugs to treat atrial fibrillation (AF) have incomplete efficacy, contraindications and adverse effects, including proarrhythmia. AP30663, an inhibitor of the K_Ca_2 channel, has demonstrated AF efficacy in animals; however, its efficacy in humans with AF is unknown. Here we conducted a phase 2 trial in which patients with a current episode of AF lasting for 7 days or less were randomized to receive an intravenous infusion of 3 or 5 mg kg^−1^ AP30663 or placebo. The trial was prematurely discontinued because of slow enrollment during the coronavirus disease 2019 pandemic. The primary endpoint of the trial was cardioversion from AF to sinus rhythm within 90 min from the start of the infusion, analyzed with Bayesian statistics. Among 59 patients randomized and included in the efficacy analyses, the primary endpoint occurred in 42% (5 of 12), 55% (12 of 22) and 0% (0 of 25) of patients treated with 3 mg kg^−1^ AP30663, 5 mg kg^−1^ AP30663 or placebo, respectively. Both doses demonstrated more than 99.9% probability of superiority over placebo, surpassing the prespecified 95% threshold. The mean time to cardioversion, a secondary endpoint, was 47 (s.d. = 23) and 41 (s.d. = 24) minutes for 3 mg kg^−1^ and 5 mg kg^−1^ AP30663, respectively. AP30663 caused a transient increase in the QTcF interval, with a maximum mean effect of 37.7 ms for the 5 mg kg^−1^ dose. For both dose groups, no ventricular arrhythmias occurred and adverse event rates were comparable to the placebo group. AP30663 demonstrated AF cardioversion efficacy in patients with recent-onset AF episodes. K_Ca_2 channel inhibition may be an attractive mechanism for rhythm control of AF that should be studied further in randomized trials. ClinicalTrials.gov registration: NCT04571385.

## Abstract (web summary)

In a phase 2 clinical trial, an inhibitor of the K_Ca_2 potassium channel, which conducts a repolarizing current in the heart, met the primary endpoint of promoting cardioversion from atrial fibrillation to sinus rhythm within 90 minutes of drug administration.

## Main

Atrial fibrillation (AF) is the most common cardiac arrhythmia and is associated with reduced quality of life and increased risk of stroke, heart failure and death^1^. Current treatment options for patients with AF episodes include pharmacological and electrical cardioversion as well as ‘wait and see’ approaches^1–3^. However, current pharmacological options for cardioversion have limited efficacy and a substantial risk of serious adverse effects, particularly an increased risk of causing potentially life-threatening ventricular arrhythmia, also known as proarrhythmia^1,2^. Consequently, use of these drugs are restricted in patients with left ventricular hypertrophy, ischemic heart disease and heart failure, resulting in barriers for prescribing the drugs and making many patients with AF ineligible for treatment. To avoid proarrhythmia, researchers have pursued drug targets with atrial but not ventricular effects, but have so far failed to demonstrate AF efficacy with these agents^4–6^.

**Extended Data Fig. 1:**
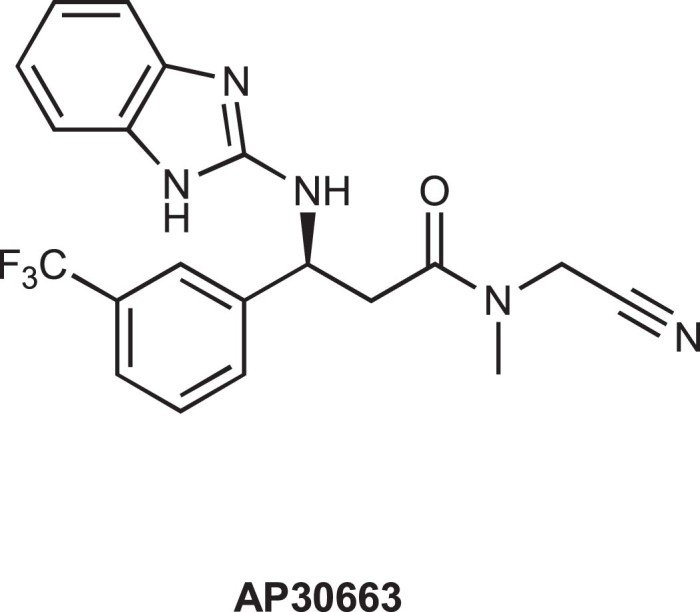
AP30663 chemical structure. AP30663 chemical name: (3 *R*)-3-[(1*H*-1,3-benzodiazol-2-yl)amino]-*N*-(cyanomethyl)-*N*-methyl-3-[3-(trifluoromethyl)phenyl]propenamide.

The K_Ca_2 ion (or SK) channel is a calcium-activated potassium channel that conducts a repolarizing current in the heart. It has the strongest association with AF in genome-wide association studies among genes encoding for ion channels^7^, with no association to the electrocardiogram (ECG) QT interval^8^. Inhibiting K_Ca_2 results in prolongation of the atrial action potential duration in tissue from humans with AF^9^; K_Ca_2 inhibitors have demonstrated efficacy in a range of animal models of AF without ventricular effects^10–13^. AP30663 is a new K_Ca_2 inhibitor with demonstrated efficacy in animals^14,15^; it was well tolerated in a phase 1 trial, although with a finding of transient QTc prolongation (see Extended Data Fig. 1 for its chemical structure)^16^.

The AF efficacy of K_Ca_2 inhibition in humans has not previously been tested. In this phase 2 trial, we studied the cardioversion efficacy of a single intravenous infusion of AP30663 in patients with a recent-onset AF episode.

## Results

### Patient characteristics

**Fig. 1:**
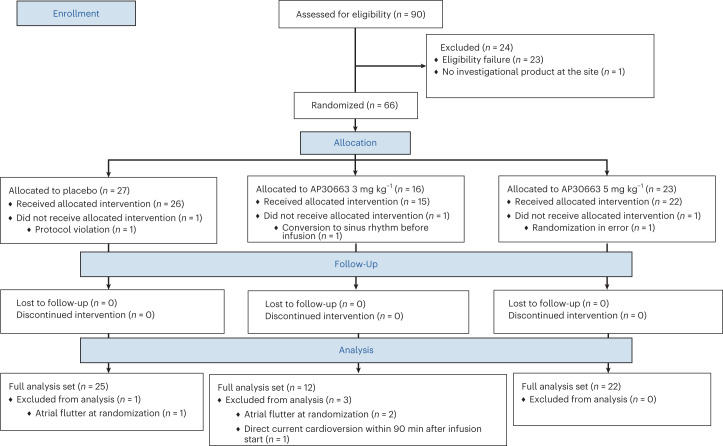
Patient flow diagram. Figure shows patient flow through the trial and reasons for exclusion. The full analysis set was used for all efficacy analyses including for the primary endpoint.

We enrolled patients with a current episode of AF lasting 7 days or less and randomized them to receive an intravenous infusion of 3 or 5 mg kg^−1^ AP30663 or placebo. The trial was prematurely discontinued in December 2022 because of slow enrollment under the coronavirus disease 2019 pandemic. A total of 66 patients were randomized between 24 September 2019 and 9 December 2022. Of these, three patients did not receive the infusion and a further four were excluded from the full analysis set due to having atrial flutter instead of AF at randomization (*n* = 3) or undergoing direct current cardioversion within the 90 min during which the primary endpoint was assessed (*n* = 1). No patients discontinued the infusion and all patients receiving the infusion completed the 30 days follow-up visit (Fig. 1). The mean age in the three groups was between 64.3 and 65.5, 68.2–80.0% were male, 13.6–40.0% had heart disease and the mean AF episode duration was 59.9–88.0 h across groups, with a shorter mean duration in the placebo group (59.9 h) compared to the active groups (3 mg kg^−1^: 88.0; 5 mg kg^−1^: 87.9 h) (Table 1). No other meaningful differences were observed in baseline characteristics across the three groups.

**Table 1:** Patient demographics and baseline characteristics in the safety analysis set

Characteristic	Placebo (*n* = 26)	AP30663 3 mg kg^−1^ (*n* = 15)	AP30663 5 mg kg^−1^ (*n* = 22)
Age, years	64.3 ± 9.23	65.4 ± 8.48	65.5 ± 10.38
Male sex, *n* (%)	18 (69.2)	12 (80.0)	15 (68.2)
Weight, kg	88.6 ± 14.6	90.3 ± 11.9	85.4 ± 12.0
Duration of current AF episode, h	59.9 ± 43.0	88.0 ± 24.6	87.9 ± 45.5
Prior diagnosis of AF, *n* (%)	6 (23.1)	5 (33.3)	4 (18.2)
Time since first AF diagnosis, years	4.3 ± 2.8	2.4 ± 2.7	6.7 ± 12.3
Heart rate during AF, bpm	98.0 ± 24.1	102.0 ± 28.7	93.9 ± 19.2
ECG QTcF interval, ms	408.0 ± 17.4	418.8 ± 27.2	409.9 ± 25.9
Left ventricular ejection fraction, %	60.3 ± 8.4	57.8 ± 9.7	57.3 ± 10.2
Left atrial dimension and diameter (anterior–posterior, end systolic), mm	47.5 ± 9.2	51.9 ± 9.5	45.2 ± 7.9
Heart disease, *n* (%)	6 (23.1)	6 (40.0)	3 (13.6)
Ischemic heart disease, *n* (%)	4 (15.4)	6 (40.0)	2 (9.1)
Heart failure, *n* (%)	1 (3.8)	0	2 (9.1)
Valvular heart disease, *n* (%)	1 (3.8)	0	0
Diabetes, *n* (%)	8 (30.8)	8 (53.3)	4 (18.2)
Oral anticoagulant drug use, *n* (%)	18 (69.2)	11 (73.3)	13 (59.1)
Rate control drug use, *n* (%)^a^	20 (76.9)	13 (86.7)	9 (40.9)
Betaxolol, *n* (mean daily dose)	0	1 (10 mg)	1 (40 mg)
Bisoprolol, *n* (mean daily dose)	13 (6 mg)	5 (8 mg)	6 (4 mg)
Carvedilol, *n* (mean daily dose)	2 (38 mg)	3 (83 mg)	0
Metoprolol, *n* (mean daily dose)	4 (213 mg)	3 (134 mg)	0
Nebivolol, *n* (mean daily dose)	1 (10 mg)	2 (5 mg)	1 (5 mg)
Digoxin, *n* (mean daily dose)	0	0	1 (500 µg)
Prior AF ablation, *n* (%)	1 (3.8)	1 (6.7)	1 (4.5)

^a^Defined as digoxin or beta-blocker use. There was no use of verapamil in the trial.

The ± values represent the mean ± s.d. unless otherwise indicated.

### Efficacy endpoints

**Fig. 2:**
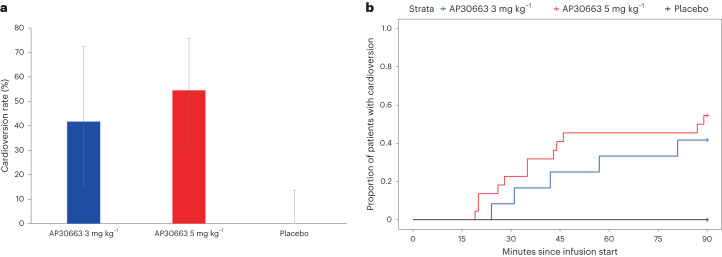
Primary endpoint of cardioversion. **a**, Cardioversion rates in the full analysis set. The bar heights show the percentage of patients with cardioversion; the error bars show the 95% confidence intervals (CIs). AP30663 3 mg kg^−1^, *n* = 12 patients; AP30663 5 mg kg^−1^, *n* = 22 patients; placebo, *n* = 25 patients. **b**, Time to cardioversion in the full analysis set.

The primary endpoint of cardioversion within 90 min occurred in 42% (5 of 12) and 55% (12 of 22) of patients receiving AP30663 at 3 mg kg^−1^ and 5 mg kg^−1^, respectively, and in 0% (0 of 25) of patients receiving placebo (Fig. 2 and Table 2). The posterior probability of superiority to placebo was greater than 99.9% for both doses, thereby exceeding the prespecified level of 95% and confirming superiority for both doses versus placebo. Secondary endpoints all numerically favored the active treatment over placebo: mean time to cardioversion was 47 min (s.d. ± 23) for AP30663 3 mg kg^−1^ and 41 min (s.d. ± 24) for AP30663 5 mg kg^−1^. Patients who did not convert within the 90 min after the start of the infusion underwent a direct current cardioversion; a post hoc analysis of this showed 100% success in cardioversion to sinus rhythm for both AP30663 dose groups versus 88% (22 of 25) for those receiving placebo. All patients treated with AP30663 3 mg kg^−1^ and 5 mg kg^−1^ were in sinus rhythm 24 h after the start of the infusion, compared with 76% (19 of 25) of patients receiving placebo. One relapse of AF within 5 min after pharmacological or direct current cardioversion was seen, occurring after a direct current cardioversion in the placebo group.

**Table 2:** Efficacy endpoints in the full analysis set

	Placebo (*n* = 25)	AP30663 3 mg kg^−1^ (*n* = 12)	AP30663 5 mg kg^−1^ (*n* = 22)
Primary endpoint			
Cardioversion within 90 min, *n* (%)	0/25 (0)	5/12 (42)	12/22 (55)
Within-arm 95% CI, %	0–14%	15–72%	32–76%
Posterior probability of superiority versus placebo, %	NA	>99.9%	>99.9%
Secondary endpoints			
Time to cardioversion, min ± s.d.	NA	47 ± 23	41 ± 24
*P* for pairwise comparison to placebo	NA	0.001	<0.0001
Relapse of AF within 5 min after pharmacological or direct current cardioversion, *n* (%)	1/25 (4.0)	0	0
Sinus rhythm 3 h after the start of the infusion, *n* (%)	21/25 (84.0)	11/11 (100)	20/21 (95.2)
Sinus rhythm 24 h after the start of the infusion, *n* (%)	19/25 (76)	11/11 (100)	21/21 (100)
Sinus rhythm 30 days after the start of the infusion, *n* (%)	16/25 (64.0)	9/10 (90.0)	15/21 (71.4)
Patients with direct current cardioversion, *n* (%)	25/25 (100)	7/12 (58)	10/22 (45)
Successful direct current cardioversion, *n* (%)^a^	22/25 (88)	7/7 (100%)	10/10 (100)

^a^Post hoc analysis.

The primary endpoint was analyzed with Bayesian statistics and the prior probability of success at a dose was modeled with a uniform Beta (1,1) prior. AP30663 was considered superior to placebo if the posterior probability was greater than 0.95. Time to cardioversion was analyzed using Kaplan–Meier curves and two-sided *P* values are reported for pairwise comparison to placebo. No adjustment for multiple testing was done. NA, not applicable.

None of the three individuals (one allocated to placebo and two allocated to AP30663 3 mg kg^−1^) who were excluded from the full analysis set because of having atrial flutter at randomization met the primary endpoint.

### Safety endpoints

Adverse events were reported in 50% (13 of 26) of patients for the AP30663 5 mg kg^−1^ group, 27% (4 of 15) for AP30663 3 mg kg^−1^ and 50% (11 of 22) for placebo (Table 3). No deaths occurred and all four serious adverse events were observed in the placebo group. All four serious adverse events were recurrence of AF that led to hospitalization. Changes in systolic blood pressure were 1.2 mmHg (s.d. = 10.1) for the AP30663 5 mg kg^−1^ group, 3.4 mmHg (s.d. = 10.3) for AP30663 3 mg kg^−1^ and 1.7 mmHg (s.d. = 11.7) for placebo, all measured during the infusion. Changes in diastolic blood pressure were 2.2 mmHg (s.d. = 8.3) for the AP30663 5 mg kg^−1^ group, −2.7 mmHg (s.d. = 7.6) for the AP30663 3 mg kg^−1^ group and −0.5 mmHg (s.d. = 7.1) for the placebo group, all measured during the infusion (Extended Data Table 1).

**Table 3:** Adverse events in the safety analysis set

Event	Placebo (*n* = 26)	AP30663 3 mg kg^−1^ (*n* = 15)	AP30663 5 mg kg^−1^ (*n* = 22)
Adverse event	13 (50.0) 19	4 (26.7) 6	11 (50.0) 19
Serious adverse event^a^	4 (15.4) 4	0	0
Leading to death	0	0	0
Adverse event leading to discontinuation of infusion	0	0	0
Adverse event according to organ class and preferred term			
Cardiac disorders	13 (50.0) 15	2 (13.3) 3	9 (40.9) 13
AF	11 (42.3) 12	1 (6.7) 1	7 (31.8) 7
Atrial flutter	1 (3.8) 1	0	3 (13.6) 3
Atrioventricular block (first-degree)	1 (3.8) 1	2 (13.3) 2	0
Left bundle branch block	0	0	1 (4.5) 1
Right bundle branch block	0	0	1 (4.5) 1
Left ventricular failure	0	0	1 (4.5) 1
Supraventricular tachycardia	1 (3.8) 1	0	0
Vascular disorders	2 (7.7) 2	2 (13.3) 2	1 (4.5) 1
Hypotension	0	1 (6.7) 1	1 (4.5) 1
Phlebitis	2 (7.7) 2	0	0
Hypertension	0	1 (6.7) 1	0
Investigations	0	1 (6.7) 1	1 (4.5) 1
Increased blood bilirubin	0	0	1 (4.5) 1
Electrocardiogram prolonged QT	0	1 (6.7) 1	0
Metabolism and nutrition disorders	1 (3.8) 1	0	1 (4.5) 1
Diabetes mellitus	0	0	1 (4.5) 1
Hypokalemia	1 (3.8) 1	0	0
Renal and urinary disorders	0	0	2 (9.1) 2
Hematuria	0	0	2 (9.1) 2
Nervous system disorders	0	0	1 (4.5) 1
Postural dizziness	0	0	1 (4.5) 1
Psychiatric disorders	1 (3.8) 1	0	0
Insomnia	1 (3.8) 1	0	0

All data are based on investigator-reported adverse events. Data reported are the number of patients (%) and number of events (*n*). ^a^A serious adverse event was defined as death, a life-threatening episode, hospitalization or prolongation of existing hospitalization, a persistent or substantial disability or incapacity, or an event otherwise considered to be an important medical event.

**Extended Data Fig. 2:**
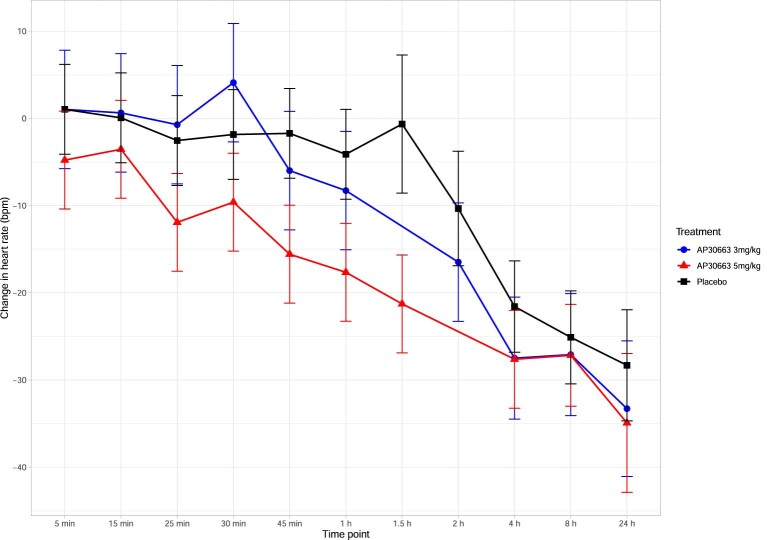
Changes in heart rate in the safety analysis set. The plots show least squares mean and 90% CI based on a linear mixed-effects model. AP30663 3 mg/kg n = 15 patients, AP30663 5 mg/kg n = 22 patients, Placebo n = 26 patients.

A mean decrease in heart rate was seen for all groups. This occurred earlier for the active groups compared to the placebo group, coinciding with the timing of conversions from AF to sinus rhythm (Extended Data Fig. 2).

**Fig. 3:**
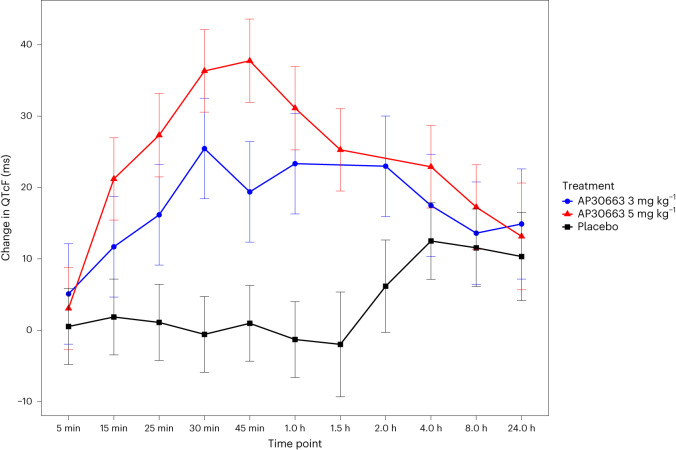
Change in QTcF from baseline in the safety analysis set. Plot showing the least squares mean and 90% CIs based on a linear mixed-effects model at the indicated time points. AP30663 3 mg kg^−1^, *n* = 15 patients; AP30663 5 mg kg^−1^, *n* = 22 patients; placebo, *n* = 26 patients.

A transient difference in change in QTcF was observed (Fig. 3), with an estimated maximum least squares mean effect of +37.7 ms (s.e.m. = 3.5) at 45 min for the AP30663 5 mg kg^−1^ group, compared to +19.4 ms (s.e.m. = 4.3) for the 3 mg kg^−1^ group and −1.3 ms (s.e.m. = 3.21) for the placebo group (Extended Data Table 2). QTcF changes for all groups, including the placebo group, remained at more than 10 ms at 24 h. No other meaningful differences in ECG markers were observed.

With Holter monitoring, the most important ventricular finding was episodes of nonsustained ventricular tachycardia seen in both the active and placebo groups with the longest episodes being four beats (Extended Data Table 3).

No apparent effect of AP30663 was observed on laboratory parameters (Extended Data Table 4).

### Pharmacokinetic endpoint

**Extended Data Fig. 3:**
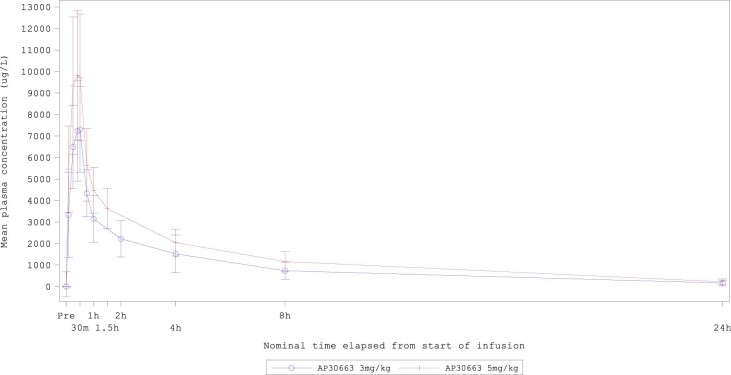
AP30663 mean plasma concentration in the pharmacokinetic analysis set. H: hours. Error bars show the standard deviation. AP30663 3 mg/kg n = 15 patients, AP30663 5 mg/kg n = 22 patients.

The plasma concentration over time is shown in Extended Data Fig. 3.

## Discussion

In this phase 2 clinical trial, AP30663, a new K_Ca_2 ion channel inhibitor, demonstrated efficacy in cardioverting AF to sinus rhythm compared to placebo in patients with a recent-onset AF episode. There was a numerical dose–response between the two doses tested and results from all secondary endpoints supported the efficacy of AP30663.

Among currently existing drugs for pharmacological AF cardioversion, vernakalant and flecainide are the most efficacious^1^. Most clinical trial data are available for vernakalant, which is a multichannel blocker that targets cardiac sodium channels and the atrium-specific potassium channel K_V_1.5, but not K_Ca_2 (refs. ^17,18^). Vernakalant has demonstrated superior efficacy to ibutilide and amiodarone in randomized trials^19,20^. In a meta-analysis that included placebo-controlled trials enrolling a very similar population to ours, vernakalant had a 48% cardioversion rate within 90 min versus 6% with placebo, resulting in a 42% placebo-adjusted cardioversion rate^21^. In our trial, AP30663, at the highest dose tested, demonstrated a 55% placebo-adjusted cardioversion rate, supporting a competitive efficacy of AP30663.

The clinical efficacy of AP30663 aligns with the effects observed in animal models, where AP30663 showed a pronounced effect on the atrial effective refractory period in one pig model, and successfully cardioverted AF and prevented its reinduction in another pig model where vernakalant did not cardiovert any longer^14^. Other K_Ca_2 inhibitors have shown similar AF antiarrhythmic effects in rats, guinea pigs, rabbits, pigs, dogs, goats and horses; however, none of these have been tested in humans^10–13,22^.

AP30663 inhibits the K_Ca_2 channel through negative allosteric modulation that decreases the calcium sensitivity of the channel. A recent study found that the K_Ca_2 current is upregulated and is the dominant repolarizing atrial current in patients with AF because of increased Ca^2+^ sensitivity and increased trafficking of K_Ca_2 to the cell membrane^9^. This upregulation of the K_Ca_2 current in patients with AF contributes to the action potential shortening characteristic seen in AF; the AP30663 mechanism of action can be hypothesized to directly counteract this effect.

The safety profile of AP30663 was consistent with results from the phase 1 trial, with the exception that infusion site reactions were reported in the phase 1 trial whereas none were reported in the current trial^16^. Inhibition of the K_Ca_2 channel in animal studies has been associated with central nervous system adverse effects in the form of tremors and ataxia at high doses; this was also seen with AP30663 in the safety animal studies^16^. However, these effects have not been observed with AP30663 in any of the clinical trials, including the current one. Although AP30663 caused a transient increase in the QTcF interval, no clinically relevant ventricular arrhythmias were observed in any treatment group. The increase in QTcF remained above 10 ms for all groups including the placebo group at 24 h, highlighting the difficulties in QTc assessment when comparing baseline values assessed while patients are in AF with post-baseline values assessed once patients converted to sinus rhythm, with resulting changes in heart rate. These findings suggest that the maximum increase in QTcF seen at 45 min is probably overestimated for the active groups because some patients had converted to sinus rhythm at this time point. In genome-wide association studies, no association between the genes encoding for the K_Ca_2 channels and the QTc interval has been found^8^. Additionally, while AP30663 potently inhibits K_Ca_2, it also inhibits the K_v_11.1 channel (KCNH2 or hERG gene) to a lesser degree^16^. K_v_11.1 inhibition is the most common cause of drug-related QTc prolongation^23^; collectively, these findings suggest that the QTc increase is caused by an off-target inhibition of the K_v_11.1 channel.

Existing drugs with different mechanisms of action, when effective in AF cardioversion, also show efficacy in sinus rhythm maintenance (prevention of AF recurrence)^1,2,24–26^. Our demonstration of human cardioversion efficacy through inhibition of K_Ca_2 suggests that this may also be a promising drug target for maintaining sinus rhythm.

This trial has some limitations. We enrolled patients with an AF episode lasting less than 7 days. Other drugs have shown decreased efficacy with longer AF durations^27–29^, and efficacy should not be generalized to patients with longer AF episodes. The trial had a limited sample size. The early termination of the trial because of slow enrollment, which prevented testing doses higher than 5 mg kg^−1^. Underrepresentation of female patients in the trial was another limitation. Collectively, the results of this phase 2 trial should be considered hypothesis-generating; the efficacy and safety, in particular the potential consequences of QT prolongation, need to be studied in a larger phase 3 trial.

In conclusion, the K_Ca_2 inhibitor AP30663 demonstrated superior AF cardioversion efficacy compared with placebo in patients with recent-onset AF episodes. AP30663 caused a transient increase of the QTc interval, but no ventricular arrhythmias were observed. K_Ca_2 inhibition may be an attractive pathway for rhythm control of AF and should be studied in future randomized trials.

## Methods

### Trial design

We conducted a phase 2 randomized, double-blind, placebo-controlled, parallel-group trial with an adaptive design with the potential to test doses between 2 and 6 mg kg^−1^. An independent data monitoring committee reviewed unblinded safety and efficacy data during the trial and was responsible for providing recommendations to the sponsor regarding dose changes according to the adaptive design (see the ‘Statistical analyses’ section of the Methods). See Supplementary Note 1 for a list of data monitoring committee members.

The maximum number of individuals that could be enrolled based on the adaptive design was 108. Patients wore 12-lead Holter monitors to assess both efficacy and safety for at least 8 h after the infusion; the collected data were analyzed by a core laboratory for arrhythmias and semiautomated measurement of ECG intervals based on triplicate ECG extracts at prespecified time points. Patients were followed until day 30 after receiving the infusion. Please refer to the protocol in Supplementary Note 2 for further information.

In December 2022, the sponsor decided to stop the trial early because of slow enrollment, mainly due to the coronavirus disease 2019 pandemic, at a time when the sample size in the 5 mg kg^−1^ dose allowed for sufficient statistical power to evaluate this dose. At that point in the adaptive design, subsequent interim analysis could have allowed testing of a 4 or 6 mg kg^−1^ dose.

The trial protocol was approved by the following ethics committees: Den Videnskabsetiske Komité, Region Sjælland, Denmark and Egészségügyi Tudományos Tanács Klinikai Farmakológiai Etikai Bizottsága, Budapest, Hungary.

All participants provided written informed consent. The trial was sponsored and funded by Acesion Pharma.

ClinicalTrials.gov registration: NCT04571385. EudraCT registration: 2018-004445-17.

### Trial population

Male and female patients aged 18–80 years with a body weight of 50–110 kg and a current episode of symptomatic AF lasting between 3 h and 7 days were deemed eligible. Patients were required to be treated with anticoagulation according to current guidelines. Patients were allowed to have stable ischemic heart disease and heart failure (New York Heart Association classes I and II, left ventricular ejection fraction 40% or higher). Exclusion criteria included recent cardioversion, current or recent use of antiarrhythmic drug classes I or III (including amiodarone), QTcF greater than 450 ms or previous torsade de pointes episodes. The complete list of inclusion and exclusion criteria can be found in the next sections.

### Inclusion criteria

The inclusion criteria were: provision of written informed consent; clinical indication for cardioversion of AF; current episode of symptomatic AF lasting between 3 h and 7 days inclusive at randomization; adequate anticoagulation according to international or national guidelines; body weight 50–110 kg inclusive (with clothes, without shoes); and male patients and postmenopausal women aged 18–80 years inclusive.

### Exclusion criteria

The exclusion criteria were: significant clinical illness or surgical procedure within 4 weeks before the screening visit; present renal dysfunction (estimated glomerular filtration rate less than 30 ml min^−1^), hepatic dysfunction (alanine aminotransferase or aspartate aminotransferase higher than three times the upper limit of normal) or uncontrolled hyperthyroidism or hypothyroidism; a history of significant mental, renal or hepatic disorder, chronic obstructive pulmonary disease or other significant disease, as judged by the investigator; any cardioversion attempt of AF or atrial flutter within 1 week before randomization. Previous failed attempt (no conversion) of pharmacological or direct current cardioversion of previous or current AF episode; failure to find a large antecubital (or equivalent) vein for the infusion; any of the following events, or any other significant cardiovascular event as judged by the investigator, during the last 6 weeks before randomization: myocardial infarction, unstable angina pectoris or other signs of myocardial ischemia, stroke or transient ischemic attack, myocardial revascularization (percutaneous coronary intervention, coronary artery bypass graft) or other revascularization procedure; hemodynamically unstable condition as judged by the investigator; systolic blood pressure lower than 90 mmHg or higher than 180 mmHg, or diastolic blood pressure higher than 105 mmHg at randomization; blood hemoglobin lower than 100 g l^−1^ at screening; congestive heart failure according to New York Heart Association class III or IV; left ventricular ejection fraction lower than 40% on echocardiography or other clinically significant abnormality on the echocardiogram (not older than 6 months), as judged by the investigator; known hypertrophic cardiomyopathy or significant left ventricular hypertrophy (free wall or septal thickness greater than 13 mm); any clinically significant valvular heart disease; a history or previous signs of sinus nodal disease; pacemaker or implantable cardioverter defibrillator therapy; a personal or family history of torsades de pointes, any other polymorphic ventricular tachycardia, sustained ventricular tachycardia, long QT syndrome or Brugada syndrome; QTc (QTcF) interval greater than 450 ms at randomization. When measured during AF, the mean heart rate should be 50–100 bpm. The QTcF should be calculated at AF as the mean of at least five consecutive RR intervals with consecutive QT intervals; QRS complex duration longer than 120 ms at randomization; known atrioventricular (AV) block I (prolonged PQ interval longer than 220 ms), AV block II, AV block III or complete bundle branch block; potassium in serum below 3.5 or above 5.3 mmol l^−1^ at randomization. Patients with low potassium levels at screening may be appropriately supplemented with potassium before baseline, according to local standards. Retesting of the potassium level is required and the patient can be randomized after potassium has returned to the reference range; anticipated change in dose or initiation of loop diuretic from screening to the end of the infusion; use of any antiarrhythmic drug class I or III within 7 days or, for amiodarone specifically, 12 weeks before randomization; use of QT-prolonging drug or drug that inhibits cytochrome P450 3A4, as well as St John’s wort within 10 days before randomization; administration of an investigational drug within the preceding 3 months before randomization; administration of AP30663 at any time before randomization; a history of drug addiction or alcohol abuse within the last 12 months, at the discretion of the investigator; blood or plasma donation within the preceding 4 weeks before randomization; any suspected or manifested clinically significant infection, as judged by the investigator; involvement in the planning and conduct of the study (applies to Acesion Pharma staff, Syneos Health staff and staff at the investigational site); clinical judgment by the investigator that the patient should not participate in the study; any malignant cancer within 3 years (except for successfully treated in situ nonmelanoma skin cancer and in situ cervical cancer) of signing the informed consent form.

The trial was conducted at ten sites in Hungary and Denmark.

### Trial intervention

The trial ended up testing 3 mg kg^−1^ and 5 mg kg^−1^ doses of AP30663 versus placebo; participants were randomized to AP30663 or placebo in a 1:1 ratio for part 1 of the trial testing the 3 mg kg^−1^ dose and subsequently in a 2:1 ratio. Randomization was performed using a computer-generated random sequence and interactive voice and Web response system. The intravenous infusion was prepared by an unblinded team and handed over to a blinded team for administration over 30 min. Use of other antiarrhythmic class I or III drugs (including amiodarone) was prohibited until the day after receiving the infusion.

If patients did not cardiovert within the 90 min after the start of the infusion, they were to undergo an electrical (direct current) cardioversion.

### Trial endpoints

The primary endpoint was the proportion of patients converting from AF to sinus rhythm within 90 min from the start of the infusion and who subsequently had no AF recurrence within 1 min of conversion. Key secondary efficacy endpoints included time to conversion from AF, the proportion of patients with relapse of AF within 5 min after pharmacological (AP30663) or direct current cardioversion and the proportion of patients in sinus rhythm at 3 and 24 h after the start of the infusion. Key secondary safety endpoints included adverse events and change in QT interval corrected using the Fridericia formula (QTcF). Pharmacokinetics were also assessed as a secondary endpoint.

Exploratory endpoints addressing the potential for treatment effect interactions with baseline variables and pharmacokinetic parameters were specified in the protocol; however, due to the limited sample size, these analyses were severely underpowered and are not reported in this article.

### Statistical analyses

To ensure an efficient adaptive trial design, Bayesian statistics were used. In part 1 of the trial, a dose of 3 mg kg^−1^ was tested; if deemed safe, a dose of 5 mg kg^−1^ as well as potentially a dose of 2 mg kg^−1^ were to be tested based on the results from an interim analysis. Dose testing according to the adaptive trial design was guided by achieving an AF cardioversion rate greater than 0.65 at the interim analysis; if the posterior probability of this was 0.90 or greater, the current dose would be closed for sufficient efficacy and a dose one level below would be opened, if available. If a posterior probability of this was less than 0.10, the current dose would be closed and a higher dose opened, if available. Interim analyses were done after enrolling 32 individuals in the 3 mg kg^−1^ dose, which led to further testing of the 5 mg kg^−1^ dose only. An interim analysis after enrolling 18 individuals in the 5 mg kg^−1^ dose did not result in dose change. The prior probability of success at a dose was modeled with a uniform Beta (1,1) prior; the posterior distribution was modeled for each dose independently using a Beta posterior distribution. This method was also applied to the final analysis of the primary endpoint because the primary analysis and each AP30663 dose were considered superior to placebo if the posterior probability was greater than 0.95. Time to conversion was analyzed using Kaplan–Meier curves. No type I error rate control was implemented across the interim analyses or at the final analysis because this was a phase 2 non-confirmatory trial. In all analyses, individuals given placebo were pooled.

ECG parameters were analyzed based on a linear mixed-effects model; a least squares mean with 90% CIs was reported for these.

The safety analysis set was prespecified to be used for all safety analyses and consisted of all randomized participants who were administered double-blind trial treatment. The full analysis set was prespecified to be the primary one for all efficacy analyses and consisted of all randomized participants who were administered double-blind trial treatment and had an evaluable AF conversion status within 90 min from the start of the infusion.

The sample size was selected based on a similar previous trial^30^; no formal sample size calculations were made. Please refer to the Statistical Analysis Plan in Supplementary Note 3 for further information.

Data analyses were done by the contract research organization Syneos Health and by the sponsor. All authors had access to the data. SAS v.9.4 or higher and R v.3.5.2 or higher were used for the analyses. Data were collected through the electronic data capture system Medidata Classic Rave (2019–2023).

### Reporting summary

Further information on research design is available in the Nature Portfolio Reporting Summary linked to this article.

## Online content

Any methods, additional references, Nature Portfolio reporting summaries, source data, extended data, supplementary information, acknowledgements, peer review information; details of author contributions and competing interests; and statements of data and code availability are available at 10.1038/s41591-023-02679-9.

### Supplementary information

Supplementary Information Supplementary Notes 1–3. Supplementary Note 1 contains the list of investigators and DMC members. Supplementary Note 2 contains the protocol. Supplementary Note 3 contains the SAP.

Reporting Summary

## Figures and Tables

**Extended Data Table 1:**
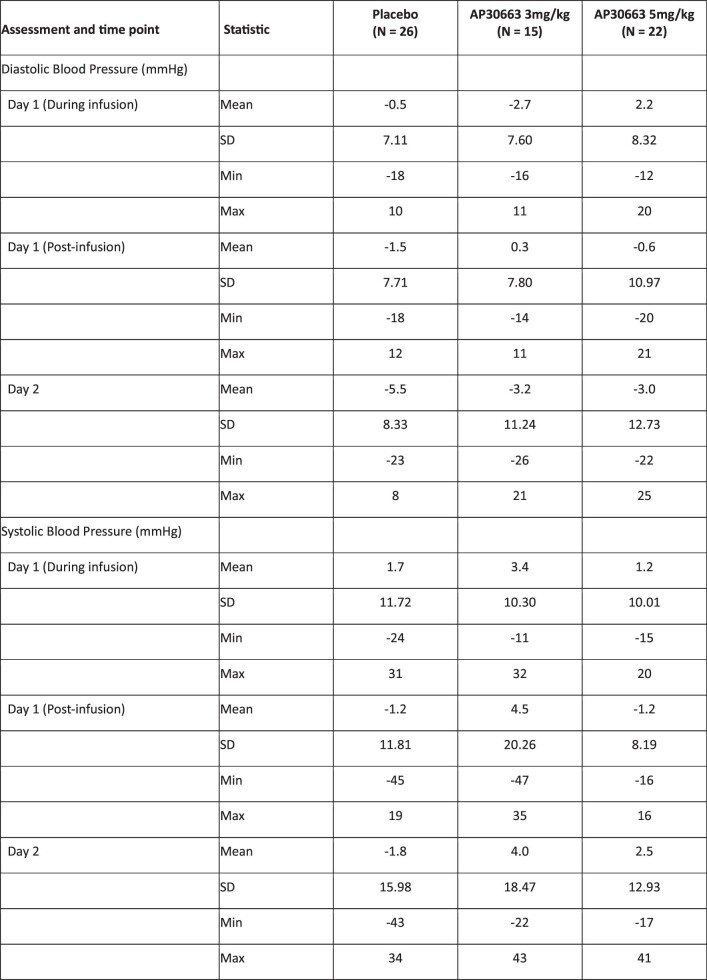
Changes in blood pressure in the safety analysis set

**Extended Data Table 2:**
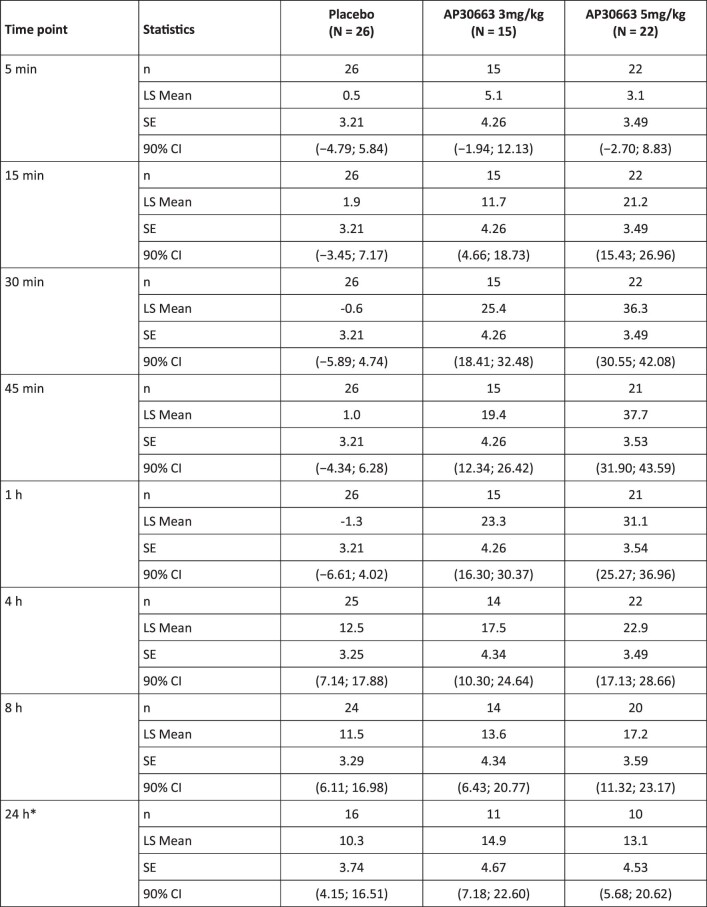
Changes in ECG QTcF interval in the safety analysis set

*In protocol version 4.0, Holter monitoring was shortened from 24 to 8 hours post-infusion.

Based on a linear mixed-effects model

**Extended Data Table 3:**
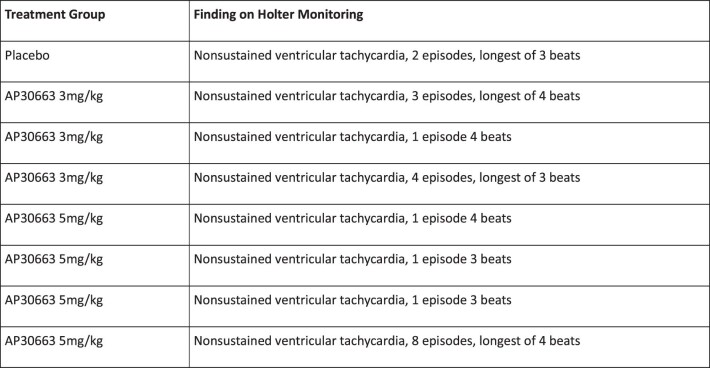
Ventricular tachyarrhythmia on Holter in the safety analysis set

**Extended Data Table 4:**
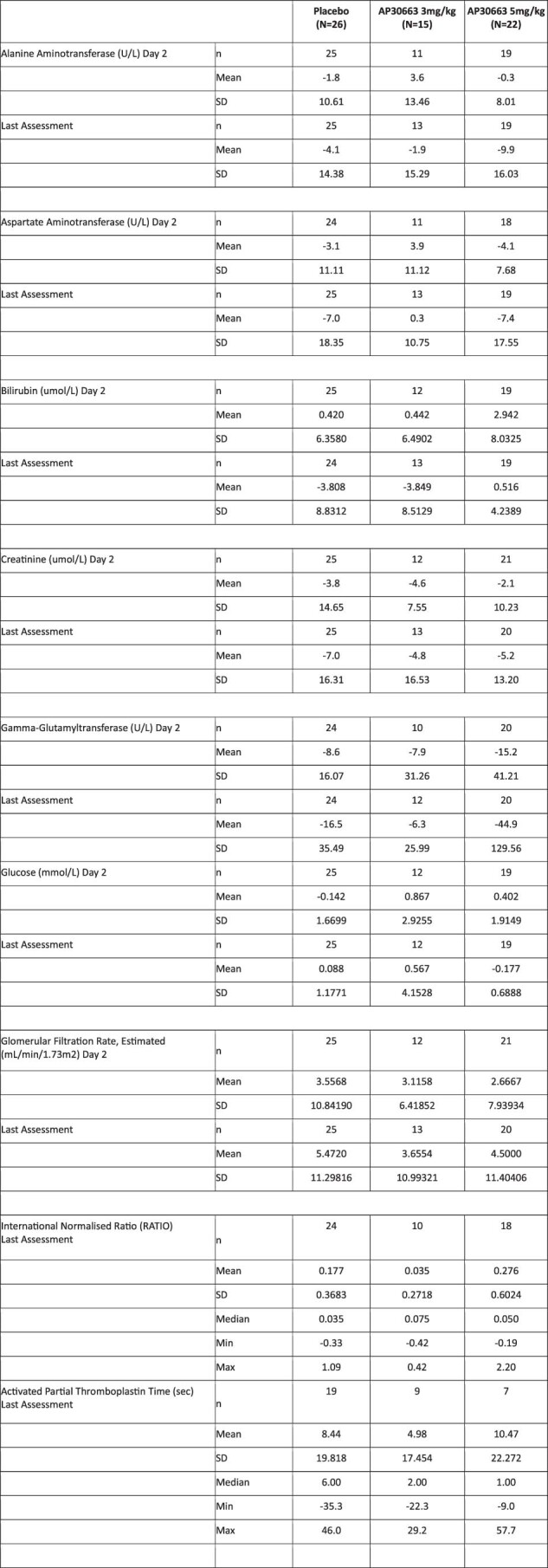
Changes in biochemistry parameters in the safety analysis set

Last Assessment is Day 30 or early discontinuation visit.

## Data Availability

Data originating from the trial are considered commercially sensitive; as such, they are not publicly available. To the extent that current legislation allows it, the authors will provide access to individual deidentified participant-level data that underlie the data presented in this article to researchers who provide a methodologically sound proposal for academic purposes to interpret, verify and extend research in the article that does not violate intellectual property or confidentiality obligations, beginning 12 months after article publication. Researchers should contact the corresponding author when applying for data access. Use of data will be restricted to the agreed purpose.
