# Supplementary material for: Inhibition of the KCa2 potassium channel in atrial fibrillation: a randomized phase 2 trial
Source: Nat Med. 2023 Dec 13;30(1):106–11. doi: 10.1038/s41591-023-02679-9 (PMC10803288; doi:10.1038/s41591-023-02679-9)
Supplement: Supplementary file 1 — Supplementary Notes 1–3. Supplementary Note 1 contains the list of investigators and DMC members. Supplementary Note 2 contains the protocol. Supplementary Note 3 contains the SAP. [file 41591_2023_2679_MOESM1_ESM.pdf]

nature medicine

Article https://doi.org/10.1038/s41591-023-02679-9

Inhibition of the $K_{Ca}2$ potassium channel in atrial fibrillation: a randomized phase 2 trial

In the format provided by the authors and unedited

Supplementary Note 1

List of investigators

Hungary: J. Tomcsányi, Budai Irgalmasrendi Kórház, Budapest; B. Benczúr, Balassa János Megyei Kórház, Szekszárd; J. Lippai, Jahn Ferenc Dél-pesti Kórház, Budapest; G. Lupkovics, Zala Megyei Szent Rafael Kórház, Zalaegerszeg; A. Vértes, DPC Kórház- Egyesitett Szent István es Szent László Kórhaz-Rendelőintézet, Budapest; L. Nagy, Csongrád Megyei Dr Bugyi Istvan Kórház, Szentes; K. Tóth, Pécsi Tudományegyetem Klinikai Központ I.sz. Belgyógyászati Klinika, Pecs.

Denmark: M. Lock Hansen, Gentofte University Hospital, Gentofte; T. Nielsen, Roskilde Sygehus, Roskilde; R. Sam, Aalborg University Hospital, Aalborg

Data Monitoring Committee

Stefan H. Hohnloser, MD, FESC, FHRS; Deepak L. Bhatt, MD, MPH; Paul Dorian, MD, M.Sc.

CLINICAL STUDY PROTOCOL

A Double-Blind, Randomised, Placebo-Controlled, Parallel-Group Study of AP30663 Given Intravenously for Cardioversion in Patients with Atrial Fibrillation

**Protocol Number:**	AP30663 – 2001
**IND Number:**	Not applicable
**EudraCT Number:**	2018-004445-17
**Syneos Health Study Number:**	7000776
**Investigational Product:**	AP30663
**Phase:**	Phase 2a
**Sponsor:**	Acesion Pharma ApS<br>Ole Maaløes Vej 3<br>DK-2200 Copenhagen N<br>Denmark
**Contract Research Organisation:**	Syneos Health<br>1030 Sync Street<br>Morrisville, NC 27560<br>United States
**Protocol Date:**	23 Mar 2022
**Protocol Version:**	Version 4.0, Final (DK and HU)<br>Version 3.1, Final (DK)<br>Version 3.0, Final (HU)<br>Version 2.0, Final (HU)<br>Version 1.1, Final (HU)

CONFIDENTIAL
This protocol may not be reproduced or communicated to a third party without the written permission of Acesion Pharma ApS.

1 PROTOCOL APPROVAL SIGNATURES

Protocol Title: A Double-Blind, Randomised, Placebo-Controlled, Parallel-Group Study of AP30663 Given Intravenously for Cardioversion in Patients with Atrial Fibrillation

Protocol Number: AP30663 – 2001

This study will be conducted in compliance with the clinical study protocol (and amendments), International Council for Harmonisation (ICH) guidelines for current Good Clinical Practice and applicable regulatory requirements.

Sponsor Signatory

[redacted] [redacted]

Signature

Date

[redacted]

Chief Medical Officer
Acesion Pharma ApS
Ole Maaløes Vej 3
DK-2200 Copenhagen N
Denmark

Sponsor Signatory

[redacted]

[redacted]

Signature

Director Clinical Operations
Acesion Pharma ApS
Ole Maaløes Vej 3
DK-2200 Copenhagen N
Denmark

Date

Contract Research Organisation (CRO) Signatory

Medical Monitor

[redacted]

Signature

Date

[redacted], Medical
Management and Scientific Services
Syneos Health
Prima Court Nowogrodzka 68
02-014 Warsaw
Poland

Contract Research Organisation (CRO) Signatory

Biostatistician

[redacted]

[redacted]
Syneos Health
1 Pinehurst Road
Farnborough, Hampshire
GU14 7BF
UK

Signature

Date

2 STUDY PERSONNEL

Sponsor Personnel

Name: [redacted]
Title: Chief Medical Officer
Address: Acesion Pharma ApS, Ole Maaløes Vej 3, DK-2200 Copenhagen N, Denmark
Telephone No.: [redacted]

Name: [redacted]
Title: Director Clinical Operations
Address: Acesion Pharma ApS, Ole Maaløes Vej 3, DK-2200 Copenhagen N, Denmark
Telephone No.: [redacted]

CRO Personnel

Medical Monitor

Name: [redacted]
Title: Senior Medical Director, Medical Management and Scientific Services
Address: [redacted]
Telephone No.: [redacted]

Project Manager

Name: [redacted]
Title: Sr Director, Project Delivery
Address: [redacted]
[redacted] [redacted]

ECG Laboratory

Company Name: Clario

Address: Lynchwood House
Peterborough Business Park
Peterborough PE2 6FZ
United Kingdom

Telephone No.: [redacted]

Clinical Laboratory

Company Name: Eurofins Central Laboratory, B.V.

Address: Bergschot 71
4817 PA
Breda
The Netherlands

Telephone No.: [redacted]

3 SYNOPSIS

Protocol Number:

AP30663 - 2001

Title:

A Double-Blind, Randomised, Placebo-Controlled, Parallel-Group Study of AP30663 Given Intravenously for Cardioversion in Patients with Atrial Fibrillation.

Investigational Product:

AP30663

Study Centres:

Approximately 15 centres in EU.

Phase:

2a

Objectives:

Primary objective:

- To demonstrate the efficacy of 1 or more dose levels of AP30663 on the basis of the ability to convert atrial fibrillation (AF) after intravenous administration.

Secondary objectives:

- To study the stability of rhythm control (immediate relapse of AF [IRAF], i.e. within 5 min after conversion from AF).
- To study the importance of AF duration with respect to the efficacy and safety of 1 or more dose levels of AP30663.
- To evaluate the safety and tolerability of 1 or more dose levels of AP30663.
- To study the relationship between systemic exposure and response, with special regard to the conversion from AF and the effect on QRS and QTcF.

Exploratory objectives:

- To study demographic and echocardiographic variables, concomitant diseases, and concurrent medication with respect to the efficacy and safety of 1 or more dose levels of AP30663.
- To evaluate the pharmacokinetics (PK) of AP30663, including influence of dose, concomitant medication, concurrent diseases, and demographic variables.
- To explore the proportions of patients on AP30663 converting from AF and of patients randomised to placebo and converting at direct-current (DC) cardioversion.

Study Design:

This is a double-blind, randomised, placebo-controlled, parallel-group Phase 2a study of 1 or more doses of AP30663 for cardioversion in adult patients with AF. The study will be conducted in 2 parts (Part 1 and 2): Part 1 of the study is a fixed randomisation, placebo-controlled, parallel design, and Part 2 will be an adaptive design conducted with 1 or more further doses of AP30663 versus placebo. The study is designed to identify the dose of AP30663 to be evaluated further in later-phase research. Though the study investigates multiple doses of AP30663 versus placebo, the participating patients will be randomised to receive one dose of AP30663 (i.e. patients will not receive multiple dose levels).

In Part 1 of the study, up to 36 patients will be randomised on Day 1 in a fixed 1:1 ratio to receive AP30663 at 3 mg/kg or matching placebo as a single 30-minute intravenous infusion. Their participation will last until the

completion of their follow-up visit at Day 30. An interim analysis including efficacy, PK/PD, safety and tolerability data analyses will be conducted once the randomised patients have completed Day 2 assessments or terminated the study, to determine the starting dose(s) for Part 2 of the study. An independent data monitoring committee (DMC) will be convened to review the accumulating unblinded efficacy (including ECG and Holter ECG), PK/pharmacodynamics (PD), safety and tolerability data for the study collected up to the time of the interim analysis (IA).

Part 2 of the study is an adaptive design, conducted in a minimum of 18 and a maximum of 72 patients with 1 or more doses of AP30663 (Dose [d] where d = 2, 4, 5, or 6 mg/kg) or placebo (d = 0). The maximum dose of 6 mg/kg AP30663 should not be exceeded under any circumstances, and the doses assessed during Part 2 will depend upon the cumulative results achieved across both Part 1 and Part 2. Based on the results seen at the end of Part 1 IAs and the decision made by the DMC regarding efficacy, safety and tolerability, the 3 mg/kg dose arm of AP30663 will be stopped, a 5 mg/kg dose arm of AP30663 will be opened and if high efficacy is seen (AF conversion rate > 0.65) then a 2 mg/kg AP30663 arm will also be opened. During Part 2, patients will be randomised in a ratio of 2:1 cumulatively in cohorts of 18 patients to a number of pre-defined doses of AP30663 versus placebo. After each cohort of 18 randomised patients has completed Day 2 assessments or terminated the study, an IA will be conducted to assess the AF conversion rate and subsequent dose arms to be assessed, with the following rules applied:

- If an open Dose d has at least 10 randomised patients who have completed Day 2 assessments, and satisfies: (Pd > 0.65) ≥ 0.90 then Dose d will be closed for sufficient efficacy response.
    - If the dose one level below this dose has never been opened, then dose d-1 is opened.
- If an open Dose d has at least 10 randomised patients who have completed Day 2 assessments, and satisfies (Pd > 0.65) < 0.10 then Dose d will be closed.
    - If Dose d was the largest dose thus far open, and Dose d is d < 6, then dose d+1 will be opened.

During Part 2, IAs will occur when 18, 36, and 54 patients have been randomised to open-doses including placebo, and have completed Day 2 assessments or terminated the study.

For Part 2, the DMC will give their recommendations regarding continuation or early termination of the study. Decision rules will be based on the AF conversion rate; however the DMC will also have the option to stop a dose arm (or the study) due to unacceptable safety and tolerability (including but not limited to increase in QTcF interval and number and severity of local injection site reactions), to be reviewed at fixed time points (see DMC Charter). Provided the study is not stopped at the end of Part 1 IA, Part 2 will include a minimum of 18 and a maximum of 72 additional patients.

<u>Data monitoring committee</u>

An independent DMC will be convened to review the unblinded data from the IA and to provide recommendations for Part 2 (see above). The DMC will also review accumulating efficacy, PK/PD, safety and tolerability data obtained at a subsequent IA in Part 2 of the study at defined time points. The safety and tolerability signal includes but not limited to increase in QTcF internal and number and severity of local injection site reactions.

Number of Patients:

Approximately 41, 62, 82, 103, or 123 patients will need to be screened to achieve up to 36, 54, 72, 90, or 108 randomised patients, respectively, who have completed Day 2 assessments or terminated the study, depending on the study design and recommendations of the DMC at each of the IAs. The number of patients screened is based on a screen failure rate of 12%.

Treatment:

In Part 1, study treatment will either be AP30663 3 mg/kg or matching placebo, randomised in a 1:1 ratio; both will be administered as an intravenous infusion for 30 min on Day 1. The DMC may either recommend to stop the study at the end of Part 1 IA, or may recommend to increase the dose of AP30663 to 4, 5, or 6 mg/kg, or

decrease to 2 mg/kg, alongside placebo during Part 2 of the study, dependent on the pre-defined decision criteria for efficacy and/or safety and tolerability review.

Study Duration:

Study drug infusion is planned for 30 min for each patient.

The overall study duration for each patient is up to a maximum of 42 days. The screening and randomisation will occur within 7 days (inclusive) from onset of AF. The duration of treatment and post-treatment follow-up will be 30 days ± 5 days from start of infusion to the follow-up visit.

Study Population:

Inclusion criteria:

1. Provision of written informed consent.
2. Clinical indication for cardioversion of atrial fibrillation.
3. Current episode of symptomatic atrial fibrillation lasting between 3 h and 7 days inclusive at randomisation.
4. Adequate anticoagulation according to international and/or national guidelines.
5. Body weight 50 to 110 kg inclusive (with clothes, without shoes).
6. Male patients and postmenopausal women aged 18 to 80 years inclusive.
    - Male patients who are sexually active must agree to abstain from sexual activity or be willing to use a double-barrier method of birth control (i.e. any double combination of male or female condom with spermicidal gel, diaphragm, sponge or cervical cap with spermicidal gel) if they become sexually active from the time of consent and for 90 days after the infusion day.
    - Post-menopausal women are defined as being >12 months after last menstrual period.
    - Women can also be included if permanently sterilised since ≥6 weeks (i.e. documented hysterectomy, bilateral salpingectomy, bilateral oophorectomy). Breastfeeding women are excluded.

Exclusion criteria:

1. Significant clinical illness or surgical procedure within 4 weeks preceding the screening visit.
2. Present renal dysfunction (estimated glomerular filtration rate [eGFR] <30 mL/min), hepatic dysfunction (alanine aminotransferase [ALT] or aspartate aminotransferase [AST] >3 × upper limit of normal), or uncontrolled hyperthyroidism or hypothyroidism.
3. History of significant mental, renal or hepatic disorder, chronic obstructive pulmonary disease or other significant disease, as judged by the investigator.
4. Any cardioversion attempt of AF or atrial flutter within 1 week preceding randomisation.
5. Prior failed attempt (no conversion) of pharmacological or DC cardioversion of previous or current AF episode.
6. Failure to find a large antecubital (or equivalent) vein for the infusion.
7. Any of the following events, or any other significant cardiovascular event as judged by the investigator, during the last 6 weeks before randomisation: myocardial infarction, unstable angina pectoris or other signs of myocardial ischaemia, stroke or transient ischaemic attack, myocardial revascularisation (percutaneous coronary intervention [PCI], coronary artery bypass graft [CABG]), or other revascularisation procedure.
8. Haemodynamically unstable condition as judged by the investigator; systolic blood pressure (BP) <90 mm Hg or >180 mm Hg, or diastolic BP >105 mm Hg at randomisation.
9. Blood haemoglobin <100 g/L at screening.
10. Congestive heart failure New York Heart Association class III or IV. Left ventricular ejection fraction <40% on echocardiography, or other clinically significant abnormality on the echocardiogram (not older than 6 months) as judged by the investigator.
11. Known hypertrophic cardiomyopathy or significant left ventricular hypertrophy (free wall or septal thickness >13 mm).

12. Any clinically significant valvular heart disease.
13. History or previous signs of sinus nodal disease.
14. Pacemaker or implantable cardioverter defibrillator therapy.
15. Personal or family history of Torsades de Pointes, any other polymorphic ventricular tachycardia, sustained ventricular tachycardia, long QT syndrome, and/or Brugada syndrome.
16. QTc (Fridericia, QTcF) interval >450 ms at randomisation. (When measured during AF, the mean heart rate should be 50 to 100 bpm. The QTcF should be calculated at AF as the mean of at least 5 consecutive RR intervals with consecutive QT intervals).
17. QRS duration >120 ms at randomisation.
18. Known atrioventricular (AV)-block I (prolonged PQ [PR] interval >220 ms), AV-block II, AV-block III, or complete bundle branch block (BBB).
19. Potassium in serum below 3.5 or above 5.3 mmol/L at randomisation. Patients with low potassium levels at screening may be appropriately supplemented with potassium before baseline, according to the local standards. A re-test of the potassium level is required, and the patient can be randomised after the potassium has returned to reference range.
20. Anticipated change in dose or initiation of loop diuretic from screening to the end of infusion.
21. Use of any antiarrhythmic drug class I and/or III within 7 days or, for amiodarone specifically, 12 weeks before randomisation.
22. Use of QT-prolonging drug, and/or drug that inhibits cytochrome P450 (CYP)3A4, as well as St John's Wort within 10 days before randomisation.
23. Administration of an investigational drug within the preceding 3 months before randomisation.
24. Administration of AP30663 at any time before randomisation.
25. History of drug addiction and/or alcohol abuse within the last 12 months at the discretion of the investigator.
26. Blood or plasma donation within the preceding 4 weeks before randomisation.
27. Any suspected or manifested clinically significant infection as judged by the investigator.
28. Involvement in the planning and conduct of the study (applies to Acesion Pharma staff, Syneos Health staff, and staff at the investigational site).
29. Clinical judgement by the investigator that the patient should not participate in the study.
30. Any malignant cancer within 3 years (except for successfully treated in-situ non-melanoma skin cancer and in-situ cervical cancer) of signing the informed consent form (ICF).

Primary Endpoint:

- The proportion of patients that have converted from AF within 90 min from the start of infusion and subsequently have no AF recurrence within 1 min of conversion from AF.

Secondary Endpoints:

Efficacy

- The time to conversion from AF from start of infusion.
- The proportion of patients with relapse of AF within 5 min (IRAF) after pharmacological or DC cardioversion.
- The proportion of patients in SR at 3 h ± 1 h after start of infusion.
- The proportion of patients in SR at 24 h ± 2 h after start of infusion.
- The proportion of patients in SR at 30 days ± 5 days after start of infusion.

Safety

- Adverse events (AEs), electrocardiogram (ECG) variables including significant arrhythmia, physical examination, vital signs, and laboratory evaluations.

- Changes in QTcF interval data over time.

Pharmacokinetics

- Systemic exposure derived from the population PK model.
- Population PK model parameter estimates derived from plasma concentrations of AP30663.

Efficacy:

Efficacy assessments include conversion from AF, which will be determined by the investigator. Holter will be used to record ECG continuously, to detect conversion from AF as well as to monitor sustainability of the rhythm after conversion.

Pharmacokinetics:

Venous blood sampling will be performed to determine plasma concentrations of AP30663 pre- and post-infusion at each time point.

Safety:

Safety assessments include review of AEs, clinical laboratory evaluations, vital signs (BP, heart rate and heart rhythm from ECG), physical examination, ECG and concomitant medications.

Laboratory assessments include haematology (haemoglobin, haematocrit, red blood cells [erythrocytes], white blood cells [leukocytes, total and differential], basophils, eosinophils, lymphocytes, monocytes, neutrophils, and platelets); clinical chemistry (alanine aminotransferase [ALT], aspartate aminotransferase [AST], bilirubin [direct, indirect, total], creatinine, gamma-glutamyltransferase, glucose [random], estimated glomerular filtration rate [eGFR], urea [blood urea nitrogen, BUN], magnesium, sodium, calcium, and potassium). Other laboratory variables determined are international normalised ratio (INR)/activated partial thromboplastin time (APTT) and thyroid stimulating hormone (TSH).

ECG used for safety assessments comprises 12-lead digital ECG, telemetry, and Holter.

Statistical Analysis:

Continuous data will be presented using descriptive summaries (e.g. mean, standard deviation, minimum, maximum, median, lower quartile, and upper quartile). Categorical data will be presented using the number of observations and relative (%) frequency.

Unless otherwise stated, the baseline value for any parameter will be the latest non-missing value taken prior to the infusion of study treatment on Day 1. Pre-infusion Day 1 assessments will be included in this derivation of baseline.

For the analysis of the primary endpoint (proportion of patients who have cardioversion within 90 min from the start of infusion) a Bayesian model will be utilised. The prior probability of success at a dose, d, is modelled with a Beta prior (uniform) for each dose. The posterior distribution is calculated for each dose independently using a Beta posterior distribution, $P_d \sim \text{Beta}(1 + X_d, 1 + N_d - X_d)$.

This modelling will occur for each interim analysis, and at the final analysis as primary method. At the conclusion of the study, each AP30663 dose will be considered to be superior to placebo, if the posterior probability of AP30663 dose has a higher success rate than placebo is greater than 0.95.

Further to this, for the final analysis, the primary endpoint will also be analysed by means of a logistic regression model. Relevant covariates at baseline may be added into the model after reviewing baseline imbalances.

Secondary endpoints relating to proportions will be analysed using a logistic regression model in a similar manner to that of the primary endpoint. Time to conversion will be analysed using Kaplan-Meier method and, if met, the median time to conversion will be presented, along with the corresponding Kaplan-Meier plot stratified by treatment group.

Interim analysis

The study is conducted in 2 parts (Part 1 and 2). For all interim analyses (IAs) during the study, a separate unblinded biostatistical team will prepare and present the unblinded analysis, and the DMC will be convened to review the data and will give their recommendations pertaining to continuation or early termination of the study, and as well as whether or not to increase/decrease the dose per the algorithm below and/or any other available data.

The first IA will be conducted once all randomised patients have completed Day 2 assessments or terminated the study. The DMC will be convened to review unblinded data on the primary efficacy endpoint, PK/PD, safety and tolerability data during this IA.

If the DMC decides that the study should continue, then Part 2 of the study will be enacted. During Part 2, IAs will be conducted when 18, 36, and 54 randomised have completed Day 2 assessments or terminated the study.

For each IA, the success rate for any single dose level d, *P*d, will be analysed using a Bayesian model, and the decision criteria as stated above will be implemented. The study will continue until all doses of AP30663 have been closed or the maximum of 72 patients during Part 2 (up to 108 patients total across Parts 1 and 2) have been enrolled.

Interim Analysis at End of Part 1:

During the first IA the following will occur, regardless of the response seen for the AP30663 3 mg/kg dose:

- Dose 3 (3 mg/kg AP30663) will be stopped.
- Dose 5 (5 mg/kg AP30663 versus placebo) will be opened.

Additionally, the following decision will be made dependent on the response rate seen for 3 mg/kg AP30663:

- If the proportion of patients achieving a response under AP30663 3 mg/kg is > 0.65 then Dose 2 (2 mg/kg AP30663) will additionally be opened for Part 2 (in parallel to Dose 5).

Interim Analyses during Part 2:

Interim analyses will occur when 18, 36, and 54 randomised patients have completed Day 2 assessments or terminated the study in Part 2. At these IAs the following rules will be utilised:

- If an open Dose d has at least 10 randomised patients having completed Day 2 assessments and satisfies: (*P*d > 0.65) ≥ 0.90 then Dose d will be closed for sufficient efficacy response.
    - If the dose one level below this dose has never been opened, then dose d-1 is opened.
- If an open Dose d has at least 10 randomised patients having completed Day 2 assessments and satisfies (*P*d > 0.65) < 0.10 then Dose d will be closed.
    - If Dose d was the largest dose thus far open, and Dose d is d < 6, then dose d+1 will be opened.

The study will continue until all doses of AP30663 have been closed or the maximum of 72 patients during Part 2 (up to 108 patients total across Part 1 and Part 2) have been enrolled.

While the algorithm, and the DMC's decision for dose increase/decrease, is based on AF conversion rate only, the DMC will also convene to review safety and tolerability data at defined time points; review of this data may also lead to further decisions being made by the DMC.

Further details of the IAs and the decision rules to be implemented will be detailed in the statistical analysis plan (SAP) and the DMC Charter.

Sample size

The study is proof of concept, designed to allow for a considered testing of a range of doses of AP30663, compared with placebo. Based on the study design, the sample size for randomisation will range from up to 36 patients (for Part 1) to up to 108 patients (for Parts 1 and 2 cumulatively). The total sample size is dependent on the AP30663 dose arms opened on the decision of the DMC. A total of 4 IAs and a final analysis are

included in the design, and so it is foreseen that the total number of randomised patients will be up to 36, 54, 72, 90, or 108 to have sufficient numbers of patients evaluable for AF conversion at each IA.

The adaptive design utilises the probability that the response rate at a dose d is greater than a success rate of 65% ($P_d > 0.65$, where d is the dose arm of AP30663), based on a Bayesian modelling procedure.

4 TABLE OF CONTENTS

1 PROTOCOL APPROVAL SIGNATURES ... 2

2 STUDY PERSONNEL ... 6

3 SYNOPSIS ... 8

4 TABLE OF CONTENTS ... 15

4.1 List of In-text Tables ... 18
4.2 List of In-text Figures ... 18

5 LIST OF ABBREVIATIONS ... 19

6 INTRODUCTION ... 21

7 STUDY OBJECTIVES AND ENDPOINTS ... 24

7.1 Study Objectives ... 24
7.1.1 Primary Objective ... 24
7.1.2 Secondary Objectives ... 24
7.1.3 Exploratory Objectives ... 24
7.2 Study Endpoints ... 24
7.2.1 Primary Endpoint ... 24
7.2.2 Secondary Endpoints ... 25
7.2.2.1 Efficacy Endpoints ... 25
7.2.2.2 Safety Endpoints ... 25
7.2.2.3 PK Endpoints ... 25
7.2.3 Exploratory Endpoints ... 25

8 INVESTIGATIONAL PLAN ... 26

8.1 Overall Study Design and Plan: Description ... 26
8.1.1 Study Design ... 29
8.1.2 Schedule of Assessments ... 30
8.2 Discussion of Study Design ... 33
8.2.1 Study Rationale ... 33
8.2.2 Quality Management and Risk Evaluation ... 34
8.3 Selection of Study Population ... 34
8.3.1 Number of Planned Patients ... 34
8.3.2 Inclusion Criteria ... 34
8.3.3 Exclusion Criteria ... 35
8.3.4 Removal of Patients From Therapy or Assessments ... 37

8.3.4.1 Patient Discontinuation/Withdrawal From the Study 37
8.3.4.2 Treatment Discontinuation (Stop of Infusion) 38

8.4 Investigational Products 39

8.4.1 Investigational Products Administered 39
8.4.2 Identity and Preparation of Investigational Products 40
8.4.3 Packaging and Labelling 41
8.4.4 Method of Assigning Patients to Treatment Groups 42
8.4.5 Selection of Doses in the Study.. 42
8.4.6 Selection and Timing of Dose for Each Patient 42
8.4.7 Blinding... 42
8.4.8 Prior and Concomitant Therapy 43
8.4.8.1 Prohibited Medication and Procedures... 44
8.4.8.2 Rescue Medication 46
8.4.9 Treatment Compliance 46

9 TIMING OF STUDY PROCEDURES... 47

9.1 Screening Visit 47

9.2 Day 1 – Pre-infusion (Baseline) 48

9.3 Day 1 – During Infusion... 48

9.4 Day 1 – Post-Infusion.. 49

9.5 Day 2 50

9.6 Follow-up/Discontinuation Visit (Day 30)... 50

9.7 Duration of Treatment 50

10 EFFICACY, PHARMACOKINETICS, AND SAFETY VARIABLES.. 51

10.1 Efficacy, Pharmacokinetics, and Safety Measurements.................................. 51

10.1.1 Efficacy Variables 51
10.1.1.1 24-Hour Holter 51
10.1.2 Pharmacokinetic Variables.. 52
10.1.2.1 Plasma Concentrations of AP30663... 52
10.1.3 Safety Assessments 52
10.1.3.1 Adverse Events... 52
10.1.3.2 Clinical Laboratory Evaluation 57
10.1.3.3 Vital Signs.. 58
10.1.3.4 Physical Examination and Brief Physical Examination 58
10.1.3.5 Electrocardiogram 58

10.2 Independent Data Monitoring Committee.. 59

10.3 Appropriateness of Measurements 60

11 STATISTICAL METHODS ... 61
11.1 Statistical and Analytical Plans ... 61
11.1.1 Datasets or Populations Analysed ... 61
11.1.2 Demographic and Other Baseline Characteristics ... 62
11.1.3 Efficacy Variables ... 62
11.1.4 Safety Variables ... 63
11.1.5 PK Variables ... 64
11.1.6 Interim Analyses ... 66
11.1.7 Handling of Missing Data ... 67
11.2 Determination of Sample Size ... 67
11.3 Protocol Deviations ... 68
12 QUALITY ASSURANCE AND QUALITY CONTROL ... 69
12.1 Audit and Inspection ... 69
12.2 Monitoring ... 69
12.3 Data Management and Coding ... 69
12.4 Quality Management and Risk Evaluation ... 69
13 RECORDS AND SUPPLIES ... 70
13.1 Drug Accountability ... 70
13.2 Financing and Insurance ... 70
14 ETHICS ... 71
14.1 Independent Ethics Committee ... 71
14.2 Regulatory Authorities ... 71
14.3 Ethical Conduct of the Study ... 71
14.4 Informed Consent ... 71
14.5 Patient Confidentiality ... 72
15 REPORTING AND PUBLICATION, INCLUDING ARCHIVING ... 73
16 REFERENCES ... 74
17 APPENDICES ... 75
17.1 Investigator Signature Page ... 75
17.2 Blood Sampling ... 76
17.3 Distribution of Patients Scenarios ... 77

4.1 List of In-text Tables

Table 1 Composition of AP30663 Concentrate for Solution for Infusion ... 40
Table 2 Prohibited Medication ... 45
Table 3 Physical Examination ... 58

4.2 List of In-text Figures

Figure 1 Study Schema ... 29

5 LIST OF ABBREVIATIONS

AE	Adverse event
AERP	Atrial effective refractory period
AF	Atrial fibrillation
ALT	Alanine aminotransferase
APTT	Activated partial thromboplastin time
AST	Aspartate aminotransferase
AUC	Area under the concentration time curve
AV	Atrioventricular
BBB	Bundle branch block
BP	Blood pressure
bpm	Beats per minute
BUN	Blood urea nitrogen
Ca	Calcium
CABG	Coronary artery bypass graft
C_{max}	Observed peak plasma concentration
CYP	Cytochrome P450
DC	Direct-current
DMC	Data monitoring committee
ECG	Electrocardiogram
eCRF	Electronic case report form
EDC	Electronic data capture
eGFR	Estimated glomerular filtration rate
EMA	European Medicines Agency
EU	European Union
FDA	Food and Drug Administration
GCP	Good Clinical Practice
GGT	Gamma-glutamyltransferase
GLP	Good Laboratory Practice
IC_{50}	Half maximal inhibitory concentration
ICF	Informed consent form
ICH	International Council for Harmonisation
IEC	Independent ethics committee
INR	International normalised ratio
IRAF	Immediate relapse of atrial fibrillation

IWRS	Interactive web randomisation system
Kel	Elimination rate constant
LS	Least squares
LV	Left ventricle
LVEF	Left ventricular ejection fraction
MedDRA	Medical Dictionary for Regulatory Activities
MTD	Maximum tolerated dose
NOAEL	No observed adverse effect level
PCI	Percutaneous coronary intervention
PD	Pharmacodynamics
PK	Pharmacokinetic
QTc	Corrected QT interval
SAE	Serious adverse reaction
SAP	Statistical analysis plan
SD	Standard deviation
SOC	System organ class
SOP	Standard operating procedure
SR	Sinus rhythm
SUSAR	Suspected unexpected serious adverse reaction
$t_{1/2}$	Terminal half-life
TEAE	Treatment-emergent adverse event
T_{max}	Time at which C_{max} occurs
TSH	Thyroid stimulating hormone
TTE	Transthoracic echo
WHO	World Health Organisation

6 INTRODUCTION

Atrial fibrillation (AF) is the most common cardiac arrhythmia (irregular heart beat) and is characterised by chaotic electrical signals in the upper chambers of the heart. This is seen as a very rapid frequency and malfunctioning contraction in the atria that may lead to impaired quality of life, reduced work productivity, and increased rate of hospitalisation. A severe consequence of AF is a 5-fold increased risk of stroke and 1.9-fold increased mortality. AF-related strokes account for up to 20% of all strokes and are therefore a major cause of this debilitating and often fatal disease. AF has reached epidemic proportions and affects up to 5 million people in the European Union (EU). Prevalence of AF has grown to 2% of the population, double that reported in the last decade, and the estimated number of patients with AF in 2030 in the EU will have grown to 14 to 17 million (Zoni-Berisso et al. 2014). Risk of AF increases with age, and approximately 70% of patients suffering from AF are between 65 and 85 years of age (Feinberg et al. 1995). The latest European AF treatment guidelines from the European Society of Cardiology (Kirchhof et al. 2016) favour the principle “safety first”, implying that the recommended first-line agents are only moderately effective. There is currently no gold standard treatment for AF in the EU and a large unmet medical need exists for developing and introducing novel compounds for treating the increasing number of AF patients.

AP30663 is being developed by Acesion Pharma for the conversion of AF by a 30-minute intravenous infusion in patients with episodes of AF. AP30663 is a small molecule drug product candidate able to inhibit the small-conductance Ca^{2+}-activated potassium channels (SK channels), a novel and functionally atrial-selective ion channel target for the treatment of AF.

Mechanistically, AP30663 inhibits human SK channels at sub-micromolar concentrations with an in vitro half maximal inhibitory concentration (IC_{50}) (SK3) of 0.77 µM. In pigs, AP30663 has demonstrated significant and pronounced prolongation of the atrial effective refractory period and ability to convert AF to normal sinus rhythm (SR) while at the same time being well tolerated and not prolonging the QT interval significantly.

Pharmacokinetic (PK) studies in landrace pigs, as well as the Good Laboratory Practice (GLP) safety studies conducted in rat and minipig, demonstrate that AP30663 is rapidly distributed in plasma after start of infusion and that an apparent steady-state plasma level is reached well before the end of the 30- to 60-minutes continuous infusion. Immediately after the end of the infusion, the plasma levels drop rapidly due to the kinetics of the distribution phase. From a safety-risk assessment viewpoint, this fast and significant drop of plasma concentration after termination of the infusion is considered a means to help effectively minimise transient C_{max}-related adverse effects, should they occur.

Binding of AP30663 to plasma proteins has been determined in vitro for a number of species and plasma protein binding of 93.2% was determined in human plasma. In comparison, plasma protein binding in the 2 GLP safety-toxicology species rat and

minipig were 92.6% and 79.9%, respectively. For comparison, the plasma protein binding in landrace pigs was 86.4%.

The AP30663 drug product is a concentrate for solution for infusion containing 200 mg/mL AP30663. The drug concentrate should be stored in the original container at -15ºC to -25ºC until 30 to 60 min before start of preparation. The drug concentrate is diluted with sterile 5% glucose solution for infusion to a concentration of 2 mg/mL prior to administration. The final solution for infusion must be stored at room temperature and must be used within 24 h after start of preparation.

In the conducted GLP toxicology programme, AP30663 was not associated with any adverse toxicity in the doses and exposures tested, except for infusion site reactions in the rat. The single dose maximum tolerated dose (MTD) in minipigs was defined as 40 mg/kg, at which dose one episode of convulsion appeared in one minipig 50 min into an intended 60-minute infusion period. In rats, the single dose MTD was defined as 90 mg/kg based on a combination of signs and symptoms, i.e. decreased activity, piloerection, semi-closed eyes, and dyspnoea.

The no observed adverse effect level (NOAEL) in minipig after 2 weeks of daily single dose intravenous treatment with AP30663 was 25 mg/kg/day, which was the highest dose tested.

In rats, daily 1-hour infusions for 2 weeks caused a reversible dose-related local infusion site inflammation at all dose levels, with associated lung inflammation and thromboembolism. No other toxicity of human relevance was observed up to the highest dose tested, 90 mg/kg/day, which was considered the NOAEL for toxicity not related to the local infusion site reactions.

The maximally tolerated exposure in minipigs and rats appeared to be comparable, meaning that the 2 species may be regarded as similarly sensitive. A total (free and bound) exposure level (C_{max}) of approximately 11,000 ng/mL was seen at the highest dose in both minipigs (25 mg/kg) and rats (90 mg/kg), and this dose level should not be exceeded in humans unless carefully evaluated.

Two phase 1 Single Ascending Dose trials investigating the safety and tolerability of AP30663 in healthy male subjects at doses up to 8 mg/kg have been completed. The trials showed that the infusion with AP30663 caused mild and transient infusion site reactions as well as prolongation of the QTcF-interval but was otherwise well tolerated at all administered dose levels. The effect on QTcF was dose and plasma concentration related with a peak estimated mean QTcF prolongation of 45.2 ms for the dose of 6 mg/kg. The QTcF effect was transient and a return to baseline level was observed 8 hours after the start of administration for the 6 mg/kg dose, consistent with low plasma concentrations at this timepoint.

No effects were found on heart rate, blood pressure or other ECG markers. No CNS effects were found, including based on a sensitive assessment of tremors (tremorography).

The aim of this study is to show proof of concept, (i.e. the ability of AP30663 to terminate ongoing AF episodes) and 1 or more dose levels will be compared with placebo.

7 STUDY OBJECTIVES AND ENDPOINTS

7.1 Study Objectives

7.1.1 Primary Objective

The primary objective of the study is to demonstrate the efficacy of 1 or more dose levels of AP30663 on the basis of the ability to convert AF after intravenous administration.

7.1.2 Secondary Objectives

The secondary objectives of the study are as follows:

- To study the stability of rhythm control (immediate relapse of AF [IRAF], i.e. within 5 min after conversion from AF).
- To study the importance of AF duration with respect to the efficacy and safety of 1 or more dose levels of AP30663.
- To evaluate the safety and tolerability of 1 or more dose levels of AP30663.
- To study the relationship between systemic exposure and response, with special regard to the conversion from AF and the effect on QRS and QTcF.

7.1.3 Exploratory Objectives

The exploratory objectives of the study are as follows:

- To study demographic and echocardiographic variables, concomitant diseases, and concurrent medication with respect to the efficacy and safety of 1 or more dose levels of AP30663.
- To evaluate the PK of AP30663, including influence of dose, concomitant medication, concurrent diseases, and demographic variables.
- To explore the proportions of patients on AP30663 converting from AF and of patients randomised to placebo and converting at direct-current (DC) cardioversion.

7.2 Study Endpoints

7.2.1 Primary Endpoint

The proportion of patients that have converted from AF within 90 min from the start of infusion and subsequently have no AF recurrence within 1 min of conversion from AF.

7.2.2 Secondary Endpoints

7.2.2.1 Efficacy Endpoints

- The time to conversion from AF from start of infusion.
- The proportion of patients with relapse of AF within 5 min (IRAF) after pharmacological or DC cardioversion.
- The proportion of patients in SR at 3 h ± 1 h after start of infusion.
- The proportion of patients in SR at 24 h ± 2 h after start of infusion.
- The proportion of patients in SR at 30 days ± 5 days after start of infusion.

7.2.2.2 Safety Endpoints

- Adverse events (AEs) and electrocardiogram (ECG) variables, including significant arrhythmia, physical examination, vital signs, and laboratory evaluations.
- Changes in QTcF interval data over time.

7.2.2.3 PK Endpoints

- Systemic exposure derived from the population PK model.
- Population PK model parameter estimates derived from plasma concentrations of AP30663.

7.2.3 Exploratory Endpoints

- Correlation of drug exposure (plasma C_{max} and area under the concentration time curve [AUC]) and the time of conversion.
- Correlation of atrial size and conversion rate.
- Correlation of atrial size to relapse or failure.
- Correlation of gender and age and conversion rate.
- Correlation of duration of current AF, conversion rate and duration of SR after conversion.

8 INVESTIGATIONAL PLAN

8.1 Overall Study Design and Plan: Description

This is a double-blind, randomised, placebo-controlled, parallel-group Phase 2a study of 1 or more doses of AP30663 for cardioversion in adult patients with AF. The study will be conducted in 2 parts (Part 1 and 2): Part 1 of the study is a fixed randomisation placebo-controlled parallel design, and Part 2 will be an adaptive design conducted in 1 or more further doses of AP30663 versus placebo. The study is designed to identify the dose of AP30663 to be evaluated further in later-phase research.

For both parts of the study, qualified patients will be randomised (Day 1) as soon as it is practically possible after providing their written informed consent and completion of screening procedures, but no later than 7 days after onset of the AF episode. The study drug (AP30663 or placebo) will be administered as a single intravenous infusion for 30 min on Day 1. Administration should take place as fast as possible after randomisation, and preferably no later than 12 h after randomisation. Patients may be discharged from the study site after all Day 1 procedures are completed. They will return to the study site the following day (Day 2) and for a follow-up visit on Day 30.

In Part 1 of the study, up to 36 patients will be randomised in a 1:1 ratio to receive AP30663 at 3 mg/kg or matching placebo. Their participation will last until the completion of their follow-up visit at Day 30. An interim analysis (IA) will be conducted once the randomised patients in Part 1 have completed Day 2 assessments or terminated the study. An independent data monitoring committee (DMC) will be convened to review the unblinded efficacy (including ECG and Holter ECG), PK/PD, safety and tolerability data during this IA. The recommendations of the DMC at the IA will determine the design and starting dose(s) for assessment during Part 2 of the study; the DMC may also recommend that the study to be stopped after Part 1.

Part 2 of the study is an adaptive design, conducted in a minimum of 18 and a maximum of 72 patients, in 1 or more doses of AP30663 (Dose [d] where d = 2, 4, 5, or 6 mg/kg) or placebo (d = 0). The maximum dose of 6 mg/kg AP30663 should not be exceeded under any circumstances, and the doses assessed during Part 2 will depend upon the cumulative results achieved across both Part 1 and Part 2. The IAs during Part 2 will occur after cohorts of 18 randomised patients have completed Day 2 assessments or terminated the study; thus, IAs will occur when 18, 36, and 54 randomised patients have completed Day 2 assessments or terminated the study (i.e. up to 54, 72, 90, and 108 patients cumulatively).

During the enrolment period, an IA may be planned on a reduced cohort if preliminary blinded safety and efficacy data indicate that current enrolment is sufficient for the DMC to provide a recommendation for Part 2.

During Part 2, dose decisions made by the DMC at each IA is based primarily on the AF conversion response. Any efficacy criteria assessed at each IA will also be

supplemented with safety review by the DMC, at aligned timelines with the IAs described. Further details of DMC review will be provided in the DMC Charter.

Randomisation during Part 2 of the study will be in 2:1, where all active arms will be randomised equally and cumulatively. The potential doses of AP30663 that could be assessed (randomised to) during Part 2 are 2, 4, 5, or 6 mg/kg (in addition to placebo). For example, if Doses 2 and 5 are open then randomisation will be 1:1:1 for Dose 2: Dose 5: Placebo for the cohort of 18 patients to be enrolled (i.e. 2:1 in total for AP30663 versus Placebo). If 1 dose of AP30663 is open versus placebo, then the randomisation will be 2:1.

Algorithm for Dose Assessment During Part 2

The following algorithm will be used for selection of dose levels of AP30663 during Part 2 of the study. For each analysis, the success rate for any single dose level d, P_d, will be analysed using a Bayesian model, whereby the prior probability of success at a dose d is modelled with a uniform Beta prior, and the posterior distribution is modelled for each dose independently using a Beta posterior distribution. The design utilises the probability that the response rate at a dose is greater than a 65% success rate ($P_d > 0.65$). Further details are provided in Section 17.3.

Interim Analysis at End of Part 1:

During the first IA, conducted once up to 36 patients randomised in Part 1 have completed Day 2 assessments or terminated the study, the following will occur, regardless of the response seen for the AP30663 3 mg/kg dose:

- Further randomisation to Dose 3 (3 mg/kg AP30663) will be stopped.
- Dose 5 (5 mg/kg AP30663 versus placebo) will be opened.

Additionally, the following decision will be made dependent on the response rate seen for 3 mg/kg AP30663:

- If the proportion of patients achieving a response with AP30663 3 mg/kg is > 0.65 then Dose 2 (2 mg/kg AP30663) will additionally be opened for Part 2 (in parallel to Dose 5).

Interim Analyses During Part 2:

Interim analyses will occur when 18, 36, and 54 randomised patients have completed Day 2 assessments or terminated the study in Part 2. At these IAs the following rules will be utilised:

a) If an open Dose d has at least 10 randomised patients who have completed Day 2 assessments and satisfies: ($Pd > 0.65$) ≥ 0.90 then Dose d will be closed for sufficient efficacy response.
    - If the dose one level below this dose has never been opened, then dose d-1 is opened.

b) If an open Dose d has at least 10 randomised patients who have completed Day 2 assessments and satisfies ($Pd > 0.65$) < 0.10 then Dose d will be closed.

- If Dose d was the largest dose thus far open, and Dose d is < 6 mg/kg, then dose d+1 will be opened.

During Part 2, all active doses will be equally randomised; for each of these IAs, randomisation will occur 2:1 across all open AP30663 doses combined versus placebo. For further details/examples of decision scenarios during Part 2, please see Section 17.3.

The study will continue until all doses of AP30663 have been closed or the maximum of 72 patients during Part 2 (up to 108 patients total across Parts 1 and 2) have been enrolled. Further details of the IAs and the decision rules to be implemented will be detailed in the statistical analysis plan (SAP).

8.1.1 Study Design

Figure 1 Study Schema

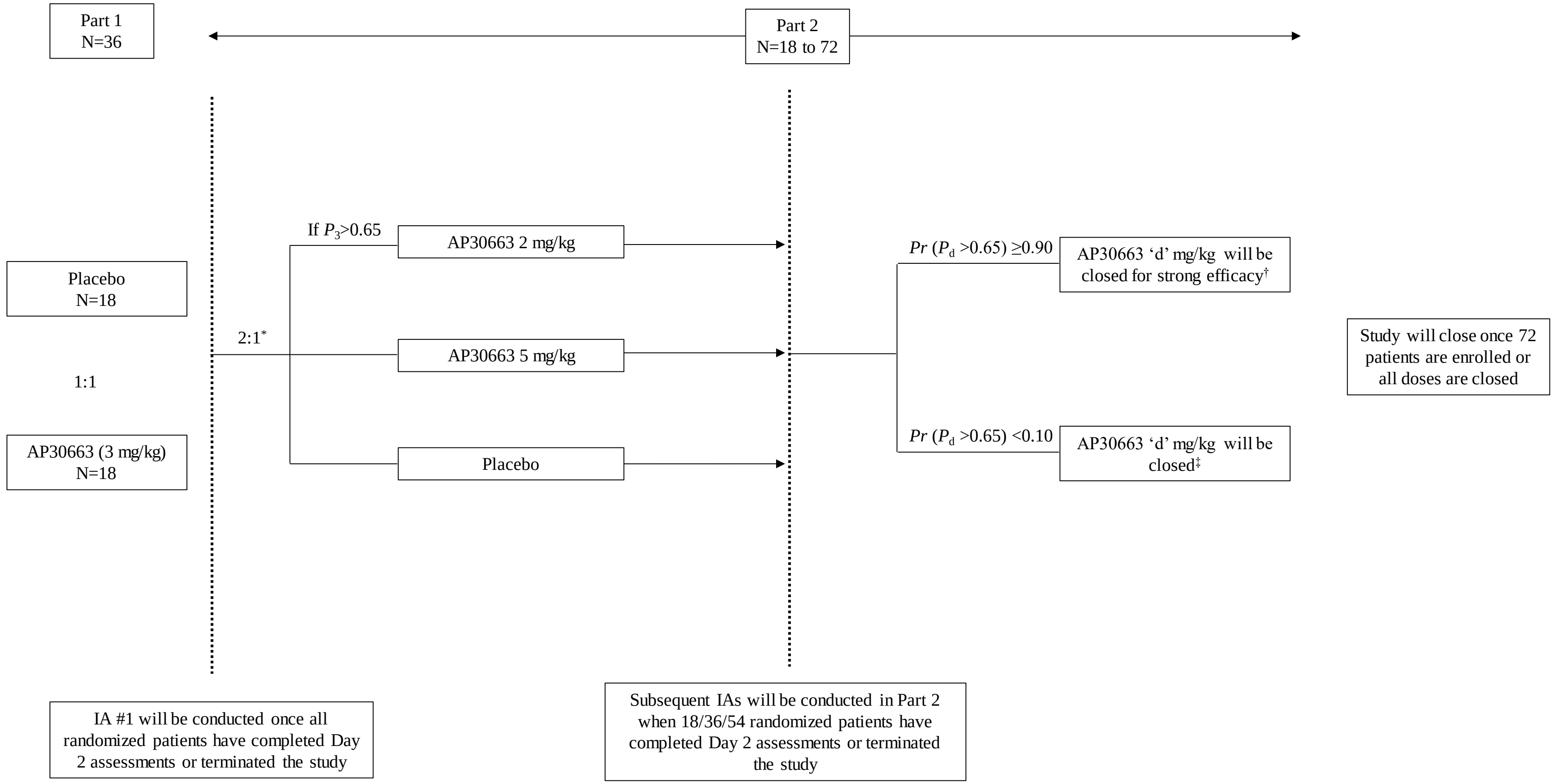

Abbreviations: N=number of patients enrolled, P_3=conversion ratio at 3 mg/kg, P_d=conversion ratio at d mg/kg, *P*r=probability.
*All active AP30663 arms will be randomised equally for each interim analysis; randomisation will occur cumulatively in 2:1 ratio to receive either AP30663 or placebo across all open AP30663 arms.
†If one level below this dose (d-1) has never been opened, then dose d-1 will be opened
‡If dose 'd' is the highest dose opened so far, and dose 'd' is <6 mg/kg, then dose 'd+1' will be opened.

8.1.2 Schedule of Assessments

The schedule of the planned study assessments during Part 1 is shown in the following table. The planned study assessments during Part 2 are same to those that are planned for Part 1.

	Screening (within 7 days of onset of AF)	Day 1	Day 1	Day 1 [a]	Day 2	Day 30 (± 5 days) Follow-up/ Discontinuation Visit
		Baseline (pre-infusion)	During infusion	Post-infusion		
Informed consent	X					
Inclusion/exclusion criteria	X	X				
Demographics	X					
Medical/surgical history	X					
Concomitant medications	X	X	X	X	X	X
Physical examination [b]	X					
Brief physical examination [c]		X				X
Vital signs (BP, heart rate, heart rhythm from ECG)	X	X	X	X	X	X
Weight & height	X					
Haematology [d]	X	X [e]				X
Clinical chemistry [f]	X	X [e]			X	X
Serum magnesium (Mg++), sodium (Na+), calcium (Ca++)	X					
Serum potassium (K+) [h]	X					
Urea (BUN)	X					X
INR/APTT [g]	X	X [i]				X
TSH	X [j]					
Randomisation [k]		X				
Study drug administration [l]			X			

	Screening (within 7 days of onset of AF)	Day 1	Day 1	Day 1 [a]	Day 2	Day 30 (± 5 days) Follow-up/ Discontinuation Visit
		Baseline (pre-infusion)	During infusion	Post-infusion		
PK sampling [m]		X	X	X	X	
12-lead ECG (digital)	X	X [n]	X [n]		X [n]	X
Holter [o]		◄--		------►		
Telemetry [o]		◄--		------►		
Transthoracic echo (TTE) [p,q]	X					
Electrical cardioversion				X [r]		
AE/SAE [s]	X	X	X	X	X	X

Abbreviations: AE = adverse event, AF = atrial fibrillation, ALT = alanine aminotransferase, APTT = activated partial thromboplastin time, AST = aspartate aminotransferase, BP = blood pressure, BUN = blood urea nitrogen, ECG = electrocardiogram, eGFR = estimated glomerular filtration rate, GGT = gamma-glutamyltransferase, ICF = informed consent form, INR = international normalised ratio, LV = left ventricle, LVEF = left ventricular ejection fraction, PK = pharmacokinetic, SAE = serious adverse event, TSH = thyroid stimulating hormone

a Patients may be discharged after all Day 1 procedures are completed and will return to the study site for a visit the following day (Day 2)

b Physical examination includes general appearance, head, ears, eyes, nose, throat, cardiovascular, respiratory, gastrointestinal, dermatological, neurological, musculoskeletal, lymphatic, and other at the discretion of the investigator.

c Brief physical examination includes general appearance, cardiovascular, respiratory, gastrointestinal, and other at the discretion of the investigator.

d Haematology parameters include haemoglobin, haematocrit, red blood cells (erythrocytes), white blood cells (leukocytes, total and differential), basophils, eosinophils, lymphocytes, monocytes, neutrophils, and platelets.

e Additional haematology and clinical chemistry parameters will be evaluated if not already assessed <72 h before start of infusion.

f Clinical chemistry parameters include ALT, AST, bilirubin creatinine, GGT, glucose (random), and eGFR.

g Only one coagulation parameter (either INR or APTT) is required for an individual patient. The same coagulation parameter should be consistently analysed throughout the study in an individual patient.

h Patients with low potassium levels at screening may be supplemented with potassium. A retest is required before randomisation.

i To be repeated for all patients on heparin or vitamin K antagonists when screening >24 h before start of infusion.

j If TSH levels were tested within 1 week before randomisation at a local laboratory certified for the study, those results will be acceptable and should not be re-tested at screening.

k Randomisation must occur as soon as possible between 3 h and 7 days (inclusive) after start of the current AF episode. Each randomised patient will be assigned a patient number. Note, if the patient is found eligible, the randomisation should be planned in such a way that all logistics will occur in the protocol-specified timeframes including timely reconstitution of study drug, infusion start and, if applicable, direct-current cardioversion in non-responders.

l	Study drugs must be administered as soon as possible after randomisation and preferably not later than 12 h after randomisation.
m	Plasma samples will be taken at baseline (pre-infusion) and at the following time points after start of infusion: 5 min ± 1 min, 15 min ± 1 min, 25 min ± 1 min, 30 min - 1 min (the infusion will not be stopped before the 30-min PK sample has been collected), 45 min ± 5 min, 1 h ± 5 min, 1 h 30 min ± 5 min, 4 h ± 5 min, 8 h ± 5 min, and 24 h ± 2h. In the case of conversion from atrial fibrillation within 90 min from the start of infusion, an additional sample will be taken immediately after conversion.
n	ECG print-outs: within 15 min before infusion start, at 10 min after infusion start, and, in case of conversion from AF, within 1 min after conversion BUT before infusion stop. Diagnosis of AF (defined as absence of discrete P waves and an irregular ventricular rate) to be confirmed based on a 12-lead ECG. On Day 2 the ECG should be completed 24 h +/- 2 hours after infusion start.
o	Continued assessment to start 30 min before infusion. Holter to stop after the PK sampling at 8 h. Telemetry to continue for minimum 8 h after infusion start.
p	Should be done in all patients; mandatory if not done within 6 months (inclusive) before randomisation.
q	Heart rhythm, LV size, LV area, LVEF, atrial area and size as well as other clinically significant finding results to be provided or requested if available.
r	In case of AF persistence at 90 min after start of infusion, a direct-current electrical cardioversion is to be done within 180 min post-infusion start (200 J biphasic synchronised).
s	Recording of any AEs/SAEs will start after the ICF has been signed. From infusion start, special attention should be paid to AEs related to the central nervous system including tremors.

8.2 Discussion of Study Design

8.2.1 Study Rationale

This is a proof-of-concept study aiming to show the ability of AP30663 to terminate ongoing AF episodes.

The study was designed to allow for modifications to the AP30663 dose without undermining the validity and integrity of this study. After all randomised patients have completed Day 2 assessments of the study (or terminated), an interim analysis will be conducted to determine whether or not the study can be continued to Part 2 and, if to be continued, to determine the starting dose(s) of AP30663 to be used in Part 2 of the study. Further IAs will be conducted during Part 2 to allow for additional doses to be tested, or poor-performing doses to be stopped. This is expected to determine a dose of AP30663 with adequate efficacy and minimal adverse effects. No Type II error correction will be taken into account due to this being a proof-of-concept study.

Choice of placebo comparator

Currently, there is no gold standard treatment for AF in the EU and the recommended first-line agents are only moderately effective. A placebo group was chosen as control group to prevent bias and as there is no standard treatment for AF in the EU. Treatment with placebo is short (30 min infusion) and patients will receive DC electrical cardioversion if AF is still ongoing 90 min after the infusion start.

Rationale for dose selection

In the completed phase 1 trials a dose and plasma concentration related effect on QTcF was found with a peak estimated mean QTcF prolongation of 45.2 ms for the dose of 6 mg/kg. The QTcF effect was transient and a return to baseline level was observed 8 hours after the start of administration for the 6 mg/kg dose consistent with low plasma concentrations at this timepoint. No other dose related effects were found.
For the starting dose of 3 mg/kg preclinical data support the assumption that it will result in a sufficiently high free plasma concentration to be effective in conversion.

The increase of the QTcF would serve as a safety parameter, but might also be a marker of a higher increase of the atrial effective refractory period (AERP) and thereby be indicative of a higher chance of conversion. In this study, the continuous monitoring of the patients during and after the 30-minute infusion in combination with predefined stopping criteria (see Section 8.3.4.2) will guarantee the safety of the patients.

The low-dose alternative of 2 mg/kg is the lowest AP30663 dose that produced a free plasma concentration at which it is reasonable to expect an effect on conversion. The lower dose of 2 mg/kg AP30663 may be an option, if the starting dose shows excellent or good efficacy but insufficient safety or tolerability.

Details regarding reasonably anticipated adverse events (AEs) as well as known or anticipated benefits and risks for AP30663, may be found in the investigator’s brochure.

8.2.2 Quality Management and Risk Evaluation

This protocol was evaluated to identify those processes and data that were critical to ensure human patient protection and reliability of study results.

Risk control measures will be periodically reviewed to ascertain whether the implemented quality management activities remain effective and relevant, taking into account emerging knowledge and experience.

8.3 Selection of Study Population

8.3.1 Number of Planned Patients

Approximately 41, 62, 82, 103, or 123 patients will need to be screened in approximately 15 centres in EU to achieve up to 36, 54, 72, 90, or 108 randomised patients, respectively, who have completed Day 2 assessments or terminated the study, depending on the study design and recommendation of the DMC at each of the IAs. The number of patients screened is based on a screen failure rate of 12%.

Examples of scenarios for the distribution of patients across dose arms during Part 1 and Part 2 are provided in Section 17.3.

The statistical considerations on which the sample size is based are provided in Section 11.2. Patient replacement is described in Section 8.3.4.1.

8.3.2 Inclusion Criteria

To be eligible for study entry, patients must satisfy all of the following criteria:

1. Provision of written informed consent.
2. Clinical indication for cardioversion of atrial fibrillation.
3. Current episode of symptomatic atrial fibrillation lasting between 3 h and 7 days inclusive at randomisation.
4. Adequate anticoagulation according to international and/or national guidelines.
5. Body weight 50 to 110 kg, inclusive (with clothes, without shoes).
6. Male patients and postmenopausal women aged 18 to 80 years, inclusive.
   - Male patients who are sexually active must agree to abstain from sexual activity or be willing to use a double-barrier method of birth control (i.e. any double combination of male or female condom with spermicidal gel, diaphragm, sponge or cervical cap with spermicidal gel) if they become

sexually active from the time of consent and for 90 days after the infusion day.

- Post-menopausal women are defined as being >12 months after last menstrual period.
- Women can also be included if permanently sterilised since ≥6 weeks (i.e. documented hysterectomy, bilateral salpingectomy, bilateral oophorectomy). Breastfeeding women are excluded.

8.3.3 Exclusion Criteria

Patients will be excluded from the study if 1 or more of the following criteria are applicable:

1. Significant clinical illness or surgical procedure within 4 weeks preceding the screening visit.
2. Present renal dysfunction (estimated glomerular filtration rate [eGFR] < 30 mL/min), hepatic dysfunction (alanine aminotransferase [ALT] or aspartate aminotransferase [AST] > 3 × upper limit of normal), or uncontrolled hyperthyroidism or hypothyroidism.
3. History of significant mental, renal or hepatic disorder, chronic obstructive pulmonary disease or other significant disease, as judged by the investigator.
4. Any cardioversion attempt of AF or atrial flutter within 1 week preceding randomisation.
5. Prior failed attempt (no conversion) of pharmacological or DC cardioversion of previous or current AF episode.
6. Failure to find a large antecubital (or equivalent) vein for the infusion.
7. Any of the following events, or any other significant cardiovascular event as judged by the investigator, during the last 6 weeks before randomisation: myocardial infarction, unstable angina pectoris or other signs of myocardial ischaemia, stroke or transient ischaemic attack, myocardial revascularisation (percutaneous coronary intervention [PCI], coronary artery bypass graft [CABG]), or other revascularisation procedure.
8. Haemodynamically unstable condition as judged by the investigator; systolic BP <90 mm Hg or >180 mm Hg, or diastolic BP >105 mm Hg at randomisation.
9. Blood haemoglobin <100 g/L at screening.
10. Congestive heart failure New York Heart Association class III or IV. Left ventricular ejection fraction <40% on echocardiography, or other clinically significant abnormality on the echocardiogram (not older than 6 months) as judged by the investigator.

11. Known hypertrophic cardiomyopathy or significant left ventricular hypertrophy (free wall or septal thickness >13 mm).
12. Any clinically significant valvular heart disease.
13. History or previous signs of sinus nodal disease
14. Pacemaker or implantable cardioverter defibrillator therapy.
15. Personal or family history of Torsades de Pointes, any other polymorphic ventricular tachycardia, sustained ventricular tachycardia, long QT syndrome, and/or Brugada syndrome.
16. QTc (Fridericia, QTcF) interval >450 ms at randomisation. (When measured during AF, the mean heart rate should be 50 to 100 bpm. The QTcF should be calculated at AF as the mean of at least 5 consecutive RR intervals with consecutive QT intervals).
17. QRS duration >120 ms at randomisation.
18. Known atrioventricular (AV)-block I (prolonged PQ [PR] interval >220 ms), AV-block II, AV-block III, or complete bundle branch block (BBB) .
19. Potassium in serum below 3.5 or above 5.3 mmol/L at randomisation. Patients with low potassium levels at screening may be appropriately supplemented with potassium before baseline, according to the local standards. A re-test of the potassium level is required, and the patient can be randomised after the potassium has returned to reference range.
20. Anticipated change in dose or initiation of loop diuretic from screening to the end of infusion.
21. Use of any antiarrhythmic drug class I and/or III within 7 days or, for amiodarone specifically, 12 weeks before randomisation.
22. Use of QT-prolonging drug, and/or drug that inhibits cytochrome P450 (CYP)3A4, as well as St John's Wort within 10 days before randomisation.
23. Administration of an investigational drug within the preceding 3 months before randomisation.
24. Administration of AP30663 at any time before randomisation.
25. History of drug addiction and/or alcohol abuse within the last 12 months at the discretion of the investigator.
26. Blood or plasma donation within the preceding 4 weeks before randomisation.
27. Any suspected or manifested clinically significant infection as judged by the investigator.
28. Involvement in the planning and conduct of the study (applies to Acesion Pharma staff, Syneos Health staff, and staff at the investigational site).

29. Clinical judgement by the investigator that the patient should not participate in the study.
30. Any malignant cancer within 3 years (except for successfully treated in-situ non-melanoma skin cancer and in-situ cervical cancer) of signing the informed consent form (ICF).

8.3.4 Removal of Patients From Therapy or Assessments

8.3.4.1 Patient Discontinuation/Withdrawal From the Study

Patients may be withdrawn from the study for any of the following reasons:

- Patient request.
- Patient non-compliance with the study protocol.
- Use of non-permitted concurrent therapy.
- Lost to follow-up.
- Occurrence of adverse events (AEs) not compatible with the continuation of patient participation in the study, in the investigator’s opinion, or unacceptable to the patient to continue.
- If the pre-infusion ECG QTcF is above 450 ms the infusion with the IMP should not be initiated and the patient should be registered as an Early Termination.
- Investigator request.
- Request by Acesion Pharma.

<u>Patient replacement</u>

- Patients who spontaneously convert to SR before randomisation are screen failures. Re-screening of such patients is not allowed.
- Patients who spontaneously convert to SR after randomisation and before infusion start are considered dropouts and will be replaced.
- Patients who have a QTcF above 450 ms at pre-infusion.
- Patients who do not meet inclusion criterion #2 (clinical indication for cardioversion of AF) will be replaced. Patients who stop study drug for any other reason will not be replaced.

Patients are free to withdraw from the study at any time without providing reason(s) for withdrawal and without prejudice to further treatment. The reason(s) for withdrawal will be documented in the electronic case report form (eCRF).

Patients withdrawing from the study will be encouraged to complete the same final evaluations as patients completing the study according to this protocol, particularly safety evaluations. The aim is to record data in the same way as for patients who completed the study.

Reasonable efforts will be made to contact patients who are lost to follow-up. These efforts must be documented in the patient's file.

Acesion Pharma has the right to terminate the study at any time in case of serious AEs (SAEs) or if special circumstances concerning the investigational product or the company itself occur making further treatment of patients impossible. In this event, the investigator(s) will be informed of the reason for study termination.

8.3.4.2 Treatment Discontinuation (Stop of Infusion)

The planned duration of drug infusion is described in Section 8.4.1.

Criteria for stop of drug infusion prior to cardioversion or prior to the infusion duration of 30 min are as follows:

- Developing hemodynamic instability (drop in BP, drop in heart rate, unless caused by conversion) including but not limited to
  - systolic BP decrease to <90 mm Hg or significant decrease from baseline (e.g. >40 mm Hg), or mean BP decrease <70 mm Hg (calculated as [systolic BP + 2 × diastolic BP] / 3).
  - systolic BP increase to >190 mm Hg or
  - heart rate <40 bpm or
  - intolerable side effects.
- Developing changes in ECG variables (compared with pre-infusion ECG):
  - QRS +50% and/or development of BBB.
  - QTcF +60 ms.
  - QTcF >500 ms.
- Developing tremors (involuntary, rhythmic muscle contraction leading to shaking movements in one or more parts of the body)
- Per investigator decision, when it is not in the patient's best interest to continue infusion. Specific attention should be paid to any other AEs related to the central nervous system and/or any local infusion site reactions.

Pregnancy

Patients will be instructed that known or suspected pregnancy occurring during the study, in female patients or female partners of male patients, should be confirmed and reported to the investigator. The investigator should also be notified of any pregnancy occurring during the study but confirmed only after completion of the study. In the event that a patient is subsequently found to be pregnant after inclusion in the study, any pregnancy will be followed to term, and the status of mother and child will be reported to Acesion Pharma after delivery.

Full details will be recorded in the eCRF and a pregnancy reporting form will be completed (see Section 10.1.3.1.1).

8.4 Investigational Products

8.4.1 Investigational Products Administered

Study drugs will either be AP30663 or placebo; both will be administered as an intravenous infusion for 30 min.

Prior to the interim analysis (Part 1 of the study), patients randomised to the AP30663 arm will receive AP30663 at a dose of 3 mg/kg.

The starting AP30663 dose(s) for Part 2 of the study will be determined during DMC review of the interim analysis at the end of Part 1. In Part 2, patients randomised to AP30663 may receive one of the following AP30663 doses: 2, 4, 5, or 6 mg/kg dependent on the results seen for the decision criteria, and the subsequent dose recommended by the DMC.

Patients randomised to the placebo arm will receive matching placebo infusion.

The following instructions apply for intravenous infusion of study drugs:

- The patient should be in (semi-) supine position.
- A large peripheral vein (e.g. antecubital) should be chosen.
- For each patient, the largest clinically feasible cannula size should be used at the discretion of the investigator to avoid local injection site reactions.
- After end of infusion, the intravenous lines should be flushed with 5% glucose solution.

Study drugs must be administered as soon as possible after randomisation and preferably not later than 12 h after randomisation.

Specific detailed instructions on study drug administration and a standardised infusion site assessment are provided in the Study Drug Administration Manual.

8.4.2 Identity and Preparation of Investigational Products

AP30663 is a small molecule drug with a molecular weight of [redacted] The drug substance appears as a white to [redacted].

The AP30663 drug product is a concentrate for solution for infusion containing [redacted]. The drug product is provided in a 10 mL glass vial containing 5 mL of AP30663 concentrate. The vial is closed with a coated bromobutyl rubber stopper. The AP30663 drug concentrate is stored in the original container at -15ºC to -25ºC. The AP30663 concentrate for solution for infusion has the following composition (Table 1):

Table 1 Composition of AP30663 Concentrate for Solution for Infusion

Component	Content	Action
AP30663	[redacted]	Active ingredient
[redacted]	[redacted]	pH adjustment
[redacted]	Up to 5 mL corresponds [redacted]	Solvent

Preparation of the final solution for infusion will be done at the clinical site and is described below.

The AP30663 drug concentrate is diluted with sterile 5% glucose solution for infusion to a concentration of 2 mg/mL prior to administration according to the following procedure:

Vials with the AP30663 drug concentrate intended for preparation of the final solution for infusion will be removed from the freezer and placed at room temperature 30 to 60 min before the start of preparation.

The required amount of 5% glucose solution for injection is transferred to an infusion bag. The respective required amount of AP30663 concentrate is added to the infusion bag. The infusion bag is shaken gently during the addition of the concentrate to ensure that a clear solution is obtained. The final solution for infusion must be stored at room temperature and must be used within 24 h after start of preparation.

The required quantity of the final AP30663 solution for infusion is calculated based on the patient’s body weight (determined with clothes but without shoes) rounded to the nearest integer. The following formula will be used for dose calculation:

Body weight [kg] rounded to nearest integer × intended AP30663 dose [mg] ÷ 2 mg/mL

The following rule applies: Body weight decimals 0.1 kg to 0.4 kg will be rounded down and 0.5 to 0.9 kg will be rounded up.

Detailed instructions for the preparation and handling of the final AP30663 solution for infusion including a detailed and easily read checklist and a dose calculation nomogram will be provided in the pharmacy manual. Careful training of unblinded site staff with regards to the reconstitution will be conducted.

Placebo solution is not frozen and will be administered as a 5% glucose solution in the same way as AP30663. To ensure blinding, volume and/or weight of the placebo solution will be adjusted to match that of the AP30663 dose provided. Detailed instructions for the preparation and handling of the placebo solution including a detailed and easily read checklist and a dose calculation nomogram will be provided in the pharmacy manual as well.

Specific procedures to ensure blinding are described in Section 8.4.7.

All investigational products will be manufactured and, if applicable, imported according to the relevant regulatory requirements.

8.4.3 Packaging and Labelling

AP30663 will be manufactured by Onyx/IPCA, Sunderland, United Kingdom. Manufacturing, packaging, and labelling of the AP30663 drug concentrate for infusion will be performed by Recipharm AB, Stockholm, Sweden. All packaging and labelling operations will be performed according to Good Manufacturing Practice for Medicinal Products and the relevant regulatory requirements.

Packaging and labelling of the AP30663 drug concentrate vials will be performed by the central drug depot Almac (location for packaging/labelling/shipping: Craigavon, UK; back-up location for shipping: Dundalk, Ireland). Glucose solution for infusion and empty infusion bags will be centrally purchased and supplied by Almac.

Labelling by Almac will include the following:

1. Labelling of the active vial and its respective carton with a booklet label which also includes kit numbering; frozen conditions are required.

2. Production of another carton (kit) which will contain a 500 mL bag of 5% glucose and an empty infusion bag. The label on the carton and the additional booklet label for the empty infusion bag will have additional text/numbering.

Almac Clinical Services will also provide labels for the infusion bags that will be shipped to the investigational site and labelled by unblinded site staff.

8.4.4 Method of Assigning Patients to Treatment Groups

For Part 1 of the study, eligible patients will be randomised 1:1 (approximately 36 patients randomised to AP30663 or placebo). An additional 18 to 72 patients will be randomised cumulatively in a 2:1 ratio to 1 or more doses of AP30663 (dependent on dose arms open at time of randomisation) or placebo in Part 2 using an interactive web randomisation system (IWRS).

Two separate lists will be utilised for the study, one for Part 1 and a separate one for Part 2. Both lists will be provided by a separate unblinded biostatistical team and provided to the IWRS.

8.4.5 Selection of Doses in the Study

The patients randomised to the active treatment arm in Part 1 of the study will be given an AP30663 dose of 3 mg/kg.

In Part 2, patients randomised to AP30663 may receive one of the following AP30663 doses: 2, 4, 5, or 6 mg/kg. The final decision on AP30663 doses for Part 2 of the study will be taken based on the DMC recommendation based on review of the IAs.

Further details on study design options and associated AP30663 dose levels in Part 2 of the study are described in Section 8.1.

A justification for the selection of AP30663 doses is given in Section 8.2.1.

8.4.6 Selection and Timing of Dose for Each Patient

Patients will be randomly allocated to receive either AP30663 or placebo. Each patient will receive a single intravenous infusion of either AP30663 or placebo on Day 1.

Details of duration of infusion, instructions for infusion, and dose levels before and after the IAs are included in Section 8.4.1.

Calculation of individual doses based on the patient's body weight is described in Section 8.4.2.

8.4.7 Blinding

This is a double-blind study. Acesion Pharma, investigators, and patients will remain blinded to the study treatment allocation until the end of the study. The randomisation list will be kept secure from the study team, investigators, and patients throughout the conduct of the study and until unblinding is authorised by Acesion Pharma and the blinded lead study statistician. The DMC will be unblinded according to the DMC Charter, and a separate unblinded statistical team will be assigned to provide the

analyses to the DMC. Further details of blinded and unblinded personnel will be included in the DMC Charter.

One or more unblinded site staff will be identified at each centre, whose role will be limited to handling the study treatment. Treatment allocation via the IWRS will be provided only to the unblinded site staff and will be sent to the pre-specified fax/email accessible only to unblinded team members.

The unblinded site staff will prepare infusion bags of AP30663 and placebo, label the final infusion bags, and provide them to the investigator in a blinded manner. This procedure is described in the pharmacy manual.

The assessors (e.g. the investigators and site personnel assessing safety and efficacy) as well as the study patients must remain blinded to the treatment assignments throughout the study.

The only planned unblinding other than at the IAs will be at the conclusion of the study, when the database has been cleaned, statistical populations have been defined, and approval for database lock has been given by Acesion Pharma.

The IWRS will be programmed with blind-breaking instructions. The study blind may be broken if, in the opinion of the investigator, it is in the participant's best interest to know the study treatment assignment. Acesion Pharma must be notified before the blind is broken unless identification of the study treatment is required for a medical emergency in which the knowledge of the specific blinded study treatment will affect the immediate management of the participant's condition (e.g. antidote is available). In this case, Acesion Pharma must be notified within 24 h after breaking the blind. The date and reason for breaking the blind must be recorded in the source documentation and eCRF, as applicable.

In case of an SAE that is unexpected and suspected to be causally related to the study drug and that potentially requires expedited reporting to regulatory authorities, the treatment code will be broken for the individual patient by the Safety and Pharmacovigilance Department.

8.4.8 Prior and Concomitant Therapy

Prior therapy with any prescribed/over-the-counter medications within 14 days prior to screening, as well as with all current medications and those initiated after signing of ICF should be recorded as concomitant therapy.

When the patient requires anticoagulation because of an AF episode, anticoagulation should be initiated and maintained according to the current international and national guidelines.

Patients with low potassium or magnesium levels at screening may be appropriately supplemented with potassium or magnesium, respectively, before baseline, according to the local standards. A re-test of the potassium level is required. The patient can be randomised after the potassium has returned to reference range (3.5 to 5.3 mmol/L).

The investigator should consult the medical monitor if there is any uncertainty regarding patient use of a particular drug or drug class.

8.4.8.1 Prohibited Medication and Procedures

Table 2 provides a listing of specific restrictions for concomitant therapy use, with all necessary washout periods described. The table provides examples of prohibited drug categories; however, it is not a comprehensive list of all restricted medications.

If, before the start of study drug infusion, there is a clinical indication in a randomised patient for any therapy that is specifically prohibited, the patient should be discontinued from the study.

Use of prohibited medications and procedures for treatment of AEs in randomised patients after the end of infusion is allowed at the investigator's discretion and is not regarded as a protocol deviation.

The investigator should actively discuss any questions regarding prohibited medications/procedures with the medical monitor before randomising the patient to study treatment or before study treatment initiation.

Table 2 Prohibited Medication

Drug class/<br>specific compound	Examples*<br>(including but not limited to)	Washout period/comments
Antiarrhythmic class I	Examples: ajmaline, disopyramide, flecainide, lidocaine, mexiletine, quinidine, phenytoin, prajmaline, procainamide, propafenone, tocainide	7 days before randomisation and until the completion of the Day 2 visit.
Antiarrhythmic class III	Examples: dofetilide, dronedarone, ibutilide, sotalol,	7 days before randomisation and until the completion of the Day 2 visit.
Amiodarone	Amiodarone	12 weeks before randomisation and until the completion of the Day 2 visit.
CYP3A4 inhibitors	Examples: cannabinoids, clarithromycin, cimethidine, cyclosporine, dexamethasone, diltiazem, disulfiram, doxycycline, fluoxetine, itrakonazole, ketoconazole, loperamide, metronidazole, naloxone, nefazodone, norfloxacilin, sertraline, terfenadine, valproic acid, voriconazole, several antiretroviral drugs	10 days and until the completion of the Day 2 visit.
Loop diuretics	Examples: bumetanide, ethacrynic acid, furosemide, torsemide	Anticipated change in dose or initiation dose is prohibited from screening visit to the end of the infusion
QT-prolonging drugs	Examples: azithromycin, clarithromycin, erythromycin, roxithromycin, fluconazole, ketoconazole, chloroquine, amitriptyline, doxepin, imipramine, haloperidol, risperidone, clozapine, pimozide, droperidol, terfenadine, astemizole, probucol, cisapride	10 days and until the completion of the Day 2 visit.
St John's Wort	-	10 days and until the completion of the Day 2 visit.
Any other investigational drug		3 months before randomisation and during the study
AP30663		Any previous administration

*Please consult the prescription information of a specific drug for more details.

The following procedures are prohibited:

1. Any cardioversion attempt of AF or atrial flutter within 1 week preceding randomisation.
2. Prior failed attempt (no conversion) of pharmacological or DC cardioversion of previous or current AF episode.
3. Significant surgical procedure within 4 weeks preceding the screening visit.
4. Pacemaker or implantable cardioverter defibrillator therapy prior to randomisation and during the treatment phase of the study.
5. Myocardial revascularisation (PCI or CABG) or other revascularisation procedure during the last 6 weeks before randomisation.

8.4.8.2 Rescue Medication

All patients who will not convert from AF during infusion of the study drug or within 90 min after the infusion start should undergo DC electrical cardioversion within 180 min from the time the infusion started. DC electrical cardioversion should be performed using biphasic synchronised cardioverter with energy of 200 J.

All medications necessary for direct-current electrical cardioversion (e.g. sedatives) should be administered per local standards, observing limitations from Section 8.4.8.1 (Prohibited Medication and Procedures), and should be recorded as concomitant medications.

8.4.9 Treatment Compliance

The study drug will only be applied by the investigator on Day 1. The timing and duration of the infusion as well as the dose calculation depending on the patient’s weight will be noted in the patient’s medical file and recorded in the eCRF (day of application). Early termination of infusion and reason for termination will also be recorded in the eCRF.

9 TIMING OF STUDY PROCEDURES

The planned study assessments are listed in Section 8.1.2.

9.1 Screening Visit

Patients will need to provide written informed consent before any study-related procedures are performed.

- Record any AEs that have occurred and any changes in concomitant medication since signing of the informed consent.
- Assess for eligibility (against the inclusion and exclusion criteria).
- Collect full medical and surgical history, including concomitant illnesses/diseases and concomitant medications.
- Record demographic data, such as date of birth or age (according to applicable regulations), ethnic origin, and sex.
- Perform a full physical examination, including body weight and height.
- Record vital signs ([semi]supine BP, heart rate, heart rhythm from ECG).
- Collect blood samples for haematology and clinical chemistry (refer to the Schedule of Assessments in Section 8.1.2 for parameters included), urea (blood urea nitrogen [BUN]), electrolytes (magnesium, sodium, calcium, potassium), International normalised ratio (INR)/activated partial thromboplastin time (APTT), and thyroid stimulating hormone (TSH).

  Note:
  If TSH levels were tested within 1 week before randomisation at a local laboratory certified for the study, those results will be acceptable and should not be re-tested at screening.

  One coagulation parameter (either INR or APTT) is required for an individual patient. The investigator should decide which parameter is relevant for a given patient, taken into consideration clinical presentation and concomitant medications used by that patient. The same coagulation parameter should be consistently analysed throughout the study in an individual patient.
- Perform a 12-lead ECG.
- Perform a transthoracic echo (TTE) if TTE done within 6 months (inclusive) is not available.

9.2 Day 1 – Pre-infusion (Baseline)

The baseline visit on Day 1 with pre-infusion assessments will take place within 7 days of onset of AF. The following procedures will be performed at the baseline visit prior to study drug infusion:

- Reassess for eligibility against the inclusion and exclusion criteria.
- Randomisation[1] will take place for eligible patients, and each will be assigned a patient number.
- Record any AEs that have occurred and any changes in concomitant medication since the previous visit.
- Perform a brief physical examination (general appearance, cardiovascular, respiratory, gastrointestinal, other).
- Record vital signs ([semi]supine BP, heart rate, heart rhythm from ECG).
- Collect blood samples for haematology and clinical chemistry (refer to the Schedule of Assessments in Section 8.1.2 for parameters included) if not already assessed <72 h before baseline.
- Collect blood sample for INR/APTT for all patients on heparin or vitamin K antagonists when screening >24 h before start of infusion.
- Collect baseline PK plasma sample.
- Perform a 12-lead ECG with ECG print-outs within 15 min before infusion start. Confirm diagnosis of AF (defined as absence of discrete P waves and an irregular ventricular rate) based on the 12-lead ECG.
  If QTcF is above 450 ms the infusion should not be initiated.
- Perform 12-lead Holter monitoring (start 30 min before infusion start).
- Perform telemetry (start 30 min before infusion start).

9.3 Day 1 – During Infusion

When all the baseline procedures have been performed and the investigator has confirmed the patient’s eligibility for the study, study drug will be administered as a single intravenous infusion for 30 min on Day 1. The following procedures will be performed during infusion on Day 1:

- Administer study drug as intravenous infusion.

[1] Note, if the patient is found eligible, the randomisation should be planned in such a way that all logistics will occur in the protocol-specified timeframe, including timely reconstitution of study drug, infusion start and – if applicable - DC cardioversion in non-responders within 180 min from infusion start.

- Record any AEs that have occurred since the pre-infusion status and any changes in concomitant medication. Special attention should be paid to monitoring for AEs related to the central nervous system including tremors. Please also refer to section 8.2.4.2 for stop of infusion criteria.
- Record vital signs ([semi]supine BP, heart rate, heart rhythm from ECG).
- Collect PK plasma samples 5 min ± 1 min, 15 min ± 1 min, 25 min ± 1 min, and 30 min - 1 min after start of infusion. The infusion should not be stopped before the 30 min - 1 min PK sample has been collected. In the case of conversion from atrial fibrillation during infusion, an additional PK sample will be taken immediately after conversion.
- Perform a 12-lead digital ECG (with ECG print-outs 10 min after infusion start and, in case of conversion from AF, within 1 min after conversion BUT before infusion stop).
- Continue 12-lead Holter monitoring.
- Continue telemetry.

9.4 Day 1 – Post-Infusion

The following procedures will be performed post-infusion on Day 1:

- Record any AEs that have occurred and any changes in concomitant medication since the infusion. Special attention should be paid to monitoring for AEs related to the central nervous system including tremors.
- Record vital signs ([semi]supine BP, heart rate, heart rhythm from ECG). Should be recorded before an electrical cardioversion, if applicable.
- Collect PK plasma samples at 45 min ± 5 min, 1 h ± 5 min, 1 h 30 min ± 5 min, 4 h ± 5 min, and 8 h ± 5 min after start of infusion.
- Continue 12-lead Holter monitoring until after the 8 h PK sample is collected.
- Perform telemetry until at least 8h after infusion start.
- Do electrical cardioversion in case of AF persistence (refer to the Schedule of Assessments in Section 8.1.2 for exact timing and parameters).

Prior to discharge of the patient, the Day 2 follow-up visit will be scheduled.

9.5 Day 2

The following procedures will be performed on Day 2:

- Record any AEs that have occurred since Day 1 and any changes in concomitant medication. Special attention should be paid to monitoring for AEs related to the central nervous system including tremors.
- Record vital signs (BP, heart rate, heart rhythm from ECG).
- Collect blood samples for clinical chemistry (refer to the Schedule of Assessments in Section 8.1.2 for parameters included).
- Collect PK plasma samples 24 h ± 2 h after start of infusion.
- Perform a 12-lead ECG 24h ± 2 h after start of infusion.

The Day 30 follow-up visit will be scheduled.

9.6 Follow-up/Discontinuation Visit (Day 30)

The follow-up visit will take place 30 days ± 5 days after the previous visit. The following procedures will be performed at the follow-up visit (Day 30):

- Record any AEs that have occurred and any changes in concomitant medication since the previous visit.
- Perform a brief physical examination (general appearance, cardiovascular, respiratory, gastrointestinal, other).
- Record vital signs ([semi]supine BP, heart rate, heart rhythm from ECG).
- Collect blood samples for haematology and clinical chemistry (refer to the Schedule of Assessments in Section 8.1.2 for parameters included), urea (BUN), and INR/APTT.
- Perform a 12-lead ECG.

9.7 Duration of Treatment

Study drug infusion is planned for 30 min for each patient.

Regardless of which study part (Part 1 or 2) the patient participates in, the overall study duration for each patient is up to a maximum of 42 days. The screening and randomisation will occur within 7 days (inclusive) from onset of AF. The duration of treatment and post-treatment follow-up will be 30 days ± 5 days from start of infusion to the follow-up visit.

10 EFFICACY, PHARMACOKINETICS, AND SAFETY VARIABLES

The planned schedule of assessments is shown in Section 8.1.2. The schedule is same for both parts of the study (Parts 1 and 2).

10.1 Efficacy, Pharmacokinetics, and Safety Measurements

10.1.1 Efficacy Variables

10.1.1.1 Holter Recording

ECG assessments for primary efficacy endpoint will be based on Holter ECG. Conversion from AF will be determined by the investigator and documented with a rhythm strip confirming conversion; time of conversion will be recorded in minutes after start of infusion.

In addition, the Holter will record ECG continuously and detect conversion from AF as well as monitor sustainability of the rhythm after conversion. The 12-lead Holter monitoring equipment will be centrally provided by Clario. Analysis of the primary and secondary efficacy endpoints will be based on Holter ECG and ECGs collected by the site. The Holter ECG recording will be initiated 30 min before the planned start of study drug infusion and will be continued for at least 8 h until after the last PK sampling on Day 1. Records will be available for assessment by the DMC at the IAs, will be used for the analysis of the primary and secondary efficacy endpoints, and may be further used for retrospective safety analyses (Section 10.1.3.5).

The following measurements will be provided for all ECGs extracted from the Holter recording: RR, PR, QRS, QT, QTcF, QTcB (optional), heart rate, T- and U- wave morphology classifications.

Triplicate ECGs will be extracted at the same time points as PK sampling except the 24h PK sampling (see Schedule of Assessments in Section 8.1.2) and will be read in a semi-automated manner by a blinded cardiologist. The arithmetic mean of the replicate values was used as the value for that time point.

The ECG laboratory processes all ECGs to produce regulatory-ready research data with all ECG interval duration measurement quality control and quality assurance processes completed on a continuing basis according to ERT’s quality assurance guidelines and standard operating procedures (SOPs).

Central reading in the ECG core laboratory adheres to the following principles:

- The actual times of dosing, extraction windows, and PK sampling are communicated to the central ECG laboratory by the site.

- The central ECG laboratory identifies periods of stable heart rate on the continuous 12-lead ECG tracing within the 3-minute extraction window.
- Review of ECGs from a particular patient is performed by a single reader.
- Baseline and on-treatment ECGs are based on the same lead ECGs from a single patient.
- The primary analysis lead is Lead II. If Lead II is not analysable, then primary lead of analysis is changed to another lead for the entire patient dataset.

Bedside safety ECGs will be collected by the site as described in Section 10.1.3.5.

10.1.2 Pharmacokinetic Variables

10.1.2.1 Plasma Concentrations of AP30663

Venous blood samples for the determination of plasma concentrations of AP30663 will be collected at the times indicated in the Schedule of Assessments in Section 8.1.2.

At each of the 11 (or 12 in case of conversion from AF) sampling time points for PK assessment, 4 mL of venous blood will be collected. The date and the time of each sample collection will be recorded in the eCRF, with the time of study drug injection (or missed injection, if applicable).

Concentration of AP30663 in the plasma will be determined using 2 analyses as outlined in Section 11.1.5 where concentration-derived PK parameters are also described.

10.1.3 Safety Assessments

10.1.3.1 Adverse Events

Adverse Event Definition

An AE is defined as any untoward medical occurrence in a clinical study patient administered a medicinal product which does not necessarily have a causal relationship with this treatment. An AE can therefore be any unfavourable and unintended sign (including an abnormal laboratory finding), symptom, or disease temporally associated with the use of a medicinal (investigational) product, whether or not it is related to the medicinal (investigational) product. This includes an exacerbation of pre-existing conditions or events, intercurrent illnesses, drug interaction or the significant worsening of the indication under investigation that is not recorded elsewhere in the eCRF under specific efficacy assessments. Anticipated fluctuations of pre-existing conditions, including the disease under study, that do not represent a clinically significant exacerbation or worsening need not be considered AEs.

It is the responsibility of the investigator to document all AEs that occur during the study. AEs will be collected from study assessments including laboratory parameters, physical examination and vital signs and also be elicited by asking the patient a non-leading question, for example, “Have you experienced any new or changed symptoms since we last asked/since your last visit?” AEs should be reported on the appropriate pages of the eCRF.

When collecting AEs, special attention should be paid to AEs related to the risks described in the investigator’s brochure section 7 “Summary of Data and Guidance for the Investigator”.

Assessment of Severity

Each AE will be assigned a category by the investigator as follows:

Mild:	An AE that is easily tolerated by the patient, causes minimal discomfort, and does not interfere with everyday activities.
Moderate:	An AE that is sufficiently discomforting to interfere with normal everyday activities; intervention may be needed.
Severe:	An AE that prevents normal everyday activities; treatment or other intervention usually needed.

If there is a change in severity of an AE, it must be recorded as a separate event.

All local infusion site reactions will be regarded as AEs and reported accordingly. Specific detailed instruction on a standardised assessment of infusion site AEs is provided in the Study Drug Administration Manual.

Assessment of Causality

Every effort will be made by the investigator to assess the relationship of the AE, if any, to the study drug. Causality should be assessed using the categories presented in the following table:

Unrelated:	Clinical event with an incompatible time relationship to study drug administration, and that could be explained by underlying disease or other drugs or chemicals or is incontrovertibly not related to the study drug.
Unlikely:	Clinical event whose time relationship to study drug administration makes a causal connection improbable, but that could plausibly be explained by underlying disease or other drugs or chemicals.
Possible:	Clinical event with a reasonable time relationship to study drug administration, but that could also be explained by concurrent disease or other drugs or chemicals.

Probable:	Clinical event with a reasonable time relationship to study drug administration, and is unlikely to be attributed to concurrent disease or other drugs or chemicals.
Very Likely/Certain:	Clinical event with plausible time relationship to study drug administration, and that cannot be explained by concurrent disease or other drugs or chemicals.

Action Taken

The investigator will describe the action taken in the appropriate section of the eCRF, as follows:

- None
- Study drug stopped
- Study drug temporarily interrupted
- Concomitant medication
- Other, specify.

Follow-up of Adverse Events

All investigators should follow patients with AEs until the event is resolved or until, in the opinion of the investigator, the event is stabilised or determined to be chronic. Details of AE resolution must be documented in the eCRF.

Patients should be followed for 30 days after receiving the study drug, and any AEs that occur during this time should be reported according to the procedures outlined below.

Documentation and Reporting of Adverse Events

AEs should be reported and documented in accordance with the procedures outlined below. All AEs occurring during the study must be documented in the relevant eCRF pages. The following data should be documented for each AE:

- Description of the event
- Classification of “serious” or “not serious”
- Severity
- Date of first occurrence and date of resolution (if applicable)
- Action taken
- Causal relationship

- Outcome of event (unknown, recovered, not yet recovered, recovered with sequelae, death [with date and cause reported])

10.1.3.1.1 Serious Adverse Events

Serious Adverse Event Definition

An SAE is any untoward medical occurrence or effect that, at any dose,

- Results in death.
- Is life-threatening. (An AE is life-threatening if the patient was at immediate risk of death from the event as it occurred, i.e. it does not include a reaction that might have caused death if it had occurred in a more serious form.)
- Requires or prolongs inpatient hospitalisation. (Complications occurring during hospitalisation are AEs and are SAEs if they cause prolongation of the current hospitalisation. Hospitalisation for elective treatment of a pre-existing non-worsening condition is not, however, considered an AE. The details of such hospitalisations must be recorded on the medical history or physical examination page of the eCRF.)
- Results in persistent or significant disability/incapacity. (An AE is incapacitating or disabling if it results in a substantial and/or permanent disruption of the patient's ability to carry out normal life functions.)
- Results in a congenital anomaly/birth defect.

In addition, medical and scientific judgement is required to decide if prompt notification is required in situations other than those defined for SAEs above. This may include any event that the investigator regards as serious that did not strictly meet the criteria above but may have jeopardised the patient or required intervention to prevent one of the outcomes listed above, or that would suggest any significant hazard, contraindication, side effect, or precaution that may be associated with the use of the investigational product.

Note that if DC cardioversion is performed as indicated in the schedule of assessments (Section 8.1.2), it should not be reported as AE/SAE.

Reporting of Serious Adverse Events

Any SAE must be reported by the investigator if it occurs during the clinical study from signing of the ICF or within 30 days of receiving the study drug, whether or not the SAE is considered to be related to the investigational product. An SAE report consists of the SAE form. A copy of these forms must be faxed **within 24 h** for the attention of the product safety scientist at

Syneos Health Safety and Pharmacovigilance Department
Fax: +1-877-464-7787 or
Email: SafetyReporting@SyneosHealth.com

The investigator should not wait to receive additional information to document the event fully before notification of an SAE, though additional information may be requested. Where applicable, information from relevant laboratory results, hospital case records, and autopsy reports should be obtained.

Instances of death, congenital abnormality, or an event that is of such clinical concern as to influence the overall assessment of safety, if brought to the attention of the investigator at any time after cessation of study drug administration and linked by the investigator to this study, should be reported to the study monitor.

Acesion Pharma and/or Syneos Health will promptly notify all relevant investigators and the regulatory authorities of findings that could adversely affect the safety of patients, impact on the conduct of the study, or alter the independent ethics committee (IEC) approval/favourable opinion of the study. In addition, Syneos Health, on behalf of Acesion Pharma, will expedite the reporting to all concerned investigators, to the IECs, where required, and to the regulatory authorities of all adverse reactions that are both serious and unexpected.

Reporting of Pregnancies

All pregnancies that occur in a patient or patient’s partner during the study will be reported using a separate pregnancy reporting form. Reporting follows the same process as described above for SAE reporting. If there is also an SAE reported or suspected, an SAE form should also be completed and sent to the Syneos Health Safety and Pharmacovigilance Department as described above.

Further details of the procedures to be followed if a pregnancy occurs are provided in Section 8.3.4.2.

10.1.3.1.2 Unexpected Adverse Reactions

Unexpected Adverse Reaction Definition

An unexpected adverse reaction is any untoward and unintended response that is related to the administration of the study drug at any dose that is not consistent with the applicable product information (e.g. investigator’s brochure for an unauthorised investigational medicinal product or summary of product characteristics for an authorised product).

All suspected unexpected serious adverse reactions (SUSARs) will be the subject of expedited reporting. Only SUSARs assessed to be possibly, probably, or very

likely/certainly related to study drug are considered to have a reasonable causal relationship to study drug and are subject to reporting to regulatory authorities.

Acesion Pharma and/or Syneos Health shall ensure that all relevant information about a SUSAR that is fatal or life-threatening is reported to the relevant competent authorities and IEC within 7 days after knowledge by Acesion Pharma of such a case and that relevant follow-up information is communicated within an additional 8 days. All other SUSARs will be reported to the relevant competent authorities and IEC within 15 days after knowledge by Acesion Pharma of such a case. All investigators should follow SUSARs until the event is resolved or until, in the opinion of the investigator, the event is stabilised or determined to be chronic. Post-study SUSARs that occur after the patient has completed the clinical study must be reported by the investigator to Acesion Pharma.

Warnings and Precautions

There are no specific warnings associated with AP30663 administration.

Precautions to avoid local infusion site reactions are described in Section 8.4.1.

Please refer to the most current version of the investigator's brochure for warnings and precautions.

10.1.3.2 Clinical Laboratory Evaluation

The haematology and clinical chemistry laboratory analyses will be performed at local laboratories. Reference ranges will be supplied by local laboratories and used by the investigator to assess the laboratory data for clinical significance and pathological changes. The following laboratory safety tests will be performed at the times outlined in the Schedule of Assessments in Section 8.1.2.

The total amount of blood to be taken during the study from screening until the end of the 30-day study period amounts to a maximum of 131 mL (including blood samples for PK analyses). See Section 17.2 for an overview of blood samples and volumes collected.

Haematology

Haemoglobin, haematocrit, red blood cells (erythrocytes), white blood cells (leukocytes, total and differential), basophils, eosinophils, lymphocytes, monocytes, neutrophils, and platelets.

Clinical Chemistry

ALT, AST, bilirubin, creatinine, gamma-glutamyltransferase, glucose (random), eGFR, urea (BUN), sodium, potassium, calcium, and magnesium.

Other Laboratory Variables

INR/APTT and TSH

10.1.3.3 Vital Signs

Vital signs will be recorded as indicated in the Schedule of Assessments in Section 8.1.2. They will include BP, heart rate, and heart rhythm (from ECG) and will be recorded in a standardised manner, i.e. after the patient has rested in the (semi)supine position for 5 min and before blood sampling or laboratory tests. Vital signs measurements will be repeated in case of clinically significant abnormalities.

10.1.3.4 Physical Examination and Brief Physical Examination

At screening, weight and height will be recorded and a physical examination will be performed, followed by a brief physical examination at baseline (pre-infusion on Day 1) and Day 30. Any changes from the screening and baseline visit to Day 30 will be recorded. Full and brief physical examination will include the following assessments:

Table 3 Physical Examination

Assessment	Full physical examination	Brief physical examination
General appearance	X	x
Head, ears, eyes, nose, throat	X	-
Cardiovascular	X	x
Respiratory	X	x
Gastrointestinal	X	x
Dermatological	X	-
Neurological	X	-
Musculoskeletal	X	-
Lymphatic	X	-
Other (at the investigator's discretion)	X	x

10.1.3.5 Electrocardiogram

10.1.3.5.1 12-lead Digital Electrocardiogram

A 12-lead digital ECG will be performed at the times outlined in the Schedule of Assessments in Section 8.1.2 in a standardised manner (i.e. after the patient has rested in the [semi]supine position for at least 5 min).

The investigator will assess ECG abnormalities (e.g. ischaemia, conduction abnormalities, and rhythm abnormalities) and define the ECG intervals as part of the

screening of eligibility and according to the inclusion/exclusion criteria, and as part of an adverse event assessment.

The following measurements will be provided for all PK time points: RR, PR, QRS, QT, QTcF (Fridericia's correction of QT interval), QTcB (optional), heart rate, as well as T- and U-wave morphology classifications.

Bedside safety ECGs will be collected by the site, printed and reviewed by the investigator on site.

ECG intervals based on these safety ECGs will only be recorded if part of an adverse event.

10.1.3.5.2 Telemetry

Heart rhythm will be recorded by telemetric monitoring as outlined in the Schedule of Assessments in Section 8.1.2. Lead positioning on the patient for telemetric monitoring will be performed per local standards and local requirements of the telemetry equipment. During the 8 h post-infusion of study drug, the patient will remain under continuous medical observation. Should any significant heart rhythm changes occur during this observation, the recording from telemetry should be printed out, assessed for clinical significance, and recorded in the eCRF.

10.1.3.5.3 Holter Recording

A Holter recording will be performed the first 8 hours after infusion start for efficacy assessments and evaluated centrally as outlined in Section 10.1.1.1. Sites will be contacted by email or telephone if clinical alerts have been detected on the extracted ECG or Holter reports overread by the central ECG laboratory.

Holter data will be retained in case additional retrospective analysis should be necessary for safety.

10.2 Independent Data Monitoring Committee

Safety, tolerability, PK/PD and efficacy will be regularly reviewed by an unblinded independent DMC to advise on dose escalation, de-escalation, and study progression decisions at the interim analyses. The DMC will also periodically review accumulating safety, tolerability, efficacy and PK/PD data throughout the study.

At the end of Part 1, after up to 36 initial patients receiving AP30663 and placebo have completed Day 2 assessments of the study (or terminated the study), the DMC will be convened to review the accumulating unblinded efficacy (including ECG and Holter ECG), PK/PD, safety and tolerability data for the study in an interim analysis, and provide a recommendation on dose escalation, de-escalation, and study progression/early termination in case there is a concern regarding safety (see Section 8.1

for options regarding Part 2 study design). A pre-determined efficacy algorithm will be put in place to guide the independent DMC in its decision. Decision rules will be based on the AF conversion rate, safety and tolerability signals including but not limited to increase in QTcF interval and number and severity of local injection site reactions.

During the enrolment period, an IA may be planned on a reduced cohort if preliminary blinded safety and efficacy data indicate that current enrolment is sufficient for the DMC to provide a recommendation for Part 2.

Full details of composition, operational aspects, and data to be reviewed and recommendations to be made by the DMC will be described in the DMC Charter. Detailed decision rules will be outlined in the DMC Charter or the SAP.

10.3 Appropriateness of Measurements

The efficacy and safety assessments planned for this study are widely used and generally recognised as reliable, accurate, and relevant to the disease condition.

11 STATISTICAL METHODS

11.1 Statistical and Analytical Plans

The statistical analysis will be performed using SAS® version 9.4 or higher. The main population for efficacy analysis will be the Full Analysis Set; supportive analyses will also be performed using the Per-Protocol Set.

Continuous data will be presented using descriptive summaries (e.g. mean, standard deviation [SD], minimum, maximum, median, lower quartile, and upper quartile). Categorical variables will be presented by the number of observations and relative (%) frequency.

Unless otherwise stated, the baseline value for any variable will be the last non-missing value taken prior to the infusion of the double-blind study treatment on Day 1. Pre-infusion Day 1 assessments will be included in this derivation of baseline.

Unless otherwise stated, all statistical tests will be 2-sided and conducted at the 5% level. All presented confidence intervals will be 2-sided 95% confidence intervals.

The SAP will be developed and finalised before database lock and will describe the selection of participants to be included in the analysis and procedures for accounting for unused and spurious data.

11.1.1 Datasets or Populations Analysed

For the purposes of analysis, the following populations are defined for both Parts 1 and 2. Any decisions required for inclusion/exclusion of a population (for example, Per-Protocol Set) will be made for all patients across both Parts 1 and 2.

Population	Description
Enrolled Set	The Enrolled Set will include all participants who sign the ICF.
Randomised Set	The Randomised Set will include all participants who signed the ICF and were subsequently randomised into the study, regardless of study treatment administration.
Full Analysis Set	The Full Analysis Set will serve as the primary population for the analysis of efficacy and will consist of all randomised participants who were administered double-blind study treatment and have an evaluable AF conversion status within 90 min from the start of infusion. Participants will be analysed according to the randomised treatment.

Population	Description
Per-Protocol Set	The Per-Protocol Set includes all participants from the Full Analysis Set who have been treated according to the protocol and fulfil the following criteria (to be further described in the SAP):<br>1. All inclusion/exclusion criteria satisfied<br>2. Absence of relevant protocol violations with respect to factors likely to affect the efficacy of treatment where the nature of protocol violation will be defined before breaking the blind<br>3. Adequate study drug compliance, which will be determined before breaking the blind<br>Participants will be analysed according to the randomised treatment.
Safety Set	All randomised participants who were administered double-blind study treatment. Participants will be analysed according to the treatment received.
PK Set	The PK Set will include all participants in the Safety Set who have at least one evaluable post-baseline drug concentration value.

Abbreviations: AF = atrial fibrillation, ICF = informed consent form, PK = pharmacokinetic, SAP = statistical analysis plan.

11.1.2 Demographic and Other Baseline Characteristics

Descriptive statistical methods will be used to tabulate and summarise demographics and baseline characteristics.

11.1.3 Efficacy Variables

Definition of Primary Efficacy Endpoint

The primary efficacy endpoint is defined as the proportion of patients who have converted from AF within 90 min from the start of infusion, and subsequently have no AF recurrence within 1 min of conversion from AF.

Definition of Secondary Efficacy Endpoints

- The time to conversion from AF from start of infusion.
- The proportion of patients with relapse of AF within 5 min (IRAF) after pharmacological or DC cardioversion.
- The proportion of patients in SR at 3 h ± 1 h after start of infusion.
- The proportion of patients in SR at 24 h ± 2 h after start of infusion.
- The proportion of patients in SR at 30 days ± 5 days after start of infusion.

Methods of Analysis

For the analysis of the primary endpoint (proportion of patients who have cardioversion within 90 min from the start of infusion) a Bayesian model will be utilised. The prior probability of success at a dose, d, is modelled with a P_d ~ Beta (1,1) prior (uniform) for each dose. Across both Parts 1 and 2 of the study, the potential doses (arms) in the trial are d = 0 (placebo), 2, 3, 4, 5, and 6 (all mg/kg dose units).

Let X_d be the number of successes and N_d be the number of observations at dose d. The posterior distribution is calculated for each dose independently using a Beta posterior distribution,

$$P_d \sim \text{Beta}\ (1 + X_d,\ 1 + N_d - X_d).$$

The adaptive design utilises the probability that the response rate at a dose is greater than a 65% success rate. This is ($P_d > 0.65$), for each dose d.

This modelling will occur for each interim analysis, and at the final analysis as primary method. At the conclusion of the study, each AP30663 dose will be considered to be superior to placebo, if the posterior probability of AP30663 dose has a higher success rate than placebo is greater than 0.95.

Further to the above, for the final analysis the primary endpoint will also be analysed by means of a logistic regression model. Relevant covariates at baseline may be added into the model after reviewing baseline imbalances.

Secondary endpoints relating to proportions will be analysed using a logistic regression model in a similar manner to that of the primary endpoint. Time to conversion will be analysed using Kaplan-Meier methods and, if met, the median time to conversion will be presented, along with the corresponding Kaplan-Meier plot stratified by treatment group.

Full details of all statistical analyses will be provided in the SAP.

11.1.4 Safety Variables

The safety endpoints are as follows:

- AEs, ECG variables including significant arrhythmia, physical examination, vital signs, and laboratory variables.
- Changes in QTcF interval data over time.

Methods of Analysis

AEs will be coded using the Medical Dictionary for Regulatory Activities (MedDRA). Summaries will be presented by system organ class (SOC) and preferred term. Treatment-emergent AEs (TEAEs) are defined as any AE occurring or worsening on or

after the first dose of study medication. If a participant experiences the same preferred term multiple times, the event will be counted only once overall and by the greatest severity.

The frequency and incidence of TEAEs will be presented by SOC and preferred term for each treatment group (number and percentage of patients experiencing at least one TEAE per preferred term as well as the number of observed events per preferred term). Separate tables will be presented by severity and by relationship, and for SAEs, related TEAEs, and TEAEs leading to study discontinuation. All AEs will be presented in full in a comprehensive listing including participant number, treatment regimen, severity, seriousness, action taken, outcome, relationship to treatment, onset/stop, and duration. Details of SAEs and AEs leading to withdrawal will be listed separately.

For ECG values, a categorical analysis of outliers (e.g. number of ECGs where QTc >450 ms, and QTc >500 ms and change from baseline of >30 ms and >60 ms) will be computed and summarised.

Changes in QTcF intervals over time will be summarised by means of a shift table. Abnormal findings ("normal", "abnormal, not clinically significant", and "abnormal, clinically significant") will be summarised by the number and percentage within each category, and change from baseline will be summarised within shift tables.

11.1.5 PK Variables

The PK endpoints are as follows:

- Systemic exposure derived from the population PK model.
- Population PK model parameter estimates derived from plasma concentrations of AP30663.

Methods of Analysis

The concentration at each time point will be summarised as arithmetic mean, SD, median, minimum, and maximum.

Descriptive statistics (n, arithmetic mean, SD, minimum, median, maximum) by treatment group will be calculated for all derived PK endpoints.

Concentration of AP30663 in the plasma will be determined using 2 analyses.

- A static analysis based on plasma concentration will be performed using a non-compartmental analysis. Data from patients with missing concentration values (missing samples) may be used if PK parameters can be estimated using the remaining data points.

- In addition, PK parameters will be derived using a non-linear mixed-effect modelling approach with NONMEM. A pre-specified population PK model based on existing information from previous Phase 1 study (structural model) will be updated with the AP30663 plasma concentrations obtained in this study.

For the non-compartmental analysis, the following PK parameters will be determined for each patient based on plasma concentrations:

- $AUC_{0\text{-}0.5}$: Area under the concentration time curve from pre-dose concentration up to 30 min. $AUC_{0\text{-}0.5}$ includes the plasma drug concentration up to the end of infusion
- $AUC_{0\text{-}t}$: Area under the concentration time curve up to the last measurable concentration
- $AUC_{0\text{-}inf}$: Area under the concentration time curve from pre-dose through concentration to infinity (extrapolated), calculated as $AUC_{0\text{-}t}$ + Ct/K_{el}, where Ct is the last observed non-zero concentration
- C_{max}: The observed peak plasma drug concentration
- T_{max}: The time at which C_{max} occurs
- $t_{1/2}$: the terminal half-life value will be calculated using the equation ln2/ K_{el}, with K_{el} being the elimination rate constant
- K_{el}: Elimination rate constant. This parameter will be the negative of the estimated slope of the linear regression of the ln-transformed concentration versus time profile in the terminal elimination phase. At least 3 concentration points will be used in estimating K_{el}. The time point where ln-linear K_{el} calculation begins (K_{el} Lower), and the actual sampling time of the last quantifiable concentration used to estimate the K_{el} (K_{el} Upper) will be reported with the correlation coefficient from the linear regression to calculate K_{el}

From the developed population PK model, PK parameters like volume of distribution and clearance will be estimated. Additional PK parameters including but not limited to area under the curve at steady-state will be derived from the individual Bayes estimates from the population PK model.

Software

Population PK analyses will be performed using NONMEM® version 7.4. Analysis datasets and graphical analyses other than non-linear mixed-effect modelling will be performed using R v3.5.0.

Population PK Approach

The population PK model will be developed using the analysis dataset and guided by the results of exploratory analysis. During the model development process, the structural, statistical, and covariate model will be selected. The model developed will be internally qualified using several diagnostics and statistical techniques as non-parametric bootstrap. A complete description of the population PK approach will be presented in a separate PK modelling analysis plan.

11.1.6 Interim Analyses

The study is conducted in 2 parts (Part 1 and 2). For all IAs during the study, a separate unblinded biostatistical team will prepare and present the unblinded analysis, and the DMC will be convened to review the data and will give their recommendations pertaining to continuation or early termination of the study, and as well as whether or not to increase/decrease the dose per the algorithm below and/or any other available data.

The first IA will be conducted after all randomised patients have completed Day 2 assessments or terminated the study during Part 1. The DMC will be convened to review unblinded data on the primary efficacy endpoint, PK/PD, safety and tolerability data during this IA.

If the DMC decides that the study should continue, then Part 2 of the study will be enacted. During Part 2, IAs will be conducted when 18, 36, and 54 randomised patients have completed Day 2 assessments or terminated the study during Part 2.

For each IA, the success rate for any single dose level d, P_d, will be analysed using a Bayesian model as described in Section 11.1.3, and the following decision criteria will be implemented, dependent on the IA to be reviewed:

Interim Analysis at End of Part 1:

During the first IA the following will occur, regardless of the response seen for the AP30663 3 mg/kg dose:

- Dose 3 (3 mg/kg AP30663) will be stopped.
- Dose 5 (5 mg/kg AP30663 versus placebo) will be opened.

Additionally, the following decision will be made dependent on the response rate seen for 3 mg/kg AP30663:

- If the proportion of patients achieving a response under AP30663 3 mg/kg is > 0.65 then Dose 2 (2 mg/kg AP30663) will additionally be opened for Part 2 (in parallel to Dose 5).

Interim Analyses During Part 2:

Interim analyses will occur when 18, 36, and 54 randomised patients have completed Day 2 assessments or terminated the study in Part 2. At these IAs the following rules will be utilised:

a) If an open Dose d has at least 10 randomised patients having completed Day 2 assessments and satisfies: $(P_d > 0.65) \geq 0.90$ then Dose d will be closed for sufficient efficacy response.

- If the dose 1one level below this dose has never been opened, then dose d-1 is opened.

b) If an open Dose d has at least 10 randomised patients having completed Day 2 assessments and satisfies $(P_d > 0.65) < 0.10$ then Dose d will be closed.

- If Dose d was the largest dose thus far open, and Dose d is < 6 mg/kg, then dose d + 1 will be opened.

The study will continue until all doses of AP30663 have been closed or the maximum of 72 patients during Part 2 (up to 108 patients total across Part 1 and Part 2) have been enrolled.

While the algorithm, and the DMC's decision for dose increase/decrease, is based on AF conversion rate only, the DMC will also convene to review safety and tolerability data at defined time points; review of this data may also lead to further decisions being made by the DMC.

Further details of the IAs and the decision rules to be implemented will be detailed in the SAP and the DMC Charter.

11.1.7 Handling of Missing Data

No imputation of missing data will be performed.

11.2 Determination of Sample Size

The study is proof of concept, designed to allow for a considered testing of a range of doses of AP30663, compared with placebo. Based on the study design, the sample size for randomisation will range from up to 36 patients (for Part 1) to up to 108 patients (for Parts 1 and 2 cumulatively). The total sample size is dependent on the AP30663 dose arms opened on the decision of the DMC. A total of 4 IAs and a final analysis are included in the design, and so it is foreseen that the total number of randomised patients will be up to 36, 54, 72, 90, or 108 patients to have a sufficient number of patients evaluable for AF conversion at each IA.

The adaptive design utilises the probability that the response rate at a Dose d is greater than a success rate of 65% ($P_d > 0.65$, where d is the dose arm of AP30663), based on a Bayesian modelling procedure as stated in Section 11.1.3.

11.3 Protocol Deviations

The investigator should not implement any deviation from the protocol without prior review and agreement by Acesion Pharma and in accordance with the IECs and local regulations, except when necessary to eliminate an immediate hazard to study patients. Such contact must be made as soon as possible to permit a review by the investigator/ Acesion Pharma to determine the impact of the deviation on the patient and/or the study. Any significant protocol deviations affecting patient eligibility and/or safety must be reviewed and/or approved by the IEC, as applicable, before implementation. Protocol deviations will be listed in the clinical study report.

12 QUALITY ASSURANCE AND QUALITY CONTROL

12.1 Audit and Inspection

Study centres and study documentation may be subject to quality assurance audit during the course of the study by Acesion Pharma or its nominated representative. In addition, inspections may be conducted by regulatory authorities at their discretion.

12.2 Monitoring

Data for each patient will be recorded in an eCRF. Data collection must be completed for each patient who signs an ICF and is administered study drug.

In accordance with current Good Clinical Practice and International Council for Harmonisation (ICH) guidelines, the study monitor will carry out source document verification at regular intervals to ensure that the data collected in the eCRF are accurate and reliable.

The investigator must permit the monitor, the IEC, Acesion Pharma’s internal auditors, and representatives from regulatory authorities direct access to all study-related documents and pertinent hospital or medical records for confirmation of data contained within the eCRFs.

12.3 Data Management and Coding

Syneos Health will be responsible for activities associated with the data management of this study. This will include setting up a relevant database and data transfer mechanisms, along with appropriate validation of data and resolution of queries. Data generated within this clinical study will be handled according to the relevant SOPs of the data management and biostatistics departments of Syneos Health.

Study centres will enter data directly into an electronic data capture (EDC) system by completing the eCRF via a secure internet connection. Data entered in the eCRF must be verifiable against source documents at the study centre. Data to be recorded directly in the eCRF will be identified and the eCRF will be considered the source document. Any changes to the data entered into the EDC system will be recorded in the audit trail and will be FDA CFR 21 Part 11 compliant.

Medical coding will use MedDRA for concomitant diseases and AEs and WHODrug for medications.

Missing or inconsistent data will be queried in writing to the investigator for clarification. Subsequent modifications to the database will be documented.

12.4 Quality Management and Risk Evaluation

Details are provided in Section 8.2.2.

13 RECORDS AND SUPPLIES

13.1 Drug Accountability

On receipt of the study drug (including rescue medication, if relevant), the delegated unblinded site staff will conduct an inventory of the supplies and verify that study drug supplies are received intact and in the correct amounts before completing a supplies receipt. The unblinded monitor may check the clinical study supplies at each study centre at any time during the study.

It is the responsibility of the unblinded study monitor to ensure that the delegated unblinded site staff have correctly documented the amount of the study drug received, dispensed, and returned on the dispensing log that will be provided. A full drug accountability log will be maintained at the study centre at all times. The unblinded study monitor will arrange collection of unused study drug. The unblinded study monitor will also perform an inventory of study drug before or during the close-out visit to the study centre. All discrepancies must be accounted for and documented.

Drug accountability will be performed by the unblinded site staff and verified by the unblinded study monitor. Responsibilities of the unblinded site staff and the unblinded study monitor will not overlap with responsibilities of the blinded site staff and the blinded study monitor.

13.2 Financing and Insurance

Financing and insurance of this study will be outlined in a separate agreement between Syneos Health and Acesion Pharma.

14 ETHICS

14.1 Independent Ethics Committee

Before initiation of the study at each study centre, the protocol, the ICF, other written material given to the patients, and any other relevant study documentation will be submitted to the appropriate IEC. Written approval of the study and all relevant study information must be obtained before the study centre can be initiated or the study drug is released to the investigator. Any necessary extensions or renewals of IEC approval must be obtained for changes to the study such as amendments to the protocol, the ICF or other study documentation. The written approval of the IEC with the approved ICF must be filed in the study files.

The investigator will report promptly to the IEC any new information that may adversely affect the safety of the patients or the conduct of the study. The investigator will submit written summaries of the study status to the IEC as required. On completion of the study, the IEC will be notified that the study has ended.

14.2 Regulatory Authorities

Relevant study documentation will be submitted to the regulatory authorities of the participating countries, according to local/national requirements, for review and approval before the beginning of the study. On completion of the study, the regulatory authorities will be notified that the study has ended.

14.3 Ethical Conduct of the Study

The investigator(s) and all parties involved in this study should conduct the study in adherence to the ethical principles based on the Declaration of Helsinki, GCP, ICH guidelines, and the applicable national and local laws and regulatory requirements.

14.4 Informed Consent

The process of obtaining informed consent must be in accordance with applicable regulatory requirement(s) and must adhere to GCP.

The investigator is responsible for ensuring that no patient undergoes any study-related examination or activity before that patient has given written informed consent to participate in the study.

The investigator or designated personnel will inform the patient of the objectives, methods, anticipated benefits, and potential risks and inconveniences of the study. The patient should be given every opportunity to ask for clarification of any points she/he does not understand and, if necessary, ask for more information. At the end of the interview, the patient will be given ample time to consider the study. Patients will be required to sign and date the ICF. After signatures are obtained, the ICF will be kept and

archived by the investigator in the investigator's study file. A signed and dated copy of the patient ICF will be provided to the patient or their authorised representative.

It should be emphasized that the patient may refuse to enter the study or to withdraw from the study at any time, without consequences for her/his further care or penalty or loss of benefits to which the patient is otherwise entitled. Patients who refuse to give or who withdraw written informed consent should not be included in or continue in the study.

If new information becomes available that may be relevant to the patient's willingness to continue participation in the study, a new ICF will be approved by the IEC(s) (and regulatory authorities, if required). The study patients will be informed about this new information and reconsent will be obtained.

14.5 Patient Confidentiality

Monitors, auditors, and other authorised agents of Acesion Pharma and/or its designee, the IEC(s) approving this research, as well as that of any other applicable agency(ies), such as the European Medicines Agency (EMA), will be granted direct access to the study patients' original medical records for verification of clinical study procedures and/or data, without violating the confidentiality of the patients to the extent permitted by the law and regulations. In any presentations of the results of this study or in publications, the patients' identity will remain confidential.

15 REPORTING AND PUBLICATION, INCLUDING ARCHIVING

Essential documents are those documents that individually and collectively permit evaluation of the study and quality of the data produced. After completion of the study (end of study defined as the date of the last visit of the last patient), all documents and data relating to the study will be kept in an orderly manner by the investigator in a secure study file. This file will be available for inspection by Acesion Pharma or its representatives. Essential documents should be retained for (whichever takes longer) 25 years after the completion of the study, for 2 years after the final marketing approval in an ICH region, or for at least 2 years since the discontinuation of clinical development of the investigational product. It is the responsibility of Acesion Pharma to inform the study centre when these documents no longer need to be retained. The investigator must contact Acesion Pharma before destroying any study-related documentation. In addition, all patient medical records and other source documentation will be kept for the maximum time permitted by the hospital, institution, or medical practice.

Acesion Pharma must review and approve any results of the study or abstracts for professional meetings prepared by the investigator(s). Published data must not compromise the objectives of the study. Data from individual study centres in multicentre studies must not be published separately.

16 REFERENCES

Feinberg WM, Blackshear JL, Laupacis A, Kronmal R, Hart RG. Prevalence, age distribution, and gender of patients with atrial fibrillation. Analysis and implications. *Arch Intern Med.* 1995;155(5):469-473.

Kirchhof P, Benussi S, Kotecha D, et al. 2016 ESC Guidelines for the management of atrial fibrillation developed in collaboration with EACTS. *Europace.* 2016;18(11):1609-1678.

Zoni-Berisso M, Lercari F, Carazza T, Domenicucci S. Epidemiology of atrial fibrillation: European perspective. *Clin Epidemiol.* 2014;6:213-220.

17 APPENDICES

17.1 Investigator Signature Page

Protocol Title: A Double-Blind, Randomised, Placebo-Controlled, Parallel-Group Study of AP30663 Given Intravenously for Cardioversion in Patients with Atrial Fibrillation

Protocol Number: AP30663 - 2001

Confidentiality and Current Good Clinical Practice Compliance Statement

I, the undersigned, have reviewed this protocol (and amendments), including appendices, and I will conduct the study as described in compliance with this protocol (and amendments), GCP, and relevant ICH guidelines.

Once the protocol has been approved by the IEC, I will not modify this protocol without obtaining prior approval of Acesion Pharma and of the IEC. I will submit the protocol amendments and/or any ICF modifications to Acesion Pharma and IEC, and approval will be obtained before any amendments are implemented.

I understand that all information obtained during the conduct of the study with regard to the patients' state of health will be regarded as confidential. No patients' names will be disclosed. All patients will be identified by assigned numbers on all eCRFs, laboratory samples, or source documents forwarded to Acesion Pharma. Clinical information may be reviewed by Acesion Pharma or its agents or regulatory agencies. Agreement must be obtained from the patient before disclosure of patient information to a third party.

Information developed in this clinical study may be disclosed by Acesion Pharma, to other clinical investigators, regulatory agencies, or other health authority or government agencies as required.

____________________________________ ____________________
Investigator Signature Date

____
Printed Name

____
Institution

17.2 Blood Sampling

Laboratory test	Maximum number of samples collected	Maximum volume per test	Total volume
Haematology	3	3 - 4 mL	9 - 12 mL
Clinical chemistry	3 - 4	8.5 - 10 or 14 mL[a]	25.5 - 56 mL
Blood ions	1 - 2	included in chemistry	included in chemistry
Coagulation (INR/APTT)	2 - 3	2.7 - 5 mL	5.4 – 15 mL
TSH	1	included in chemistry	included in chemistry
PK	11 - 12	2 - 4 mL	22 - 48 mL

Abbreviations: ALT = alanine aminotransferase, APTT = activated partial thromboplastin time, AST = aspartate aminotransferase, INR = international normalised ratio, PK = pharmacokinetic, TSH = thyroid stimulating hormone.
a Clinical chemistry: 8.5 - 10 mL (including TSH); if ALT/AST tested separately – additional 4 mL.

17.3 Distribution of Patients Scenarios

Example Trials

In this section, we present simulated example trial results. This section is intended to clarify the adaptive design rules of the trial. In example 1, we walk through the steps of the trial and explain each of the rules. In the remaining examples, we present a table of the data at each interim analysis with the posterior summaries and showing the sequences of the trial results.

Example 1:

Interim	N	PBO	2	3	4	5	6
End of Part 1	36	2/18 (0.11)		10/18 (0.56)			

Abbreviations: PBO=placebo

Summary of Posterior Distribution

Interim	N	PBO	2	3	4	5	6
Pr (P_d>0.65)	36	--		0.19			

Abbreviations: PBO=placebo, P_d=conversion ratio at d mg/kg, *Pr*=probability

At the conclusion of Part 1, the observed rate of success on Dose 3 is 0.56. As this value is <0.65, Dose 2 is not opened. Dose 5 is opened at the start of Part 2. By rule, Dose 3 is closed at the start of Part 2. The one active dose and placebo will be randomised (2:1) for 18 more patients. The results after these 18 patients are:

Interim	N	PBO	2	3	4	5	6
End of Part 1	36	2/18 (0.11)		10/18 (0.56)			
Interim 1; Part 2	54	3/24 (0.13)				8/12 (0.67)	

Abbreviations: PBO=placebo

Summary of Posterior Distribution:

Interim	N	PBO	2	3	4	5	6
Pr (P_d>0.65)	54	--		0.03		0.50	

Abbreviations: PBO=placebo, P_d=conversion ratio at d mg/kg, *Pr*=probability

At the conclusion of the first IA in Part 2, Dose 5 has a 0.50 probability of being better than a 65% rate, which is between 0.10 and 0.90 and thus continues to be allocated. Dose 5 is the only active dose and thus randomisation is 2:1 for Dose 5 versus placebo for 18 new patients. The new results for these 18 new patients are:

Interim	N	PBO	2	3	4	5	6
End of Part 1	36	2/18 (0.11)		10/18 (0.56)			
Interim 1; Part 2	54	3/24 (0.13)				8/12 (0.67)	
Interim 2; Part 2	72	3/30 (0.10)				17/24 (0.71)	

Abbreviations: PBO=placebo

Summary of Posterior Distribution:

Interim	N	PBO	2	3	4	5	6
Pr (P_d>0.65)	72	--		0.03		0.69	

Abbreviations: PBO=placebo, P_d=conversion ratio at d mg/kg, *Pr*=probability

Dose 5 has a 0.69 posterior probability, which is >65% but is not greater than the threshold of 90% to stop for sufficient efficacy. The randomisation for the next 18 patients will continue to be 2:1 for Dose 5: placebo. The new results are:

Interim	N	PBO	2	3	4	5	6
End of Part 1	36	2/18 (0.11)		10/18 (0.56)			
Interim 1; Part 2	54	3/24 (0.13)				8/12 (0.67)	
Interim 2; Part 2	72	3/30 (0.10)				17/24 (0.71)	
Interim 3; Part 2	90	5/36 (0.14)				28/36 (0.78)	

Abbreviations: PBO=placebo

Summary of Posterior Distribution:

Interim	N	PBO	2	3	4	5	6
Pr (P_d>0.65)	90	--				0.94	

Abbreviations: PBO=placebo, P_d=conversion ratio at d mg/kg, *Pr*=probability

Dose 5 has 36 patients completing Day 2 assessments or terminated the study and the posterior probability that the rate is >65% is 0.94, which is larger than the cut-off of 0.90, so Dose 5 will be closed for sufficient success. Dose 4 will be opened for the last cohort of patients. The remaining enrolment period will be enrolled 2:1 for Dose 4 to placebo.

Interim	N	PBO	2	3	4	5	6
End of Part 1	36	2/18 (0.11)		10/18 (0.56)			
Interim 1; Part 2	54	3/24 (0.13)				8/12 (0.67)	
Interim 2; Part 2	72	3/30 (0.10)				17/24 (0.71)	
Interim 3; Part 2	90	5/36 (0.14)				28/36 (0.78)	
Final Data	108	6/42 (0.14)		10/18 (0.56)	8/12 (0.67)	28/36 (0.78)	

Abbreviations: PBO=placebo

Summary of Posterior Distribution:

Interim	N	PBO	2	3	4	5	6
Pr (P_d>0.65)	108	--		>0.999	>0.999	>0.999	

Abbreviations: PBO=placebo, P_d=conversion ratio at d mg/kg, *Pr*=probability

At the conclusion of the trial each of the Doses 3, 4, and 5, are deemed superior to placebo.

Example 2

Interim	N	PBO	2	3	4	5	6
End of Part 1	36	2/18 (0.11)		4/18 (0.22)			
Interim 1; Part 2	54	4/24 (0.17)				5/12 (0.42)	
Interim 2; Part 2	72	6/30 (0.20)					7/12 (0.58)
Interim 3; Part 2	90	7/36 (0.19)					16/24 (0.67)
Final Data	108	8/42 (0.19)		4/18 (0.22)		5/12 (0.42)	24/36 (0.67)

Abbreviations: PBO=placebo

Summary of Posterior Distribution:

Interim	N	PBO	2	3	4	5	6
Pr (P_d>0.65)	36			0.00			
Pr (P_d>0.65)	54					0.05	
Pr (P_d>0.65)	72						0.28
Pr (P_d>0.65)	90						0.53
Pr (P_d>P_0)	108			0.690		0.959	>0.999

Abbreviations: PBO=placebo, P_d=conversion ratio at d mg/kg, *Pr*=probability

Doses 5 and 6 are considered superior to placebo.

Example 3

Interim	N	PBO	2	3	4	5	6
End of Part 1	36	2/18 (0.11)		9/18 (0.50)			
Interim 1; Part 2	54	4/24 (0.17)				9/12 (0.75)	
Interim 2; Part 2	72	6/30 (0.20)				19/24 (0.79)	
Interim 3; Part 2	90	7/36 (0.19)			9/12 (0.75)		
Final Data	108	8/42 (0.19)		9/18 (0.50)	16/24 (0.67)	19/24 (0.79)	

Abbreviations: PBO=placebo

Summary of Posterior Distribution:

Interim	N	PBO	2	3	4	5	6
$Pr\ (P_d>0.65)$	36			0.08			
$Pr\ (P_d>0.65)$	54					0.72	
$Pr\ (P_d>0.65)$	72					0.91	
$Pr\ (P_d>0.65)$	90				0.72		
$Pr\ (P_d>P_0)$	108			0.994	>0.999	>0.999	

Abbreviations: PBO=placebo, P_d=conversion ratio at d mg/kg, *Pr*=probability

Doses 3, 4, and 5 are considered superior to placebo.

Example 4

Interim	N	PBO	2	3	4	5	6
End of Part 1	36	3/18 (0.17)		13/18 (0.72)			
Interim 1; Part 2	54	4/24 (0.17)	3/6 (0.50)			4/6 (0.67)	
Interim 2; Part 2	72	5/30 (0.20)	4/12 (0.33)			8/12 (0.67)	
Interim 3; Part 2	90	6/36 (0.17)				16/24 (0.67)	
Final Data	108	6/42 (0.14)	4/12 (0.33)	13/18 (0.72)		24/36 (0.67)	

Abbreviations: PBO=placebo

Summary of Posterior Distribution

Interim	N	PBO	2	3	4	5	6
Pr (P_d>0.65)	36			0.70			
Pr (P_d>0.65)	54		0.19			0.47	
Pr (P_d>0.65)	72		0.01			0.50	
Pr (P_d>0.65)	90					0.53	
Pr (P_d>P_0)	108		0.947	>0.999		>0.999	

Abbreviations: PBO=placebo, P_d=conversion ratio at d mg/kg, *Pr*=probability

Doses 2, 3, and 5 are considered superior to placebo.

Statistical Analysis Plan for Interventional Studies

Sponsor Name: Acesion Pharma ApS

Protocol Number: AP30663 - 2001
Protocol Title: A Double-Blind, Randomised, Placebo-Controlled, Parallel-Group Study of AP30663 Given Intravenously for Cardioversion in Patients with Atrial Fibrillation

Protocol Version and Date: Version 4.0, Final 23-Mar-2022

Syneos Health Project Code: 7000776

Authors: Franziska KOHL, Principal Biostatistician
Stephanie GAGNON, Senior Pharmacokineticist

Notice of Confidential and Proprietary Information:

The information contained in this document is confidential belonging to Acesion Pharma ApS. Acceptance of this document constitutes agreement by the recipient that no information contained herein will be published or disclosed without prior written authorization from an official of Acesion Pharma ApS. However, this document may be disclosed to appropriate Institutional Review Board and Ethics Committees or duly authorized representatives of a national regulatory authority under the condition that they are requested to keep it confidential. In the event of an actual or suspected breach of this obligation, Syneos Health should be notified promptly.

Revision History

Version #	Date (DD-Mmm-YYYY)	Document Owner	Revision Summary
0.1	08-Feb-2019	Fabienne Baelen	Initial Release Version
0.2	19-Feb-2019	Fabienne Baelen	Updated based on senior statistical review comments
0.3	26-Feb-2019	Fabienne Baelen	Updated based on senior statistical review comments
0.4	11-Mar-2019	Fabienne Baelen	Updated based on Internal review comments
0.5	19-Mar-2019	Fabienne Baelen	Updated based on Acesion review comments
0.6	27-Mar-2019	Fabienne Baelen	Upadted list of TFLs to be provided for Interim analysis
0.7	08-Apr-2019	Fabienne Baelen	Updated based on Internal review comments
0.8	07-May-2019	Fabienne Baelen	Updated based on Sponsor review comments
1.0	12-July-2019	Fabienne Baelen	Final version
2.0	19-Mar-2020	Fabienne Baelen	Updated after protocol amendment
3.0	06-Aug-2020	Fabienne Baelen	Updated after protocol amendment version 3.0 01-Jun-20
4.0	20-Feb-2023	Franziska Kohl	Updated after protocol amendment version 4.0 23-Mar-22 and decisions made during the BDRM (08Feb2023)

Table of Contents

Revision History 2
Approvals 3
1. Glossary of Abbreviations 8
2. Purpose 10
   2.1. Responsibilities 10
   2.2. Timings of Analyses 10
3. Study Objectives 11
   3.1. Primary Objective 11
   3.2. Secondary Objectives 11
   3.3. Exploratory Objectives 11
   3.4. Brief Description 11
   3.5. Patient Selection 12
      3.5.1. Inclusion Criteria 12
      3.5.2. Exclusion Criteria 12
   3.6. Determination of Sample Size 12
   3.7. Treatment Assignment & Blinding 13
   3.8. Administration of Study Medication 13
   3.9. Study Procedures and Flowchart 13
4. Endpoints 14
   4.1. Primary Efficacy Endpoint 14
   4.2. Secondary Efficacy Endpoints 14
   4.3. Exploratory Endpoints 14
   4.4. Pharmacokinetic Endpoints 14
   4.5. Safety Endpoints 14
5. Analysis Sets 15
   5.1. Enrolled Set 15
   5.2. Randomised Set 15
   5.3. Full Analysis Set 15

This document is confidential.

5.4. Per Protocol Set 15
5.5. Safety Set 15
5.6. Pharmacokinetic (PK) Set 15
5.7. Protocol Deviations 15
6. General Aspects for Statistical Analysis 17
6.1. General Methods 17
6.2. Key Definitions 17
6.2.1. Baseline Value 17
6.2.2. Last assessment 17
6.3. Missing Data 18
6.3.1. Missing Dates for prior or concomitant medications 18
6.3.2. Missing Dates for adverse event 18
6.4. Visit Windows 18
6.5. Pooling of Centres 19
6.6. Subgroups 19
7. Demographic, Other Baseline Characteristics and Medication 20
7.1. Patient Disposition and Withdrawals 20
7.2. Demographic and Other Baseline Characteristics 20
7.3. Medical History and Concomitant Diseases 21
7.4. Medication 21
7.4.1. Prior Medication 21
7.4.2. Concomitant Medication 21
8. Efficacy 22
8.1. Primary Efficacy Endpoint and Analysis 22
8.2. Secondary Efficacy Endpoint(s) and Analyses 22
8.2.1. Time to conversion from AF from start of infusion 22
8.2.2. Proportion of patients with relapse of AF within 5 min (IRAF) 23
8.2.3. Proportion of patients in SR 23
9. Analysis of Pharmacokinetics 24

This document is confidential.

9.1. PK Sampling Schedule 24
9.2. Derived and Imputed Data for PK Endpoint 24
9.2.1. Handling of Dropouts, Particularities, Missing Data or Data Below the Lower Limit of Quantification 25
9.3. Planned Statistical Models for PK Parameters and Concentrations 25
9.4. PK Interim Analyses....25
9.5. Deviation from Analyses Planned in Protocol....26
10. Safety....27
10.1. Extent of Exposure 27
10.2. Treatment Compliance....27
10.3. Adverse Events / Adverse Drug Reactions 27
10.4. Laboratory Evaluations 28
10.5. Vital Signs....28
10.6. ECG....29
10.7. Physical Examination....30
11. Interim Analyses....31
12. Changes from Analysis Planned in Protocol....32
13. Reference List 33
14. Programming Considerations 34
14.1. General Considerations 34
14.2. Table, Listing, and Figure Format 34
14.2.1. General 34
14.2.2. Headers....35
14.2.3. Display Titles....35
14.2.4. Column Headers 35
14.2.5. Body of the Data Display 36
14.2.6. Footnotes 38
15. Quality Control 39
16. Index of Tables....40
17. Index of Figures....43

This document is confidential.

18. Index of Listings 45
19. Appendices 46

1. Glossary of Abbreviations

AE	Adverse event
AERP	Atrial effective refractory period
AF	Atrial fibrillation
ALT	Alanine aminotransferase
APTT	Activated partial thromboplastin time
AST	Aspartate aminotransferase
AUC	Area under the concentration time curve
AV	Atrioventricular
BBB	Bundle branch block
BP	Blood pressure
bpm	Beats per minute
BUN	Blood urea nitrogen
Ca	Calcium
CABG	Coronary artery bypass graft
C_{max}	Observed peak concentration
CYP	Cytochrome P450
DC	Direct current
DMC	Data Monitoring Committee
ECG	Electrocardiogram
eCRF	Electronic Case Report Form
EDC	Electronic data capture
eGFR	Estimated glomerular filtration rate
EMA	European Medicines Agency
EU	European Union
FDA	Food and Drug Administration
GCP	Good clinical practice
GGT	Gamma-glutamyltransferase
GLP	Good Laboratory Practice
IC_{50}	Half maximal inhibitory concentration

This document is confidential.

ICD	Implantable cardioverter defibrillator
ICF	Informed consent form
ICH	International Council for Harmonisation
IEC	Independent ethics committee
INR	International normalised ratio
IRAF	Immediate relapse of atrial fibrillation
IWRS	Interactive web randomization system
Kel	Elimination rate constant
LV	Left ventricle
LVEF	Left ventricular ejection fraction
MedDRA	Medical Dictionary for Regulatory Activities
MTD	Maximum tolerated dose
NCA	Non-compartmental analysis
NOAEL	No observed adverse effect level
PCI	Percutaneous coronary intervention
PD	Pharmacodynamics
PK	Pharmacokinetic
QTc	Corrected QT interval
SAE	Serious adverse reaction
SAP	Statistical analysis plan
SD	Standard deviation
SOC	System organ class
SOP	Standard operating procedure
SR	Sinus rhythm
$t_{1/2}$	Terminal half-life
TEAE	Treatment-emergent adverse event
T_{max}	Time at which C_{max} occurs
TSH	Thyroid stimulating hormone
TTE	Transthoracic echo
WHO	World health organisation

2. Purpose

The purpose of this statistical analysis plan (SAP) is to ensure that the data listings, summary tables and figures which will be produced, and the statistical methodologies that will be used, are complete and appropriate to allow valid conclusions regarding the study objectives.

2.1. Responsibilities

Syneos Health will perform the statistical analyses and are responsible for the production and quality control of all tables, figures and listings. Pharmacokinetic analysis will also be carried out by Syneos Health.

2.2. Timings of Analyses

The study will be conducted in two parts (Parts 1 and 2): Part 1 of the study is a fixed randomisation placebo-controlled parallel design, and Part 2 will be an adaptive design conducted in one or more further doses of AP30663 vs Placebo.

In Part 1 of the study, up to 36 patients will be randomised in a 1:1 ratio to receive AP30663 at 3 mg/kg or matching placebo. An interim analysis (IA) will be conducted once the randomised patients in Part 1 have completed Day 2 assessments or terminated the study(see section 8.1 of the protocol).

An interim analysis (Part 1) including efficacy, PK/PD, safety and tolerability data will be conducted after all randomised patients have completed Day 2 assessments of the study, in order to determine the starting dose(s) for Part 2 of the study. For further information see section 11. An independent data monitoring committee (DMC) will be convened to review the accumulating unblinded efficacy (including ECG and Holter ECG), PK/PD, safety and tolerability data for the study at the time of the interim analysis.

During Part 2, IAs will occur when 18, 36 and 54 patients have been randomised to open-doses including placebo, and have completed Day 2 assessments or terminated the study.

For Part 2, the DMC will give their recommendations regarding continuation or early termination of the study. Decision rules will be based on the AF conversion rate; however the DMC will also have the option to stop a dose arm (or the study) due to unacceptable safety and tolerability (including but not limited to increase in QTcF interval and number and severity of local injection site reactions).

An unblinded team (unblinded statistician and unblinded programming team) will be responsible for generating and delivering unblinded TFLs to the DMC members. Tables that will be delivered for the interim analysis are detailed in Section 16.

This document is confidential.

3. Study Objectives

3.1. Primary Objective

The primary objective of the study is to demonstrate the efficacy of one or more dose levels of AP30663 on the basis of the ability to convert atrial fibrillation (AF) after intravenous administration.

3.2. Secondary Objectives

The secondary objectives of the study are as follows:

- To study the stability of rhythm control (immediate relapse of AF [IRAF], *i.e.* within 5 min after conversion from AF).
- To study the importance of AF duration with respect to the efficacy and safety of one or more dose levels of AP30663.
- To evaluate the safety and tolerability of one or more dose levels of AP30663.
- To study the relationship between systemic exposure and response, with special regard to the conversion from AF and the effect on QRS and QTcF.

3.3. Exploratory Objectives

The exploratory objectives of the study are as follows:

- To study demographic and echocardiographic variables, concomitant diseases, and concurrent medication with respect to the efficacy and safety of one or more dose levels of AP30663.
- To evaluate the PK of AP30663, including influence of dose, concomitant medication, concurrent diseases, and demographic variables.
- To explore the proportions of patients on AP30663 converting from AF and of patients randomised to placebo and converting at DC cardioversion.

3.4. Brief Description

This is a double-blind, randomised, placebo-controlled, parallel-group Phase 2a study of one or more dose levels of AP30663 for cardioversion in adult patients with AF.

The study will be conducted in two parts (Part 1 and 2): Part 1 of the study is a fixed randomisation placebo-controlled parallel design and Part 2 will be an adaptive design conducted in one or more further doses of AP30663 vs Placebo. The study is designed to identify the dose of AP30663 to be evaluated further in later-Phase research. Though the study investigates multiple doses of AP30663 versus placebo, the participating patients will be randomised to receive one dose of AP30663 (i.e. patients will not receive multiple dose levels).

This document is confidential.

Figure 1 Study Schema

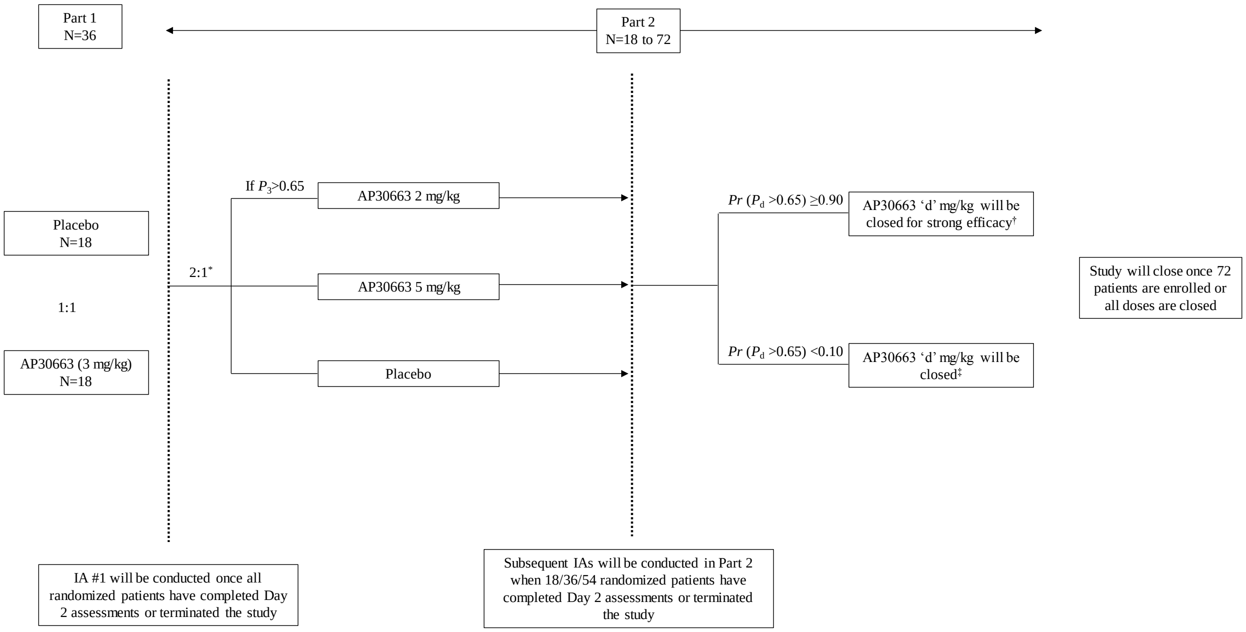

Abbreviations: N=number of patients enrolled, P_3=conversion ratio at 3 mg/kg, P_d=conversion ratio at d mg/kg, *P*r=probability.

*All active AP30663 arms will be randomised equally for each interim analysis; randomisation will occur cumulatively in 2:1 ratio to receive either AP30663 or placebo across all open AP30663 arms.

†If one level below this dose (d-1) has never been opened, then dose d-1 will be opened

‡If dose 'd' is the highest dose opened so far, and dose 'd' is <6 mg/kg, then dose 'd+1' will be opened.

3.5. Patient Selection

3.5.1. Inclusion Criteria

See section 8.3.2 of the protocol

3.5.2. Exclusion Criteria

See section 8.3.3 of the protocol

3.6. Determination of Sample Size

The study is a proof of concept, designed to allow for a considered testing of a range of doses of AP30663, compared with placebo. Based on the study design, the sample size for randomisation will range from up to 36 to up to 108. The total sample size is dependent on the AP30663 dose arms opened

on the decision of the DMC. A total of 4 IAs and a final analysis are included in the design, and so it is foreseen that the total number of randomised patients will be either up to 36, 54, 72, 90 or 108 patients to have a sufficient number of patients evaluable for AF conversion at each IA.

The adaptive design utilises the probability that the response rate at a Dose d is greater than a success rate of 65% ($P_d > 0.65$, where d is the dose arm of AP30663), based on a Bayesian modelling procedure.

3.7. Treatment Assignment & Blinding

For Part 1 of the study, eligible patients will be randomised 1:1 (approximately 36 patients randomised to AP30663 or placebo). An additional 18 to 72 patients will be randomised cumulatively in a 2:1 ratio to 1 or more doses of AP30663 (dependent on dose arms open at time of randomisation) or placebo in Part 2 using an interactive web randomisation system (IWRS).

Two separate lists will be utilised for the study, one for Part 1 and a separate one for Part 2. Both lists will be provided by a separate unblinded biostatistical team and provided to the IWRS.

Acesion Pharma, investigators, and patients will remain blinded to the study treatment allocation until the end of the study. The randomisation list will be kept secure from the study team, investigators, and patients throughout the conduct of the study and until unblinding is authorised by Acesion Pharma and the blinded lead study statistician.The DMC will be unblinded according to the DMC charter, and a separate unblinded statistical team will be assigned to provide the analyses to the DMC and Interim Analysis. Further details of blinded and unblinded personnel will be included in the DMC charter.

3.8. Administration of Study Medication

Study drugs will either be AP30663 or placebo.

Prior to the interim analysis (Part 1 of the study), patients randomised to the AP30663 arm will receive AP30663 at a dose of 3 mg/kg.

The starting AP30663 dose(s) for Part 2 of the study will be determined during DMC review of the interim analysis at the end of Part 1. In Part 2, patients randomised to AP30663 may receive one of the following AP30663 doses: 2, 4, 5, or 6 mg/kg dependent on the results seen for the decision criteria, and the subsequent dose recommended by the DMC.

Patients randomised to the placebo arm will receive matching placebo infusion.

3.9. Study Procedures and Flowchart

The schedule of the planned study assessments during Part 1 and Part 2 is in section 8.1.2 from the protocol.The planned study assessments during Part 2 are the same as those that are planned for Part 1.

This document is confidential.

4. Endpoints

4.1. Primary Efficacy Endpoint

The proportion of patients that have converted from AF within 90 min from the start of infusion and subsequently have no AF recurrence within 1 min of conversion from AF.

4.2. Secondary Efficacy Endpoints

- The time to conversion from AF from start of infusion.
- The proportion of patients with relapse of AF within 5 min (IRAF) following pharmacological or direct-current (DC) cardioversion.
- The proportion of patients in SR at 3 h ± 1 h after start of infusion.
- The proportion of patients in SR at 24 h ± 2 h after start of infusion.
- The proportion of patients in SR at 30 days ± 5 days after start of infusion.

4.3. Exploratory Endpoints

- Correlation of drug exposure (plasma Cmax and area under the concentration time curve [AUC]) and the time of conversion.
- Correlation of atrial size and conversion rate.
- Correlation of atrial size to relapse or failure.
- Correlation of gender and age and conversion rate.
- Correlation of duration of current AF, conversion rate and - sinus rhythm at some timepoint .

4.4. Pharmacokinetic Endpoints

The PK endpoints are as follows:

- Systemic exposure derived from the population PK model.
- Population PK model parameter estimates derived from plasma concentrations of AP30663.

Concentration-derived PK parameters of plasma AP30663 will be determined using 2 approaches:

• A static analysis based on plasma concentration will be performed using a non-compartmental analysis (NCA);

• PK parameters will also be derived using a non-linear mixed-effect modeling approach.

4.5. Safety Endpoints

- Adverse events (AEs), electrocardiogram (ECG) variables including significant arrhythmia, physical examination, vital signs, and laboratory evaluations.
- Changes in QTcF interval data over time.

This document is confidential.

5. Analysis Sets

For purposes of analysis, the following populations are defined for both Parts 1 and 2.

5.1. Enrolled Set

The Enrolled Set will include all patients who sign the ICF. Unless specified otherwise, this set will be used for patient listings and for summaries of patient disposition. This set will include screen failures.

5.2. Randomised Set

The Randomised Set will include all patients who signed the ICF and were subsequently randomised into the study (Screen failure are excluded), regardless of study treatment administration.

5.3. Full Analysis Set

The Full Analysis Set will serve as the primary population for the analysis of efficacy and will consist of all randomised participants who were administered double-blind study treatment and have an evaluable AF conversion status within 90 min from the start of infusion.

Patients will be analysed according to the randomised treatment.

5.4. Per Protocol Set

The Per-Protocol Set includes all patients from the Full Analysis Set who have been treated according to the protocol and fulfil the following criteria:

1. All inclusion/exclusion criteria satisfied according to the latest AP30663-2001 Protocol Version 4.0
2. Absence of relevant protocol violations with respect to factors likely to affect the efficacy of treatment where the nature of protocol violation will be defined before breaking the blind
3. Adequate study drug compliance, which will be determined before breaking the blind

Patients will be analysed according to the randomised treatment.

5.5. Safety Set

All randomised patients who were administered double-blind study treatment. Patients will be analysed according to the treatment received. The Safety Set will be used for all analyses of safety endpoints.

5.6. Pharmacokinetic (PK) Set

The PK Set will include all patients in the Safety Set who have at least one evaluable post-infusion drug concentration value.

5.7. Protocol Deviations

Important protocol deviations will be collected in the eCTMS. Each instance of a protocol deviation will be reviewed by the Sponsor and determined to be either major or minor before DBL.
The major protocol deviations include, but are not limited to the following (to be finalized before DBL):

- Eligibility deviations (Inclusion/Exclusion criteria)
- Improper reconstitution and administration of study medication
- Noncompliance with study medication (stop of drug infusion prior to 30 min)
- Noncompliance with study procedures if the consequence of noncompliance would compromise either the patient's safety and/or the study integrity, primary endpoint, and/or is not in line with GCP/ICH guidelines
- Use of prohibited concomitant therapies

Patients with any major protocol deviations that likely affect the efficacy of treatment will not be included in the PP population.

Number (%) of patients with at least one major protocol deviation will be included in a summary table, using the Randomised Set.

All protocol deviations will be listed for all patients in the Randomised Set, including their assignment of minor, major or critical, and the date the deviation occurred.

6. General Aspects for Statistical Analysis

6.1. General Methods

- SAS Version 9.4 or higher will be used for programming and production
- The statistical analysis based on plasma concentration will be performed using a non-compartmental analysis (NCA), the PK analyses will be performed by Syneos Health using Phoenix WinNonlin® (release 8.0 or higher) and all other analyses and summaries will be produced using Statistical Analysis System (SAS®) version 9.4 (or higher).
- PK parameters will be derived using a non-linear mixed-effect modeling approach, PK analyses will be performed by Syneos Health using NONMEM® version 7.4.
- Unless otherwise specified, summaries will be presented for each treatment and overall.
- Unless otherwise specified, continuous variables will be summarized using the number of observations (n), mean, standard deviation (SD), median, minimum, and maximum. Categorical variables will be summarized using number of observations (n), frequency and percentages of patients.
- All relevant patient data will be included in listings. All patients entered into the database will be included in patient data listings.
- <u>Handling of repeated measurements at the same visit</u>

  If measurements were repeated at the same scheduled visit, the value actually flagged as scheduled will be the

  ✓ Last non-missing repeated measurement, if visit is before start of infusion, and
  ✓ First non-missing repeated measurement, if visit is after start of infusion.

  Generally, only scheduled measurements will be used for statistical summaries and analysis. Unscheduled measurements will not used for analysis, but only listed.

6.2. Key Definitions

6.2.1. Baseline Value

Unless otherwise specified, the baseline value for any variable will be the last value taken prior to the infusion of the double-blind study treatment on Day 1. This includes pre-dose Day 1 assessments.

6.2.2. Last assessment

Unless otherwise specified, last assessment is defined as Day 30 visit or early discontinuation visit.

This document is confidential.

6.3. Missing Data

Unless otherwise specified, no imputation will be performed for missing data.

6.3.1. Missing Dates for prior or concomitant medications

For prior and concomitant medications with incomplete dates, the following rules will be used to impute start and/or stop dates for the purposes of determining whether a medication is prior or concomitant only.

- For start dates:
  -if day is missing the first day of the month will be used.
  -If day and month is missing, 1st of January will be used.
  -If the start date is missing completely, the date of infusion will be used.
  -If the stop date is complete and the imputed start date is after the actual stop data, then the start date will be imputed as the stop date.

- For stop dates:
  -if the day is missing then the last day of the month will be used.
  -If the day and month is missing, then 31st December will be used.
  -If the stop date is completely missing, then the date of infusion will be used. If the start date is complete and the imputed stop date is earlier than the actual start date, the stop date will be imputed as the start date.

6.3.2. Missing Dates for adverse event

If the adverse event start date is partially or completely missing, then the following rules will be used to impute the start date for the purposes of determining treatment emergence status only :

- If the whole start date is missing then the date of infusion will be used.
- <u>missing month</u> : if the AE has occurred the same year as the date of infusion, replace the missing month of onset by the month of date of infusion.

  If the AE has occurred the subsequent year after the year of date of infusion, replace the missing month of onset by January.

  If the AE has occurred the previous year of the year of date of infusion, replace the missing month of onset by December.
- <u>missing day</u> : replace it by ‘01’, and verify that the completed date is greater or equal to the date of infusion. If not, replace the incomplete date of onset by the date of infusion.

6.4. Visit Windows

Not applicable

6.5. Pooling of Centres

Not applicable

6.6. Subgroups

No subgroup for this study.

This document is confidential.

7. Demographic, Other Baseline Characteristics and Medication

7.1. Patient Disposition and Withdrawals

The following frequencies (number and percent) will be displayed in the patient disposition table, by treatment group and overall:

Number of patients enrolled;

Number of patients screen failure;

Number of patients randomised;

Number (%) of patients in the FAS set (Full Analysis Set see definition in section 5);

Number (%) of patients in the PP set (Per-Protocol Set, see definition in section 5);

Number (%) of patients in the PK set (Pharmacokinetic Set, see definition in section 5);

Number (%) of patients in the Safety set (see definition in section 5);

Number (%) of patients who completed the study;

Number (%) of patients who discontinued the study early, and reasons for discontinuation.

The analyses of disposition will be based on the Enrolled Set.The denominators for the percent calculations will be the number of patients in the Randomised Set.

Table for Patient Dispostion will be presented by cohort at each Interim analysis.

Patient completion status, date of completion/discontinuation and reason for discontinuation will be listed.

7.2. Demographic and Other Baseline Characteristics

Gender and child-bearing potential (female patients only), ethnicity will be summarized by the number and percentage of patients in each treatment group. Age (years) will be summarized using descriptive summary statistics.
Demographics parameters will be also presented by cohort at each Interim Analysis.
Data from transthoracic echo (TTE) will be presented using summary statistics. Height and baseline weight will be also summarized.

Duration of current AF episode (in hours) (derivation see section 8.1) and time since initial AF diagnosis (in years) will be summarized in addition.

Time since initial AF diagnosis (in years) = date of randomization – date of initial AF diagnosis (based on MHDECOD = ‘Atrial Fibrillation’)

In case of an incomplete date of initial AF diagnosis, the first day of the month will be used for a missing day, 1st January will be used for a missing day and month.

All demographic data will be presented in a data listing.

This document is confidential.

7.3. Medical History and Concomitant Diseases

Medical history will be coded to system organ class (SOC) and preferred term (PT) using Medical Dictionary for Regulatory Activities (MedDRA) version 21.1 or an updated version.

Data will be summarized by SOC and PT and sorted alphabetically. Conditions that are reported more than once for a given SOC and PT will be counted only once per patient on the PT level for each SOC

A corresponding data listing will be presented.

7.4. Medication

Section 6.3.1 describes how missing dates will be imputed for the purpose of determining prior/concomitant medication status.

7.4.1. Prior Medication

Prior and concomitant medications will be coded by the Anatomical Therapeutic Chemical (ATC) classification system according to the World Health Organization Drug Dictionary (WHODRUG), WHODrug Global B3 Sep 2018.

Prior medications will include medications started and stopped prior to the infusion date of the study drug.

For the summary tables, the count and percentage of patients under each anatomical therapeutic chemical (ATC) class level 2 and PT will be summarized by treatment group.
If a patient has taken prior medications more than once, the patient will be counted only once under any given drug class.

7.4.2. Concomitant Medication

Concomitant medications will include medication started on or after the infusion date of study drug. Medication that started before the infusion of study drug and continued on or after the infusion date will be considered as concomitant medication.

For the summary tables, the count and percentage of patients under each anatomical therapeutic chemical (ATC) class level 2 and PT will be summarized by treatment group.
If a patient has taken concomitant medications more than once, the patient will be counted only once under any given drug class.

A listing of prior and concomitant medications will be provided for all patients in the safety set. A flag will be included in the listing to distinguish prior and concomitant medications.

8. Efficacy

8.1. Primary Efficacy Endpoint and Analysis

The primary efficacy endpoint is defined as the proportion of patients who have converted from AF within 90 min from the start of infusion, and subsequently have no AF recurrence within 1 min of conversion from AF.

For the analysis of the primary endpoint (proportion of patients who have cardioversion within 90 min from the start of infusion) a Bayesian model will be utilised. The prior probability of success at a dose, d, is modelled with a P_d ~ Beta (1,1) prior (uniform) for each dose. Across both Parts 1 and 2 of the study, the potential doses (arms) in the trial are d = 0 (placebo), 2, 3, 4, 5, and 6 (all mg/kg dose units).

Let X_d be the number of successes and N_d be the number of observations at dose d. The posterior distribution is calculated for each dose independently using a Beta posterior distribution,

$$P_d \sim \text{Beta}\ (1 + X_d,\ 1 + N_d - X_d).$$

The adaptive design utilises the probability that the response rate at a dose is greater than a 65% success rate. This is ($P_d > 0.65$), for each dose d.

This modelling will occur for each interim analysis, and at the final analysis as primary method. At the conclusion of the study, each AP30663 dose will be considered to be superior to placebo, if the posterior probability of AP30663 dose having a higher success rate than placebo is greater than 0.95.

Further to the above, the primary endpoint will also be analysed by means of a logistic regression model. The model will include treatment and duration of current AF episode.

Duration of current AF episode (in hours) = date/time of randomisation – date/time of AF symptom onset

If no time of AF symptom onset is available, '00:00' will be used for the calculation of the duration.

All statistical tests will be 2-sided and conducted at the 5% significance level. All presented confidence intervals will be 2-sided 95% confidence intervals.

8.2. Secondary Efficacy Endpoint(s) and Analyses

8.2.1. Time to conversion from AF from start of infusion

The time to conversion from AF from start of infusion will be analysed using Kaplan-Meier methods. The number of patients that reached cardiac conversion within 90 mins of start of study drug infusion will be summarized using the median time to cardiac conversion along with the interquartile range (IQR) .

A Kaplan Meir plot stratified by treatment group will be presented. A logrank test will be used to compare treatment groups.

Time to conversion (in min) will be calculated using the formulae:

Time to conversion (in min) = (Time of conversion or censoring - time of start of infusion).

In case of AF persistence at 90 min after start of infusion, a direct-current (DC) electrical cardioversion is to be done within 180 min post-infusion start. Patients who have electrical cardioversion will be censored. The time of censoring will be 90 min.

8.2.2. Proportion of patients with relapse of AF within 5 min (IRAF)

The proportion of patients with relapse of AF within 5 min (IRAF) following pharmacological or DC cardioversion will be presented by treatment and analysed using a logistic regression model. The model will include treatment, and duration of current AF episode.

In addition the same analysis will be done for the proportion of patients with relapse of AF within 5 min (IRAF) following pharmacological cardioversion only.

8.2.3. Proportion of patients in SR

The proportion of patients in SR after start of infusion at different timepoints (3 h ± 1 h, 24 h ± 2 h and 30 days ± 5 days after start of infusion) will be presented by treatment group and analysed using a logistic regression model. The model wil include treatment and duration of current AF episode.

This document is confidential.

9. Analysis of Pharmacokinetics

All PK analyses will be conducted using the PK Set.

9.1. PK Sampling Schedule

Plasma samples will be taken at baseline (pre-infusion) and at the following time points after start of infusion: 5 min ± 1 min, 15 min ± 1 min, 25 min ± 1 min, 30 min - 1 min (the infusion will not be stopped before the 30-min plasma sample has been collected), 45 min ± 5 min, 1 h ± 5 min, 1 h 30 ± 5 min, 4 h ± 5 min, 8 h ± 5 min, and 24 h ± 2 hour (based on protocol version 4.0). In the case of conversion from atrial fibrillation during infusion, an additional sample will be taken immediately after conversion.

9.2. Derived and Imputed Data for PK Endpoint

For all sampling times, the actual sampling times will be calculated as the difference between the sample collection actual clock time and the actual clock time of start of the infusion of AP30663. The actual post-dose sampling times expressed in hours and rounded off to three decimal digits will be used to calculate the PK parameters, except for pre-dose samples occurring prior to infusion, which will always be reported as zero (0.000), regardless of the time difference.

For NCA approach, the following PK parameters including, but not limited to, will be determined for each patient based on plasma concentrations:

- $AUC_{0\text{-}0.5}$: Area under the concentration time curve from pre-dose concentration up to 30 min. $AUC_{0\text{-}0.5}$ includes the plasma drug concentration up to the end of infusion
- $AUC_{0\text{-}t}$: Area under the concentration time curve up to the last measurable concentration
- $AUC_{0\text{-}inf}$: Area under the concentration time curve from pre-dose through concentration to infinity (extrapolated), calculated as $AUC_{0\text{-}t} + C_t/K_{el}$, where C_t is the last observed non-zero concentration
- C_{max}: The observed peak drug concentration
- T_{max}: The time at which C_{max} occurs
- $t_{1/2}$: the terminal half-life value will be calculated using the equation ln2/ K_{el}, with K_{el} being the elimination rate constant
- K_{el}: Elimination rate constant. This parameter will be the negative of the estimated slope of the linear regression of the ln-transformed concentration versus time profile in the terminal elimination phase. At least 3 concentration points will be used in estimating Kel. The time point where ln-linear K_{el} calculation begins (K_{el} Lower), and the actual sampling time of the last quantifiable concentration used to estimate the K_{el} (K_{el} Upper) will be reported with the correlation coefficient from the linear regression to calculate K_{el}.

From the developed population PK model (Pop-PK), PK parameters like volume of distribution and clearance will be estimated. Additional PK parameters including, but not limited to, area under the curve at steady-state (AUC_{SS}) will be derived from the individual Bayes estimates from the pop-PK model. A separate analysis plan will be created to detail this approach.

This document is confidential.

9.2.1. Handling of Dropouts, Particularities, Missing Data or Data Below the Lower Limit of Quantification

Missing concentration data for all patients who are administered scheduled study treatment will be considered as non-informative missing and will not be imputed. No concentration estimates will be provided for missing sample values.

For NCA approach, plasma concentrations below the limit of quantification (BLQ) will be set to zero in the computation of mean concentration values; however, BLQ concentrations between 2 non-BLQ concentrations will be set to missing. For the computation of PK variables, the BLQ concentrations prior to the first measurable concentration will be set to zero and other BLQ concentrations will be set to missing.

Samples taken far outside the sampling windows may be excluded from by-time point summary statistics; this will be determined prior to database lock.

9.3. Planned Statistical Models for PK Parameters and Concentrations

The plasma concentration at each time point will be summarized as n, number and percentage BLQ, arithmetic and geometric mean, SD and arithmetic coefficient of variation (CV (%)) (calculated as 100*SD/mean), median, minimum and maximum

Descriptive statistics (n, arithmetic mean, SD, arithmetic CV (%), geometric mean, geometric CV(%), minimum, median and maximum) by treatment group will be calculated for all derived PK endpoints.

The following conventions will be used for the presentation of the descriptive statistics of PK parameters and of plasma concentrations:

PK Reporting Precision

Statistics	Degree of Precision
Minimum, Maximum	In the same precision as PK results, *i.e.* with 3 decimal places
Mean (arithmetic and geometric), Median	one more decimal place than the PK results, *i.e.* with 4 decimal places
Standard deviation	Two more decimal places than the PK results, *i.e.* with 5 decimal places
CV and Geometric CV	1 decimal place

9.4. PK Interim Analyses

After all patients in Part 1 have completed Day 2, corresponding to 24 hours after the stop of the infusion, an unblinded interim analysis (IA) will be performed using NCA and pop-PK model. The list of Topline Results for the interim analyses are described below for each method of derivation:

This document is confidential.

NCA:

- Mean (± SD) AP30663 Plasma Concentrations - Linear and semi-Log Scales – PK Set
- Overlay of Individual and Mean AP30663 Plasma Concentrations by Treatment - Linear and semi-Log Scales - PK Set

These figures will be also provided during Part 2 where IAs will occur when 18, 36 and 54 patients have been randomised to open-doses including placebo, and have completed Day 2 assessments or terminated the study.

Pop-PK model:

Interim results including average PK parameters determination will be provided using descriptive statistics and graphical evaluation.A summary report including conclusion/recommendation for the dose of Part 2 will be generated.

9.5. Deviation from Analyses Planned in Protocol

No deviation from PK analyses described in the Protocol is planned.

This document is confidential.

10. Safety

The population used for safety analyses will be the Safety Set (SS). Safety will be assessed on the basis of adverse event (AE) or adverse drug reactions (ADR) reports, clinical laboratory data, ECG parameters, physical examinations, and vital signs.

10.1. Extent of Exposure

Extent of exposure will be summarized by treatment group for duration of infusion .

Duration of infusion will be derived as:

Duration of infusion (in min)= (Stop Time of Study Drug Infusion- Start Time of Study Drug Infusion) – duration of infusion interruption

Extent of exposure (expected total volume to infuse and infused volume) will be summarized by treatment group.

10.2. Treatment Compliance

Number (%) of patients completing the full infusion treatment, number (%) of patients with infusion interruption and reasons for stopping will also be summarized.

10.3. Adverse Events / Adverse Drug Reactions

AEs will be coded using the Medical Dictionary for Regulatory Activities (MedDRA) version 21.1 or an updated version. Summaries will be presented by system organ class (SOC) and preferred term. Treatment-emergent AEs (TEAEs) are defined as any AE occurring or worsening on or after the first dose of study medication. Partial AE dates will be imputed.

The frequency and incidence of TEAEs will be presented by SOC and preferred term for each treatment group (number and percentage of patients experiencing at least one TEAE per preferred term as well as the number of observed events per preferred term; percentages will be based on the number of patients in the safety set).
The summaries presenting frequency of AEs by SOC and PT will be ordered by overall descending frequency of SOC and then, within a SOC, by overall descending frequency of PT.

Separate tables will be presented by severity and by relationship, and for SAEs, related TEAEs, and TEAEs leading to study discontinuation.
Relationships of 'very likely', 'probable' and 'possible' are to be considered as related to study drug for the summary tables. Relationships of 'unlikely' and 'unrelated' are to be considered not related to study drug.

In case of missing relationships, AEs will be considered as related.

All AEs will be presented in full in a comprehensive listing including patient number, treatment group, severity, seriousness, action taken, outcome, relationship to treatment, onset/stop, and duration. TEAEs will be flagged in the listings.

Details of SAEs and AEs leading to withdrawal will be listed separately.

10.4. Laboratory Evaluations

The haematology and clinical chemistry laboratory analyses will be performed at local laboratories. Reference ranges will be supplied by local laboratories and used by the investigator to assess the laboratory data for clinical significance and pathological changes. The following laboratory safety tests will be performed at the times outlined in the Study Procedures and Flowchart in Section 3.9.

Haematology

Haemoglobin, haematocrit, red blood cells (erythrocytes), white blood cells (leukocytes, total and differential), basophils, eosinophils, lymphocytes, monocytes, neutrophils, and platelets.

Clinical Chemistry

ALT, AST, bilirubin (direct, indirect, total), creatinine, gamma-glutamyltransferase, glucose (random), eGFR, urea (BUN), sodium, potassium, calcium, and magnesium.

Other Laboratory Variables

INR/APTT and TSH. These variables will be summarized and listed within clinical chemistry.

For all laboratory parameters, the following summary tables will be presented by visit and treatment for each test:

- Actual and changes from baseline for continuous variables
- Shift from baseline
- Number and percent of patients with clinically significant changes

All laboratory results in SI units will be included in data listings. Tests will be listed in alphabetical order. Abnormal lab values will be flagged in the listing .

10.5. Vital Signs

Vital signs will be recorded as indicated in the Study Procedures and Flowchart in Section 3.9. Blood Pressure, heart rate, and heart rhythm (from ECG) will be included and recorded in a standardized manner, *i.e.*, after the patient has rested in the (semi)supine position for 5 min and before blood sampling or laboratory tests. Vital signs measurements will be repeated in case of clinically significant abnormalities.

All vital signs (absolute values and change from baseline) will be summarized by visit, heart rhythm and timepoint for each treatment group.

All vital signs data will be listed chronologically by parameter and included in data listings.

10.6. ECG

A 12-lead digital ECG will be performed at the times outlined in the Study Procedures and Flowchart in Section 3.9 in a standardized manner (*i.e.* after the patient has rested in the [semi]supine position for at least 5 min).

The investigator will assess ECG abnormalities (*e.g.* ischemia, conduction abnormalities, and rhythm abnormalities) and define the ECG intervals as part of the screening of eligibility and according to the inclusion/exclusion criteria, and as part of an adverse event assessment.

12-lead digital ECG will be overread by the central ECG laboratory. The following measurements will be provided for all PK time points: RR, PR, QRS, QT, QTcF (Fridericia's correction of QT interval), heart rate, as well as T- and U-wave morphology classifications.

Bedside safety ECGs will be collected by the site, printed and reviewed by the investigator on site.

ECG intervals based on these safety ECGs will only be recorded if part of an adverse event.

A summary table for actual and change from baseline will be provided by treatment.

Results for 12-Lead ECGs will be summarized by the number and percentage within each category ("normal", "abnormal, not clinically significant", and "abnormal, clinically significant") .

Abnormal results for 12-Lead ECGs will be summarized as below:

- Absolute QTc interval prolongation:
  - QTc F interval > 450 ms
  - QTc F interval > 500 ms
- Change from baseline in QTc interval:
  - QTc F interval increases from baseline >30 ms
  - QTcF interval increases from baseline >60 ms

ECG data will be listed for all patients, and any clinically significant findings in ECG values will be flagged.

A cardiac Statistical Analysis Plan will be developed by Clario.

10.7. Physical Examination

At screening, weight and height will be recorded and a physical examination will be performed, followed by a brief physical examination at baseline (pre-infusion on Day 1) and Day 30. Any changes from the screening and baseline visit to Day 30 will be recorded. Full and brief physical examination will include the following assessments:

Assessment	Full physical examination	Brief physical examination
General appearance	x	x
Head, ears, eyes, nose, throat	x	-
Cardiovascular	x	x
Respiratory	x	x
Gastrointestinal	x	x
Dermatological	x	-
Neurological	x	-
Musculoskeletal	x	-
Lymphatic	x	-
Other (at the investigator’s discretion)	x	x

Body systems will be classified as “Normal”, “Abnormal Clinically Significant” and “Abnormal Not Clinically Significant”. Shift tables comparing physical examination results from baseline to last assessment will be presented by treatment.

Physical examination data will also be listed.

This document is confidential.

11. Interim Analyses

An interim analysis for efficacy (including ECG and Holter ECG), PK/PD, safety and tolerability will be conducted after all randomised patients have been recruited into Part 1 of the study and either completed Day 2 or terminated the study.

Interim Analysis at End of Part 1:

During the first IA the following will occur, regardless of the response seen for the AP30663 3 mg/kg dose:

- Dose 3 (3 mg/kg AP30663) will be stopped.
- Dose 5 (5 mg/kg AP30663 versus placebo) will be opened.

Additionally, the following decision will be made dependent on the response rate seen for 3 mg/kg AP30663:

- If the proportion of patients achieving a response under AP30663 3 mg/kg is > 0.65 then Dose 2 (2 mg/kg AP30663) will additionally be opened for Part 2 (in parallel to Dose 5).

Interim Analyses during Part 2:

Interim analyses will occur when 18, 36 and 54 randomised patients have completed Day 2 assessments or terminated the study in Part 2. At these IAs the following rules will be utilised:

a) If an open Dose d has at least 10 randomised patients having completed Day 2 assessments and satisfies: ($P_d > 0.65$) ≥ 0.90 then Dose d will be closed for sufficient efficacy response.
    - If the dose one level below this dose has never been opened, then dose d-1 is opened.

b) If an open Dose d has at least 10 randomised patients having completed Day 2 assessments and satisfies ($P_d > 0.65$) < 0.10 then Dose d will be closed.
    - If Dose d was the largest dose thus far open, and Dose d is < 6 mg/kg, then dose d + 1 will be opened.

The study will continue until all doses of AP30663 have been closed or the maximum of 72 patients during Part 2 (up to 108 patients total across Part 1 and Part 2) have been enrolled.

While the algorithm, and the DMC's decision for dose increase/decrease, is based on AF conversion rate only, the DMC will also convene to review safety and tolerability data at defined time points; review of this data may also lead to further decisions being made by the DMC.

This document is confidential.

12. Changes from Analysis Planned in Protocol

None

This document is confidential.

13. Reference List

None

This document is confidential.

14. Programming Considerations

All tables, figures, listings (TFLs), and statistical analyses will be generated using SAS for Windows, Release 9.4. (SAS Institute Inc., Cary, NC, USA) . Computer-generated table, listing and figure output will adhere to the following specifications.

The static analysis based on plasma concentration will be performed using a non-compartmental analysis (NCA), the PK analyses will be performed by Syneos Health using Phoenix WinNonlin® (release 8.0 or higher) and all other analyses and summaries will be produced using Statistical Analysis System (SAS®) version 9.4 (or higher).

PK analyses will be performed using NONMEM® version 7.4.

14.1. General Considerations

- One SAS program can create several outputs
- One output file can contain several outputs.
- Output files will be delivered in Rich Text Format (RTF) that can be manipulated in MSWord
- Numbering of TFLs will follow ICH E3 guidance

14.2. Table, Listing, and Figure Format

14.2.1. General

- All TFLs will be produced in landscape format on A4 paper size, unless otherwise specified.
- All TFLs will be produced using the Courier New font, size 8, which is the smallest acceptable point size for the Regulatory Authorities.
- The data displays for all TFLs will have a minimum blank 1-inch margin on all 4 sides.
- Headers and footers for figures will be in Courier New font, size 8, which is the smallest acceptable point size for the Regulatory Authorities.
- Legends will be used for all figures with more than 1 variable, group, or item displayed.
- TFLs will be in black and white (no color), unless otherwise specified.
- Specialized text styles, such as bolding, italics, borders, shading, and superscripted and subscripted text, will not be used in the TFLs, unless otherwise specified. On some occasions, superscripts 1, 2, or 3 may be used (see below).
- Only standard keyboard characters will be used in the TFLs. Special characters, such as non-printable control characters, printer-specific, or font-specific characters, will not be used. Hexadecimal-derived characters will be used, where possible, if they are appropriate to help

display math symbols (*e.g.*, μ). Certain subscripts and superscripts (*e.g*., cm2, Cmax) will be employed on a case-by-case basis.

- Mixed case will be used for all titles, footnotes, column headers, and programmer-supplied formats, as appropriate.

14.2.2. Headers

- All output should have the following header at the top left of each page:
- Acesion Pharma ApS

  Protocol: AP30663 - 2001
- Draft/Final Run *<date>*
- All output should have Page n of N at the top or bottom right corner of each page. TFLs are internally paginated in relation to the total length (*i.e.*, the page number should appear sequentially as page n of N, where N is the total number of pages in the table).
- The date/time of the generation of the output should appear along with the program name as a footer on each page.

14.2.3. Display Titles

- Each TFL is identified by the designation and a numeral. (*i.e.*, Table 14.1.1). ICH E3 numbering.A decimal system (x.y and x.y.z) are used to identify TFLs with related contents. The title is centered. The analysis set are identified on the line immediately following the title. The title and table designation are single spaced. A solid line spanning the margins will separate the display titles from the
- Column headers. There will be 1 blank line between the last title and the solid line.

Table x.y.z
First Line of Title
Second Line of Title if Needed
FAS Analysis Set

14.2.4. Column Headers

- Column headings are displayed immediately below the solid line described above in initial upper-case characters.
- In the case of efficacy tables, the variable (or characteristic) column will be on the far left followed by the treatment group columns and total column (if applicable). P-values may be presented under the total column or in separate p-value column (if applicable). Within-treatment comparisons may have p-values presented in a row beneath the summary statistics for that treatment.

This document is confidential.

- For numeric variables, include "unit" in column or row heading when appropriate.
- Analysis set sizes will be presented for each treatment group in the column heading as (N=xx) (or in the row headings, if applicable). This is distinct from the 'n' used for the descriptive statistics representing the number of patients in the analysis set.
- The order of treatments in the tables and listings will be Placebo first in the case of placebo controlled studies and Active comparators first in the case of active comparator trials, followed by a total column (if applicable).

14.2.5. Body of the Data Display

14.2.5.1. *General Conventions*

Data in columns of a table or listing are formatted as follows:

- Alphanumeric values are left-justified;
- Whole numbers (*e.g.*, counts) are right-justified; and
- Numbers containing fractional portions are decimal aligned.

14.2.5.2. *Table Conventions*

- Units will be included where available
- If the categories of a parameter are ordered, then all categories between the maximum and minimum category are presented in the table, even if n=0 for all treatment groups in a given category that is between the minimum and maximum level for that parameter. For example, the frequency distribution for symptom severity would appear as:

Severity Rating	N
severe	0
moderate	8
mild	3

Where percentages are presented in these tables, zero percentages will not be presented and so counts of 0 will be presented as 0 and not as 0 (0%).

- If the categories are not ordered (*e.g.*, Medical History, Reasons for Discontinuation from the Study, etc.), then only those categories for which there is at least 1 patient represented in 1 or more groups are included.
- An Unknown or Missing category are added to each parameter for which information is not available for 1 or more patient.

- Unless otherwise specified, the estimated mean and median for a set of values are printed out to 1 more significant digit than the original values, and standard deviations are printed out to 2 more significant digits than the original values. The minimum and maximum should report the same significant digits as the original values. For example, for systolic blood pressure:

N	XX
Mean	XXX.X
Std Dev	X.XX
Median	XXX.X
Minimum	XXX
Maximum	XXX

- P-values are output in the format: "0.xxx", where xxx is the value rounded to 3 decimal places. Every p-value less than 0.001 will be presented as <0.001. If the p-value are less than 0.0001, then present as <0.0001. If the p-value is returned as >0.999, then present as >0.999

- Percentage values are printed to one decimal place, in parentheses with no spaces, one space after the count (*e.g.*, 7 (12.8), 13 (5.4)). Unless otherwise noted, for all percentages, the number of patients in the analysis set for the treatment group who have an observation will be the denominator. Percentages after zero counts should not be displayed and percentages equating to 100% are presented as 100%, without decimal places.

- The percentage of of patients is normally calculated as a proportion of the number of patients assessed in the relevant treatment group (or overall) for the analysis set presented. However, careful consideration is required in many instances due to the complicated nature of selecting the denominator, usually the appropriate number of patients exposed. Describe details of this in footnotes or programming notes.

- For categorical summaries (number and percentage of patients) where a patient can be included in more than one category, describe in a footnote or programming note if the patient are included in the summary statistics for all relevant categories or just 1 category and the criteria for selecting the criteria.

- Where a category with a subheading (such as system organ class) has to be split over more than one page, output the subheading followed by "(cont)" at the top of each subsequent page. The overall summary statistics for the subheading should only be output on the first relevant page.

14.2.5.3. *Listing Conventions*

- Listings will be sorted for presentation in order of treatment groups as above, patient number, visit/collection day, and visit/collection time.

- Dates are printed in SAS DATE9.format ("ddMMMyyyy": 01JUL2000). Missing portions of dates are represented on patient listings as dashes (--JUL2000).

- All observed time values are to be presented using a 24-hour clock HH:MM or HH:MM:SS format (*e.g*., 11:26:45, or 11:26). Time will only be reported if it was measured as part of the study.

- Units will be included where available

14.2.5.4. *Figure Conventions*

- Unless otherwise specified, for all figures, study visits will be displayed on the X-axis and endpoint (*e.g.*, treatment mean change from Baseline) values will be displayed on the Y-axis.

14.2.6. Footnotes

- A solid line spanning the margins will separate the body of the data display from the footnotes.

- All footnotes will be left justified with single-line spacing immediately below the solid line underneath the data display.

- Footnotes should always begin with “Note:” if an informational footnote, or 1, 2, 3, etc. if a reference footnote. Each new footnote should start on a new line, where possible.

- Patient specific footnotes are avoided, where possible.

- Footnotes will be used sparingly and add value to the table, figure, or listing. If more than six lines of footnotes are planned, then a cover page is strongly recommended to be used to display footnotes, and only those essential to comprehension of the data will be repeated on each page.

- The last line of the footnote section will be a standard source line that indicates the name of the program used to produce the data display, date/time the program was run, and the listing source (*i.e.*, ‘Program : myprogram.sas   Listing source: 16.x.y.z’).

15. Quality Control

SAS programs are developed to produce output such as analysis data sets, summary tables, data listings, figures or statistical analyses. An overview of the development of programs is detailed in Syneos Health SOP Developing Statistical Programs (3907).

Syneos Health SOPs Developing Statistical Programs (3907) and Conducting the Transfer of Biostatistical Deliverables (3908) describes the quality control procedures that are performed for all SAS programs and output. Quality control is defined here as the operational techniques and activities undertaken to verify that the SAS programs produce the output by checking for their logic, efficiency and commenting and by review of the produced output."

This document is confidential.

16. Index of Tables

Table Number	Name	Analysis Set	Delivered for Interim analysis/DMC
Table 14.1.1.1	Patient Disposition	Enrolled Set	
Table 14.1.1.2	Patient Disposition – Cohort only	Enrolled Set	Only for IA
Table 14.1.2.	Major Protocol Deviations	Randomised Set	
Table 14.1.3.1.1	Demographics	Safety Set	✓
Table 14.1.3.1.2	Demographics – Cohort only	Safety Set	Only for IA
Table 14.1.3.2	Baseline Characteristics	Safety Set	
Table 14.1.3.3	Medical /Surgical History	Safety Set	
Table 14.1.4.1	Prior Medications	Safety Set	
Table 14.1.4.2	Concomitant Medications	Safety Set	
Table 14.1.5.1	Exposure to Study Treatment	Safety Set	✓
Table 14.1.5.2	Compliance with Study Treatment	Safety Set	
Table 14.2.1.1.1	Conversion from AF within 90 Minutes from Start of Infusion : Summary of Response Rate, Bayesian Model	Full Analysis Set	✓
Table 14.2.1.1.2	Conversion from AF within 90 Minutes from Start of Infusion : Summary of Response Rate, Bayesian Model	Per Protocol Set	
Table 14.2.1.1.3	Conversion from AF within 90 Minutes from Start of Infusion : Summary of Response Rate, Logistic Regression	Full Analysis Set	✓
Table 14.2.1.1.4	Conversion from AF within 90 minutes from start of infusion : Summary of Response Rate, Logistic Regression	Per Protocol Set	
Table 14.2.2.1.1	Time to Conversion from AF – Descriptive Statistics, Kaplan-Meier Analysis	Full Analysis Set	✓
Table 14.2.2.1.2	Time to Conversion from AF – Descriptive Statistics, Kaplan-Meier Analysis	Per Protocol Set	
Table 14.2.2.2.1	Relapse of AF within 5 min following Pharmacological or DC Conversion – Summary of relapse rate - Logistic Regression	Full Analysis Set	
Table 14.2.2.2.2	Relapse of AF within 5 min following pharmacological or DC conversion – Summary of relapse rate	Per Protocol Set	

This document is confidential.

Table Number	Name	Analysis Set	Delivered for Interim analysis/DMC
Table 14.2.2.2.3	Relapse of AF within 5 min following Pharmacological Conversion – Summary of relapse rate - Logistic Regression	Full Analysis Set	
Table 14.2.2.2.4	Relapse of AF within 5 min following Pharmacological Conversion – Summary of relapse rate - Logistic Regression	Per Protocol Set	
Table 14.2.2.3.1	Proportion of Patients in SR after Start of Infusion at Different Timepoints - Logistic Regression	Full Analysis Set	✓
Table 14.2.2.3.2	Proportion of Patients in SR after Start of Infusion at Different Timepoints - Logistic Regression	Per Protocol Set	
Table 14.2.3.1	Descriptive Statistics of Plasma Concentration over Time by Treatment	PK Set	
Table 14.2.3.2	Summary of Pharmacokinetic Parameters – NCA Approach	PK Set	
Table 14.3.1.1	Overall Summary of Treatment-Emergent Adverse Events	Safety Set	✓
Table 14.3.1.2	Treatment-Emergent Adverse Events by System Organ Class and Preferred Term	Safety Set	
Table 14.3.1.3	Treatment-Emergent Adverse Events Related to Study Treatment	Safety Set	✓
Table 14.3.1.4	Serious Treatment-Emergent Adverse Events by System Organ Class and Preferred Term	Safety Set	✓
Table 14.3.1.5	Treatment-Emergent Adverse Events Leading to Study Discontinuation by System Organ Class and Preferred Term	Safety Set	
Table 14.3.1.6	Treatment-Emergent Adverse Events by System Organ Class, Preferred Term, and Maximum Severity	Safety Set	
Table 14.3.4.1.1.1	Summary statistics of Laboratory Data: Haematology	Safety Set	
Table 14.3.4.1.1.2	Change from Baseline in Laboratory Data: Haematology	Safety Set	
Table 14.3.4.1.1.3	Laboratory Data: Haematology Relationship to Reference Range – Shift from Baseline	Safety Set	

Table Number	Name	Analysis Set	Delivered for Interim analysis/DMC
Table 14.3.4.1.1.4	Laboratory Data: Haematology Clinical Significance	Safety Set	
Table 14.3.4.1.2.1	Summary statistics of Laboratory Data: Clinical Chemistry	Safety Set	
Table 14.3.4.1.2.2	Change from Baseline in Laboratory Data: Clinical Chemistry	Safety Set	
Table 14.3.4.1.2.3	Laboratory Data: Clinical Chemistry Relationship to Reference Range – Shift from Baseline	Safety Set	
Table 14.3.4.1.2.4	Laboratory Data: Clinical Chemistry Clinical Significance	Safety Set	
Table 14.3.4.2.1	Summary of Vital Signs	Safety Set	
Table 14.3.4.2.2	Change from Baseline in Vital Signs	Safety Set	✓
Table 14.3.4.3.1	Summary of 12-Lead ECG Parameters	Safety Set	
Table 14.3.4.3.2	Change from Baseline in 12-Lead ECG Parameters	Safety Set	✓
Table 14.3.4.3.3	12-Lead ECG Clinical Significance	Safety Set	✓
Table 14.3.4.3.4	12-Lead ECG QTc Intervals: Categorical Analysis	Safety Set	✓
Table 14.3.4.4	Brief Physical Examination - Shift from Baseline	Safety Set	

This document is confidential.

17. Index of Figures

Figure Number	Figure Title	Delivered for Interim analysis/DMC
Figure 14.2.2.1.3	Kaplan-Meier curve for Time to Conversion from AF – Full analysis set	
Figure 14.2.3.3	Mean (±SD) Plasma Concentration - Linear and Semi-Log Scales - PK Set	✓ For interim analysis this graph will be provided by the PK group
Figure 14.2.3.4	Overlay of Individual and Mean AP30663 Plasma Concentrations by Treatment - Linear and Semi-Log Scales - PK Set	✓ For interim analysis this graph will be provided by the PK group
Figure 14.2.3.5	Scatter plot of Plasma Concentration Immediately After Conversion versus Time to Conversion – PK set	
Figure 14.2.3.6	Mean (±SD) Cmax vs. AP30663 Dose Level - PK Set	
Figure 14.2.3.7	Mean (±SD) AUC0-t vs. AP30663 Dose Level - PK Set	
Figure 14.2.3.8	Mean (±SD) AUC0-0.5 vs. AP30663 Dose Level - PK Set	
Figure 14.2.3.9	Scatter Plot of Cmax vs. Time to Conversion - PK Set	
Figure 14.2.3.10	Scatter Plot of AUC0-t vs. time to conversion- PK Set	
Figure 14.2.3.11	Scatter Plot of AUC0-0.5 vs. Time to Conversion - PK Set	
Figure 14.2.3.12	Bar chart of conversion rate vs. Age – Full analysis set	
Figure 14.2.3.13	Bar chart of conversion rate vs. Gender – Full analysis set	
Figure 14.2.3.14	Bar chart of conversion rate vs. Left Atrial Dimension/Diameter (Anterior Posterior, End Systolic)– Full analysis set	
Figure 14.2.3.15	Bar chart of Conversion Rate vs. Duration of Current AF episode– Full Analysis Set	

This document is confidential.

Figure Number	Figure Title	Delivered for Interim analysis/DMC
Figure 14.2.3.16	Bar Chart of Conversion Rate vs. Treatment Group – Full Analysis Set	

18. Index of Listings

Table Number	Name	Analysis Set	Delivered for Interim analysis/DMC
Listing 16.2.1.1	Completion of Study/Withdrawal of Study	Enrolled Set	
Listing 16.2.2	Protocol Deviations	Randomised Set	
Listing 16.2.3.1	Inclusion/Exclusion Criteria not met	Randomised Set	
Listing 16.2.3.2	Patients Excluded from Analysis Set	Randomised Set	
Listing 16.2.4.1	Demographics	Safety Set	
Listing 16.2.4.2	Baseline Characteristics	Safety Set	
Listing 16.2.4.3	Medical/Surgical History	Safety Set	
Listing 16.2.4.4	Prior and Concomitant Medications	Safety Set	
Listing 16.2.5.1	Study Drug Administration	Safety Set	
Listing 16.2.6.1	Efficacy : Conversion	Full Analysis Set	
Listing 16.2.7.1	Adverse Events	Safety Set	
Listing 16.2.7.2	Serious Adverse Events	Safety Set	
Listing 16.2.7.3	Adverse Events Leading to Treatment Discontinuation	Safety Set	
Listing 16.2.8.1.1	Haematology	Safety Set	
Listing 16.2.8.1.2	Clinical chemistry	Safety Set	
Listing 16.2.8.2.	Vital Signs	Safety Set	✓ Based on Randomised Set
Listing 16.2.8.3.	12-Lead ECG	Safety Set	✓ Based on Randomised Set
Listing 16.2.8.4	Physical Examination	Safety Set	
Listing 16.2.8.5	Plasma concentrations	PK Set	
Listing 16.2.8.6	Pharmacokinetics Parameters – NCA Approach	PK SET	

This document is confidential.

19. Appendices

APPENDIX I SAMPLE SAS CODE IN STATISTICAL ANALYSIS

1) Sample Code for Efficacy Analysis

The logistic regression will be used to analyze all efficacy endpoints relating to proportions

The following is an example of the SAS codes used to perform the analysis of this LOGISTIC model.

```
************************************************************************
* SAS Codes: LOGISTIC model
* Variables in the model:
* resp = proportion of patients who have converted from AF within 90 mn from the start of infusion
* Duration of current AF episode = Dur
*trtp = treatment planned
************************************************************************;
proc logistic data=eff descending;
class trtp (param=ref ref='Placebo');
model resp (event=”Yes”)= trtp dur;

contrast 'Pairwise'  trt 1 -1 0 /estimate=exp;
contrast 'Pairwise'  trt 1 0 -1 /estimate=exp;

ods output Contrastestimate=est;
run;
```

A Kaplan-Meier analysis was used for time to conversion. An example of the SAS code to perform this analysis is below.

```
************************************************************************
* SAS Code: Kaplan-Meier Analysis
* Variables in the model:
* timeconv = The time to conversion from AF from start of infusion
* Censor =  censor variable (0=patients with conversion, 1=censored patients, no pharmacological conversion)
*trtp = treatment planned
************************************************************************;
proc lifetest data=dataset plot;
```

```
time timeconv*censor(1);
strata trtp/ test=(logrank) diff=control('Placebo');
ods output quartiles/quartile
run;
```

2) Sample Code for graph

```
*****************************************************************************
* SAS Code: Graph Concentration response relationship:
 QTcF versus AP30663 plasma concentrations
* PCTRESN = Plasma concentration
* AVAL = QTcF
****************************************************************************;

proc sgplot data=PKPD;
reg x=PCSTRESN y=AVAL /CLM alpha=.05;
refline 0/axis=y;
label PCSTRESN="Plasma concentration (unit)";
run;
```
