# Supplementary material for: Inhibition of the KCa2 potassium channel in atrial fibrillation: a randomized phase 2 trial
Source: Nat Med. 2023 Dec 13;30(1):106–11. doi: 10.1038/s41591-023-02679-9 (PMC10803288; doi:10.1038/s41591-023-02679-9)
Supplement: Supplementary file 2 — Reporting Summary [file 41591_2023_2679_MOESM2_ESM.pdf]

Corresponding author(s): Anders Gaarsdal Holst

Last updated by author(s): Oct 22, 2023

Reporting Summary

Nature Portfolio wishes to improve the reproducibility of the work that we publish. This form provides structure for consistency and transparency in reporting. For further information on Nature Portfolio policies, see our Editorial Policies and the Editorial Policy Checklist.

Statistics

For all statistical analyses, confirm that the following items are present in the figure legend, table legend, main text, or Methods section.

n/a	Confirmed	
☐	☒	The exact sample size (*n*) for each experimental group/condition, given as a discrete number and unit of measurement
☒	☐	A statement on whether measurements were taken from distinct samples or whether the same sample was measured repeatedly
☐	☒	The statistical test(s) used AND whether they are one- or two-sided<br>*Only common tests should be described solely by name; describe more complex techniques in the Methods section.*
☐	☒	A description of all covariates tested
☐	☒	A description of any assumptions or corrections, such as tests of normality and adjustment for multiple comparisons
☐	☒	A full description of the statistical parameters including central tendency (e.g. means) or other basic estimates (e.g. regression coefficient) AND variation (e.g. standard deviation) or associated estimates of uncertainty (e.g. confidence intervals)
☒	☐	For null hypothesis testing, the test statistic (e.g. *F*, *t*, *r*) with confidence intervals, effect sizes, degrees of freedom and *P* value noted<br>*Give P values as exact values whenever suitable.*
☐	☒	For Bayesian analysis, information on the choice of priors and Markov chain Monte Carlo settings
☐	☒	For hierarchical and complex designs, identification of the appropriate level for tests and full reporting of outcomes
☒	☐	Estimates of effect sizes (e.g. Cohen's *d*, Pearson's *r*), indicating how they were calculated

Our web collection on statistics for biologists contains articles on many of the points above.

Software and code

Policy information about availability of computer code

Data collection	Data was collected through the electronic data capture system Medidata Classic Rave 2019-2023
Data analysis	SAS Version 9.4 or higher and R version 3.5.2 or higher was used for analyses.

For manuscripts utilizing custom algorithms or software that are central to the research but not yet described in published literature, software must be made available to editors and reviewers. We strongly encourage code deposition in a community repository (e.g. GitHub). See the Nature Portfolio guidelines for submitting code & software for further information.

Data

Policy information about availability of data

All manuscripts must include a data availability statement. This statement should provide the following information, where applicable:

- Accession codes, unique identifiers, or web links for publicly available datasets
- A description of any restrictions on data availability
- For clinical datasets or third party data, please ensure that the statement adheres to our policy

Data originating from the trial is considered commercially sensitive and as such, is not publicly available. To the extent that current legislation allows this, Tthe authors will provide access to individual-deidentified participant-level data that underlie the data presented in this paper, to researchers who provide a methodologically sound proposal for academic purposes to interpret, verify and extend research in the article that does not violate intellectual property or

confidentiality obligations beginning 12 months after article publication. Researchers should contact the corresponding author when applying for data access. Use of data will be restricted to the agreed upon purpose.

Research involving human participants, their data, or biological material

Policy information about studies with human participants or human data. See also policy information about sex, gender (identity/presentation), and sexual orientation and race, ethnicity and racism.

Reporting on sex and gender	Subjects self-reported sex. No analyses by sex have been reported due to the limited sample size making such subgroup analyses difficult, if not impossible to interpret.
Reporting on race, ethnicity, or other socially relevant groupings	No data on race was collected in the trial and all subjects self-reported to be "Not Hispanic or Latino". No analyses by ethnicity was performed in the trial.
Population characteristics	Reported in Table 1
Recruitment	Subjects were recruited through hospitals in Hungary and Denmark. Subjects were required to have an recent onset episode of atrial fibrillation and were identified mostly in emergency rooms or through collaboration with general practitioners when they presented with an acute episode of atrial fibrillation. Subjects were approached by a health care professional and asked if they would be interested in participating in the trial.
Ethics oversight	The trial protocol was approved by the following ethics committees: Den Videnskabsetiske Komité, Region Sjælland, Alléen 15, 4180 Sorø, Denmark; Egészségügyi Tudományos Tanács Klinikai Farmakológiai Etikai Bizottsága, Széchenyi István tér 7-8, Budapest, Hungary.

Note that full information on the approval of the study protocol must also be provided in the manuscript.

Field-specific reporting

Please select the one below that is the best fit for your research. If you are not sure, read the appropriate sections before making your selection.

☒ Life sciences ☐ Behavioural & social sciences ☐ Ecological, evolutionary & environmental sciences

For a reference copy of the document with all sections, see nature.com/documents/nr-reporting-summary-flat.pdf

Life sciences study design

All studies must disclose on these points even when the disclosure is negative.

Sample size	The sample size was selected based on a similar previous trial (doi:10.1016/J.JACC.2004.09.021), and no formal sample size calculations were made.
Data exclusions	Subjects were excluded from the primary analysis if they did not fulfill the following pre-specified definition of the full analysis set: randomised participants who were administered double-blind study treatment and have an evaluable AF conversion status within 90 min from the start of infusion.<br>The exclusion was done to ensure that only participants that could contribute data to the primary analysis were included in this.<br>No data points were excluded in the analysis.
Replication	As this was a stand-alone clinical trial no replication was performed.
Randomization	Randomization was performed with the use of a computer-generated random-sequence and interactive voice- and web-response system
Blinding	The trial was double-blind, meaning that both subjects and investigators were blinded to treatment allocation.

Reporting for specific materials, systems and methods

We require information from authors about some types of materials, experimental systems and methods used in many studies. Here, indicate whether each material, system or method listed is relevant to your study. If you are not sure if a list item applies to your research, read the appropriate section before selecting a response.

Materials & experimental systems

n/a	Involved in the study
☒	☐ Antibodies
☒	☐ Eukaryotic cell lines
☒	☐ Palaeontology and archaeology
☒	☐ Animals and other organisms
☐	☒ Clinical data
☒	☐ Dual use research of concern
☒	☐ Plants

Methods

n/a	Involved in the study
☒	☐ ChIP-seq
☒	☐ Flow cytometry
☒	☐ MRI-based neuroimaging

Clinical data

Policy information about clinical studies

All manuscripts should comply with the ICMJE guidelines for publication of clinical research and a completed CONSORT checklist must be included with all submissions.

Clinical trial registration	The trial was pre-registered in EudraCT: number 2018-004445-17. Later, it was also registered in ClinicalTrials.gov: number NCT04571385
Study protocol	Available in the Supplementary Appendix
Data collection	The trial was conducted at 10 hospital sites in Hungary and Denmark and patients were randomized between September 24, 2019, and December 9, 2022. Data was entered by site personal into an electronic data capture system (Medidate Rave Classic) and verified by sponsor monitors based on source documents.
Outcomes	The primary and secondary endpoints were prespecified in the trial protocol and in the Eudra CT and Clinicaltrials.gov public trial registries. The primary endpoint of cardioversion within 90 minutes was assessed by the investigator and verified centrally based on Holter monitoring read by a core laboratory. The secondary efficacy outcomes were assessed by the investigator and, if Holter monitoring was available at the assessment time (Holter monitoring was done for at least 8 and up to 24 hours after infusion start), this was used to verify the assessment.
